# Heterocyclic Diaryliodonium-Based Inhibitors of Carbapenem-Resistant Acinetobacter baumannii

**DOI:** 10.1128/spectrum.04773-22

**Published:** 2023-03-28

**Authors:** Pooja Kumari, Grace Kaul, T. Anand Kumar, Abdul Akhir, Manjulika Shukla, Suraj Sharma, Siddhesh S. Kamat, Sidharth Chopra, Harinath Chakrapani

**Affiliations:** a Department of Chemistry, Indian Institute of Science Education and Research Pune, Pune, Maharashtra, India; b Academy of Scientific and Innovative Research (AcSIR), Ghaziabad, India; c Division of Molecular Microbiology and Immunology, CSIR-Central Drug Research Institute, Lucknow, Uttar Pradesh, India; d Department of Biology, Indian Institute of Science Education and Research Pune, Pune, Maharashtra, India; University of Guelph College of Biological Science

**Keywords:** *Acinetobacter*, critical pathogens, drug discovery, drug resistance

## Abstract

Finding new therapeutic strategies against Gram-negative pathogens such as Acinetobacter baumannii is challenging. Starting from diphenyleneiodonium (dPI) salts, which are moderate Gram-positive antibacterials, we synthesized a focused heterocyclic library and found a potent inhibitor of patient-derived multidrug-resistant Acinetobacter baumannii strains that significantly reduced bacterial burden in an animal model of infection caused by carbapenem-resistant Acinetobacter baumannii (CRAB), listed as a priority 1 critical pathogen by the World Health Organization. Next, using advanced chemoproteomics platforms and activity-based protein profiling (ABPP), we identified and biochemically validated betaine aldehyde dehydrogenase (BetB), an enzyme that is involved in the metabolism and maintenance of osmolarity, as a potential target for this compound. Together, using a new class of heterocyclic iodonium salts, a potent CRAB inhibitor was identified, and our study lays the foundation for the identification of new druggable targets against this critical pathogen.

**IMPORTANCE** Discovery of novel antibiotics targeting multidrug-resistant (MDR) pathogens such as A. baumannii is an urgent, unmet medical need. Our work has highlighted the potential of this unique scaffold to annihilate MDR A. baumannii alone and in combination with amikacin both *in vitro* and in animals, that too without inducing resistance. Further in depth analysis identified central metabolism to be a putative target. Taken together, these experiments lay down the foundation for effective management of infections caused due to highly MDR pathogens.

## INTRODUCTION

The advent and clinical utilization of antibiotics this past century has improved life expectancies globally (1). However, the unrelenting rise in antimicrobial resistance (AMR) is rendering the current arsenal of antibiotics ineffective in treating infections caused by drug-resistant pathogens, thus leading to increased death and disability worldwide (1). It has been projected that, at this rate, treating even routine infections will be challenging in the coming decades. According to a recent report on bacterial AMR for 23 pathogens and 88 pathogen-drug combinations in 204 countries and territories, more than a million deaths were directly attributable to AMR in 2019 (1). The six leading pathogens responsible for this surge, Escherichia coli, Staphylococcus aureus, Klebsiella pneumoniae, Streptococcus pneumoniae, Acinetobacter baumannii, and Pseudomonas aeruginosa, are responsible for the majority of the drug-resistant infections and associated deaths globally (2).

In this cohort, A. baumannii, which causes a range of infections including pneumonia, meningitis, wound and surgical site infections, and urinary tract infections, is extremely challenging to treat, with outbreaks especially common among critically ill and immunocompromised populations, and is compounded by rampant multidrug resistance (MDR) with rapidly declining treatment options (2–4). Carbapenem-resistant A. baumannii (CRAB) is top on the critical priority list released by the World Health Organization (WHO), underscoring the urgency of developing novel therapeutics (5). Needless to say, AMR is a complex global public health problem and requires a multipronged strategy to address (6, 7).

**FIG 1:**
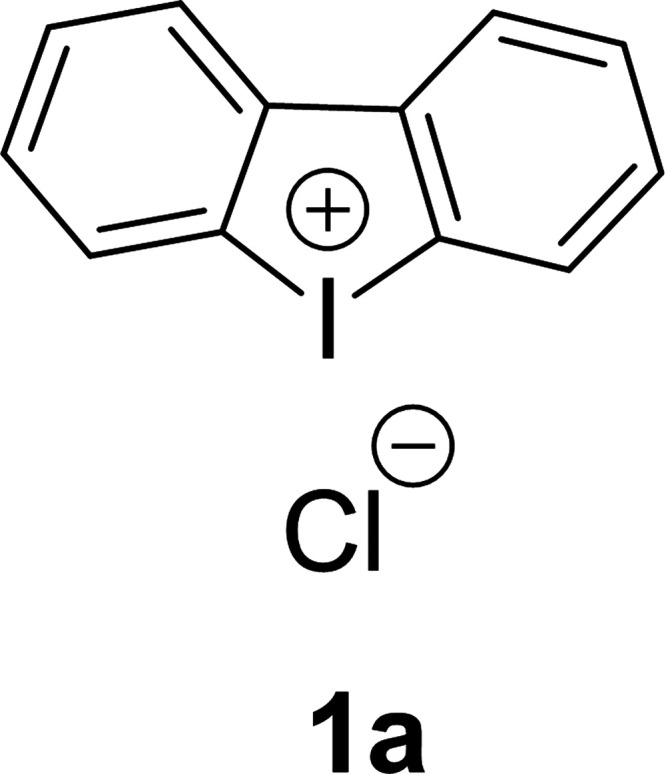
Diphenyl iodonium (dpI) chloride (1a).

Among the various approaches currently used to address AMR, the identification of new druggable targets and/or hot spots for MDR pathogens, screening for new chemotypes as drug candidates and adjuvants that sensitize pathogens to antibiotics, and repurposing of known drugs for new indications seem to be the most promising and popular (8–10). Iodonium salts such as diphenyleneiodonium (dpI) (Fig. 1) chloride are antiseptics against Gram-positive bacteria with moderate inhibitory activity against Gram-negative bacteria (GNB) such as A. baumannii (11–15). To the best of our knowledge, the biological targets and mechanisms of action of iodonium compounds in bacteria are not well characterized in the literature.

Although some studies show that iodonium compounds reversibly inhibit certain enzymes (16), the presence of a reactive iodonium functional group suggests that the compound is redox-active and can possibly generate a radical (17, 18) that can modify key catalytic residues of proteins or cofactors (19), which in turn can contribute to its inhibitory activity (20). The identity of such targets and the residues or functional groups involved in such modifications remain unclear. Delineating such targets will help reveal new vulnerabilities and possibly new druggable targets in MDR GNB pathogens. Herein, we synthesized and screened highly focused heterocyclic iodonium salt analogues and identified analogue 3a with exemplary antibacterial activity against A. baumannii. Moreover, 3a exhibited excellent inhibitory activity against a panel of patient-derived MDR A. baumannii strains and also substantially reduced the bacterial burden in a neutropenic murine thigh infection model. To understand the mechanism of action, we synthesized a biorthogonal iodonium probe and, using activity-based protein profiling (ABPP) and a liquid chromatography-mass spectrometry (LC-MS)-based chemoproteomics platform, demonstrated that iodonium compounds covalently modify cysteine residues of key proteins that rely on redox cofactors for their physiological functioning.

## RESULTS

### Structure-activity relationship study.

**FIG 2:**
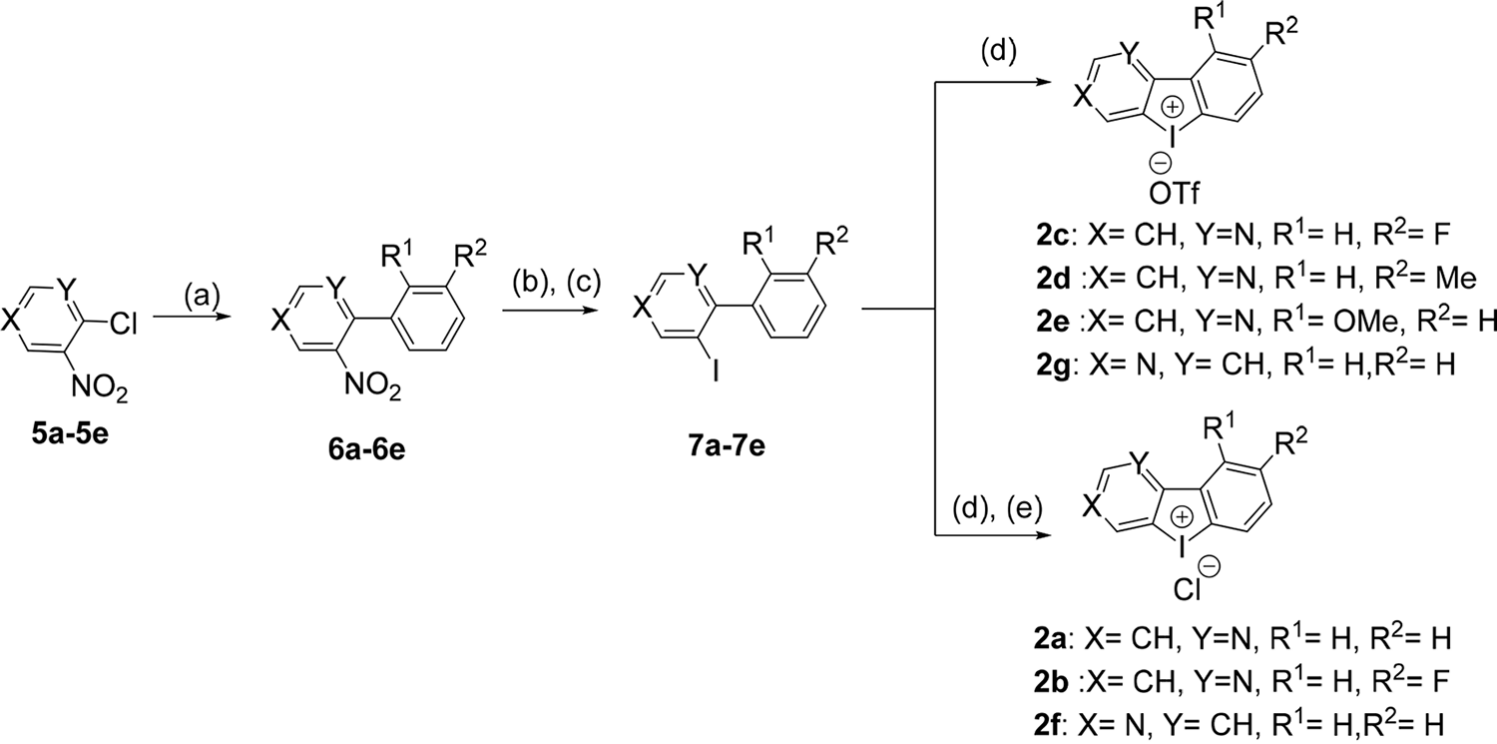
Synthesis of derivatives heterocyclic pyridine iodonium 2a to 2g. Reagents and conditions are as follows. (a) Aryl boronic acid, Pd(PPh_3_)_4_, K_3_PO_4_, EtOH, 85°C, 16 h. (b) Fe (powder), NH_4_Cl, EtOH:H_2_O (1:1). (c) NaNO_2_, KI, *p*-TsOH, MeCN, 0°C to RT, 12 h. (d) *m*-CPBA, triflic acid, dry DCM, 0°C. (e) Formic acid, aq. NaCl. DCM, dichloromethane.

Although 1a has good inhibitory activity against GNB, this compound has diminished potency against A. baumannii, and hence further structure-activity relationship (SAR) studies are much needed for this chemotype. We considered introducing a heterocyclic ring, which is a fairly common constituent of several drugs and drug-like privileged scaffolds, to iodonium compounds. To the best of our knowledge, a study on heterocyclic iodonium analogues has hitherto not been conducted. To this end, we synthesized a series of bi-aryl systems with one heterocyclic pyridine (hpI) (Fig. 2) using the following steps: Suzuki coupling was used to produce intermediates 6a to 6e from an aryl boronic acid and a chloro-nitro pyridine derivative in the presence of K_3_PO_4_ in ethyl alcohol (EtOH). Following this, these bi-aryl nitro intermediates were converted to bi-aryls amine using Fe powder and intermediates 7a to 7e were produced through the diazotization reaction in the presence of NaNO_2_, KI, and *p*-TsOH. The bi-aryl iodo intermediates were then oxidized in the presence of meta-chloroperoxybenzoic acid (*m*-CPBA) and triflic acid to produce heterocyclic pyridine iodonium triflate derivatives. An anion exchange approach with aq. NaCl and formic acid was used to produce heterocyclic pyridine iodonium chloride derivatives.

**FIG 3:**
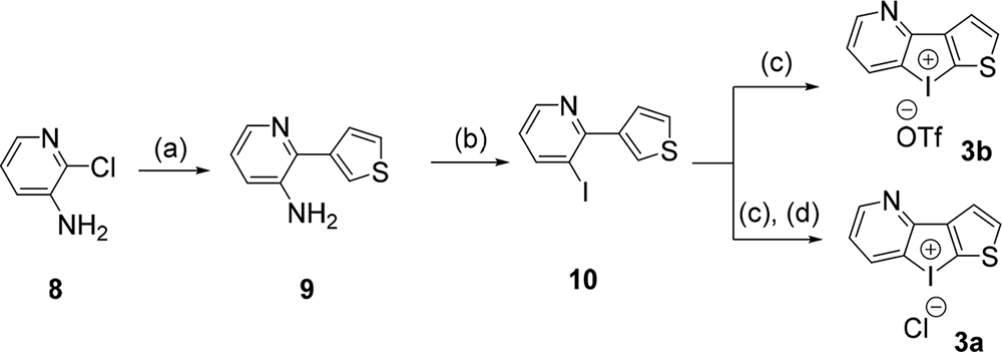
Synthesis of diheterocyclic iodonium derivatives 3a and 3b. Reagents and conditions. (a) 3-thienylboronic acid, Pd(PPh_3_)_4_, K_3_PO_4_, EtOH, 85°C, 16 h. (b) NaNO_2_, KI, *p*-TsOH, MeCN, 0°C to RT, 12 h. (c) *m*-CPBA, triflic acid, dry DCM, 0°C. (d) Formic acid, aq. NaCl.

Using pyridine as one of the heterocycles and thiophene as the second ring, a diheterocyclic iodonium derivative (dhI) (Fig. 3) was synthesized from 3-amino-2-chloropyridine. First, the Suzuki coupling produced intermediate 9, which was then diazotized to generate intermediate 10. This intermediate was then oxidized with *m*-CPBA and triflic acid to afford diheterocyclic iodonium triflate derivative. A diheterocyclic iodonium chloride derivative was obtained via an anion exchange method with aq. NaCl and formic acid.

Next, the MIC of the hpI and dhI series was determined against a panel of human bacterial pathogens of clinical relevance (Table 1). MIC data for various analogues against carbapenem-resistant A. baumannii BAA-1605 (CRAB) is listed in Table 1. The hpI series (2a to 2g) was found to have significantly improved activity against E. coli and A. baumannii compared with that of 1a. Conversely, dhI derivatives (3a and 3b) were most potent against A. baumannii among analogues tested (Table 1), and their activity is much better than polymyxin B (MIC, 0.5 μg/mL), which is the last line of treatment for serious GNB infections.

**TABLE 1:** Structure-activity study of iodonium compounds against ESKAP pathogens

Entry	Compound	MIC (μg/mL)				
		E. coli ATCC 25922	S. aureus ATCC 29213	K. pneumoniae BAA 1705	A. baumannii BAA-1605	P. aeruginosa ATCC 27853
1	1a	4	1	16	4	4
2	2a	0.25	0.5	2	0.125	0.25
3	2b	0.25	1	1	0.25	1
4	2c	0.125	0.25	2	0.125	0.5
5	2d	0.25	0.5	8	0.25	1
6	2e	0.5	1	2	8	4
7	2f	0.5	0.5	4	2	2
8	2g	0.5	0.5	2	2	2
9	3a	1	1	2	0.0625	1
10	3b	0.5	0.5	1	0.0625	0.5
11	Levo^*a*^	0.0156	0.125	64	4	0.5

a Levo, levofloxacin.

In the next step, the cytotoxicity for hpI and dhI analogues was carried out against Vero cells (see Table S1 in the supplemental material), and a selectivity index (SI = CC_50_/MIC) was determined and found to be favorable for further studies (SI >20) (CC_50_ is defined as the lowest concentration of compound which leads to a 50% reduction in cell viability).

**FIG 4:**
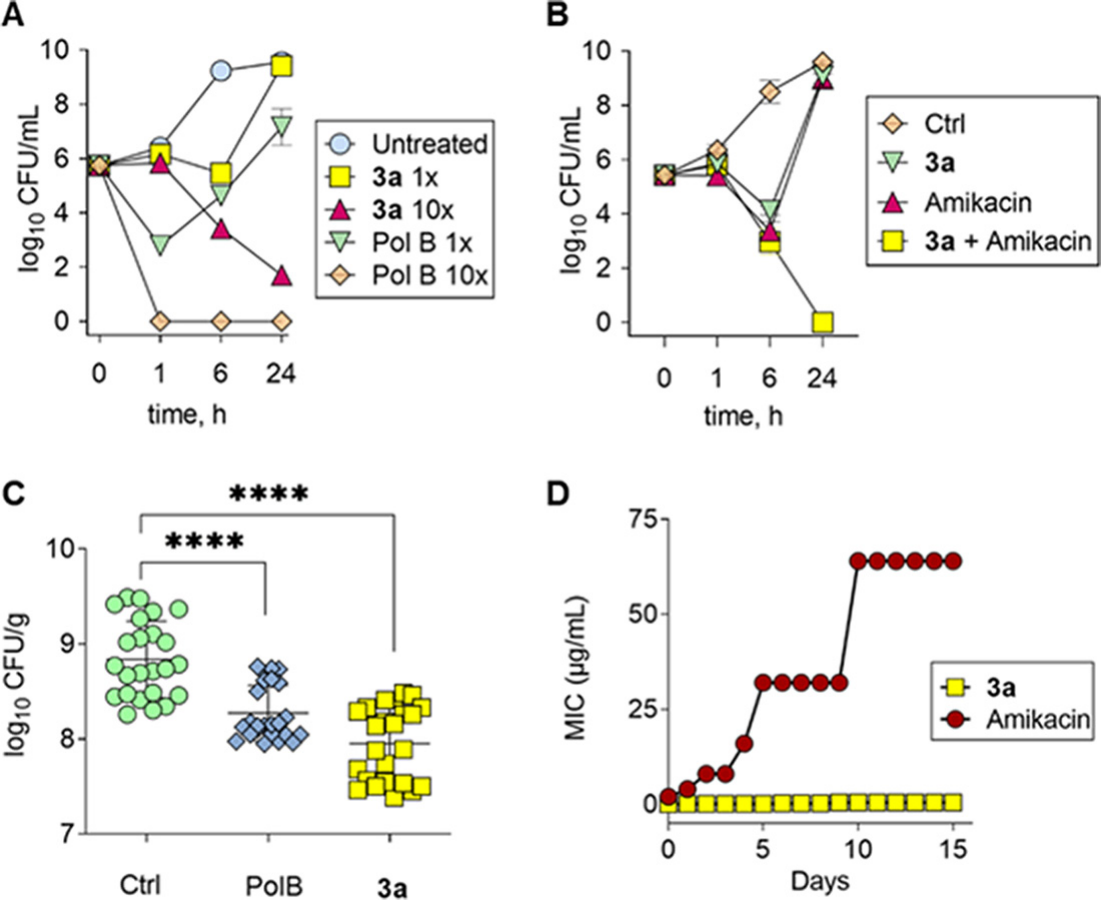
(A) Time-kill curves for A. baumannii BAA-1605 treated with 1× and 10× MIC of 3a (MIC, 0.0625 μg/mL) and polymyxin B (Pol B) (MIC, 0.5 μg/mL). Cell survival was plotted at the 24-h time point as log-change in CFU per milliliter (log_10_ CFU/mL) and curves show mean ± standard deviation of three independent experiments. (B) Synergy between 3a and amikacin was studied using a time-kill analysis where 3a (MIC, 0.0625 μg/mL) and amikacin (MIC, 8 μg/mL) were used at 1× MIC. When used alone, neither were as effective, but when used together, they were potent inhibitors of A. baumannii growth and eliminated all viable bacteria supporting synergy between two compounds. (C) *In vivo* efficacy of 3a in murine neutropenic thigh infection model. Pol B was used as a reference antibiotic while Ctrl is untreated mice. Statistical significance is calculated with respect to Ctrl (****, *P* < 0.0001). (D) Resistant mutant generation in A. baumannii BAA-1605 at subinhibitory concentrations of amikacin or 3a. The MIC of 3a remained consistent over 15 days, while a significant increase in MIC of amikacin was observed.

Further, the time-kill analysis experiments with dhI derivative 3a revealed that treatment with its 10× MIC reduced ~7 log_10_ CFU/mL bacteria in 24 h, whereas polymyxin B reduced ~9 log_10_ CFU/mL compared to that of untreated in 24 h with no regrowth. Thus, 3a exhibited concentration-dependent bactericidal activity, which is comparable to polymyxin B (Fig. 4A).

Also, 3a was found to be uniformly active against MDR patient-derived A. baumannii strains (see Table S2 in the supplemental material), whereas the comparators exhibited wide variation in activity with most strains expressing resistance to amikacin, tobramycin, meropenem, minocycline, and levofloxacin. The equipotent activity of 3a indicates its ability to bypass existing resistance mechanisms and act on a novel drug target.

To study if 3a synergized with major classes of clinically used antibiotics, fractional inhibitory concentrations (FIC) were determined with polymyxin B, amikacin, tobramycin, meropenem, minocycline, rifampicin, and levofloxacin (see Table S3 in the supplemental material). As seen, 3a synergized with amikacin (Table S3), and it was further validated by time-kill kinetics assay using 1× MIC of 3a and amikacin in combination against CRAB BAA-1605. The combination of amikacin and 3a completely eliminated all culture in 24 h compared to that of untreated and outperformed either drug alone (Fig. 4B; see also Fig. S1A and B in the supplemental material). A similar result was obtained in E. coli as well, which was also confirmed by time-kill analysis using 1× MIC of either drug (see Fig. S1C and D and Table S4 in the supplemental material).

Furthermore, the postantibiotic effect (PAE) of 3a was found to be 1 h at 10× MIC, which is equivalent to amikacin against A. baumannii (~1.5 h at 10× MIC) (see Table S5 in the supplemental material). Administration of a single 10-mg/kg intraperitoneal (i.p.) dose of 3a in mice resulted in no mortality over a 5-day observation period, indicating the maximum tolerable dose (MTD) to be ≥10 mg/kg (see Table S6 in the supplemental material). Next, to determine if the *in vitro* potency of 3a can be translated *in vivo*, a neutropenic thigh infection model was investigated. Analogue 3a at 1 mg/kg exhibited excellent efficacy against A. baumannii BAA-1605 by reducing ~0.9 log_10_ CFU/g bacteria in a neutropenic thigh infection model (Fig. 4C). In contrast, clinically used antibiotic polymyxin B, as the comparator in this study, reduced ~0.56 log_10_ CFU/g bacteria at a 5-mg/kg dose.

Next, the ability of A. baumannii to develop resistance to the lead compound 3a was studied through a serial drug exposure resistance model. A. baumannii BAA-1605 continued to be susceptible to 3a over a period of 15 days (Fig. 4D). However, it developed resistance to amikacin in 5 days with a considerable rise in MIC under similar conditions.

Taken together, 3a exhibits equipotent activity against multiple clinical MDR strains, possesses concentration-dependent bactericidal activity, and does not induce resistance in CRAB BAA-1605 with excellent *in vivo* efficacy.

### Synthesis and validation of an iodonium probe (P1).

**FIG 5:**
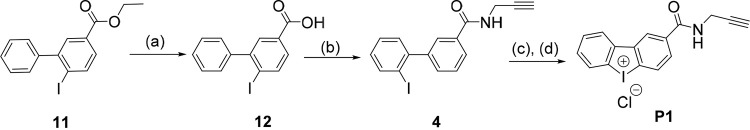
Synthesis of an iodonium probe P1. Reagents and conditions. (a) KOH, EtOH:H_2_O (1:1) 80°C, 4 h. (b) Propargyl amine, EDC·HCl, DMAP, RT, 36 h. (c) *m*-CPBA, triflic acid, dry DCM, 0°C. (d) Formic acid, aq. NaCl. DMAP, 4-dimethylaminopyridine.

The probe P1 with a biorthogonal alkyne handle was synthesized in three steps from ethyl 6-iodo-[1,1′-biphenyl]-3-carboxylate (21), an ethyl ester of biphenyl iodide (Fig. 5). First, the hydrolysis of ester 11 afforded an acid (12). This compound was reacted with propargyl amine to produce the iodoaryl compound 4. The final step was the oxidation of the aryl iodide by m-CPBA and triflic acid and subsequent treatment of this salt with NaCl in formic acid to afford the iodonium-based probe P1. Having made the probe P1 and before embarking on further studies, we first wanted to validate if P1 inhibited E. coli growth and, toward this, we monitored bacterial growth in the presence of P1 and 1a. From this experiment, we found that P1 at 16 μg/mL concentration inhibited E. coli growth over 5 h as determined by optical density at 600 nm (OD_600_) at concentrations similar to that effect was seen with the parent compound 1a and levofloxacin, which was used as a positive control (see Fig. S2 in the supplemental material).

### Chemoproteomics with P1 to identify targets of 1a in E. coli.

**FIG 6:**
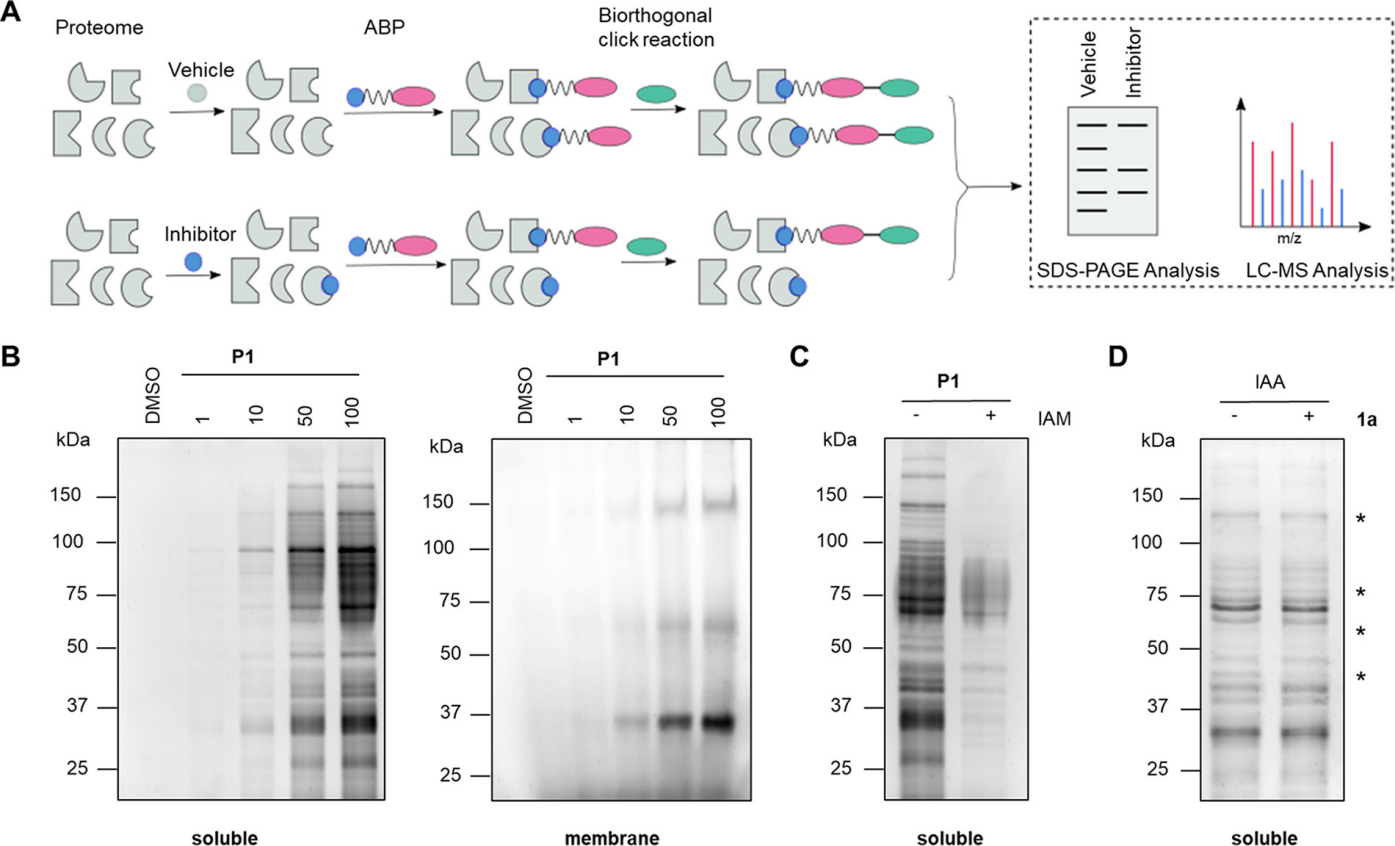
(A) Schematic representation of activity-based protein profiling for target identification and visualization using activity-based probe (ABP). (B) Dose-dependent protein labeling by P1 (1 to 100 μM) in soluble and membrane proteomic fractions of E. coli ATCC 25922. (C) Proteome profiling of E. coli soluble protein by P1; the lysate was pretreated with DMSO or iodoacetamide (IAM, 10 mM), a thiol-modifying electrophile followed by treatment with P1 (100 μM). (D) Proteome profiling by IAA (100 μM) of E. coli soluble protein pretreated with DMSO or 1a (250 μM). The asterisks (*) denote proteins for which labeling by IAA is diminished in the presence of 1a (see Fig. S3 in the supplemental material for Coomassie-stained gel image).

E. coli cells were lysed and fractionated into soluble and membrane proteomes using protocols reported earlier (22, 23). To determine if P1 binds to and/or covalently modifies proteins, we first decided to perform gel-based experiments, where the proteomic fractions were treated with increasing concentrations of P1 (Fig. 6A). Indeed, from this experiment, we find a dose-dependent labeling of the E. coli soluble and membrane proteomes by P1 (Fig. 6A). However, the labeling of membrane proteome was less prominent than soluble proteome, and hence, the latter was used in this study. Considering that thiols are among the best biological nucleophiles, we hypothesized that P1 might be forming a covalent adduct with proteins through cysteine residue. To test this premise, we pretreated the E. coli soluble proteome with vehicle (dimethyl sulfoxide [DMSO]) or the generic cysteine binding electrophile, iodoacetamide (IAM) (10 mM, 1 h) (24), and then chased with P1 (100 μM, 1h).

The pretreatment with IAM substantially reduced the ability of P1 to label the soluble proteome of E. coli (Fig. 6B). Conversely, we also pretreated the E. coli soluble proteome with either vehicle (DMSO) or 1a (250 μM, 1 h) and then chased with an alkynated version of IAM (iodoacetamide-alkyne [IAA]) (100 μM, 1 h) (25). Consistent with this hypothesis, we found a few protein bands disappear from the gel upon pretreatment with 1a (Fig. 6C), further confirming that both 1a and its corresponding probe P1 are modifying protein cysteine thiols.

**FIG 7:**
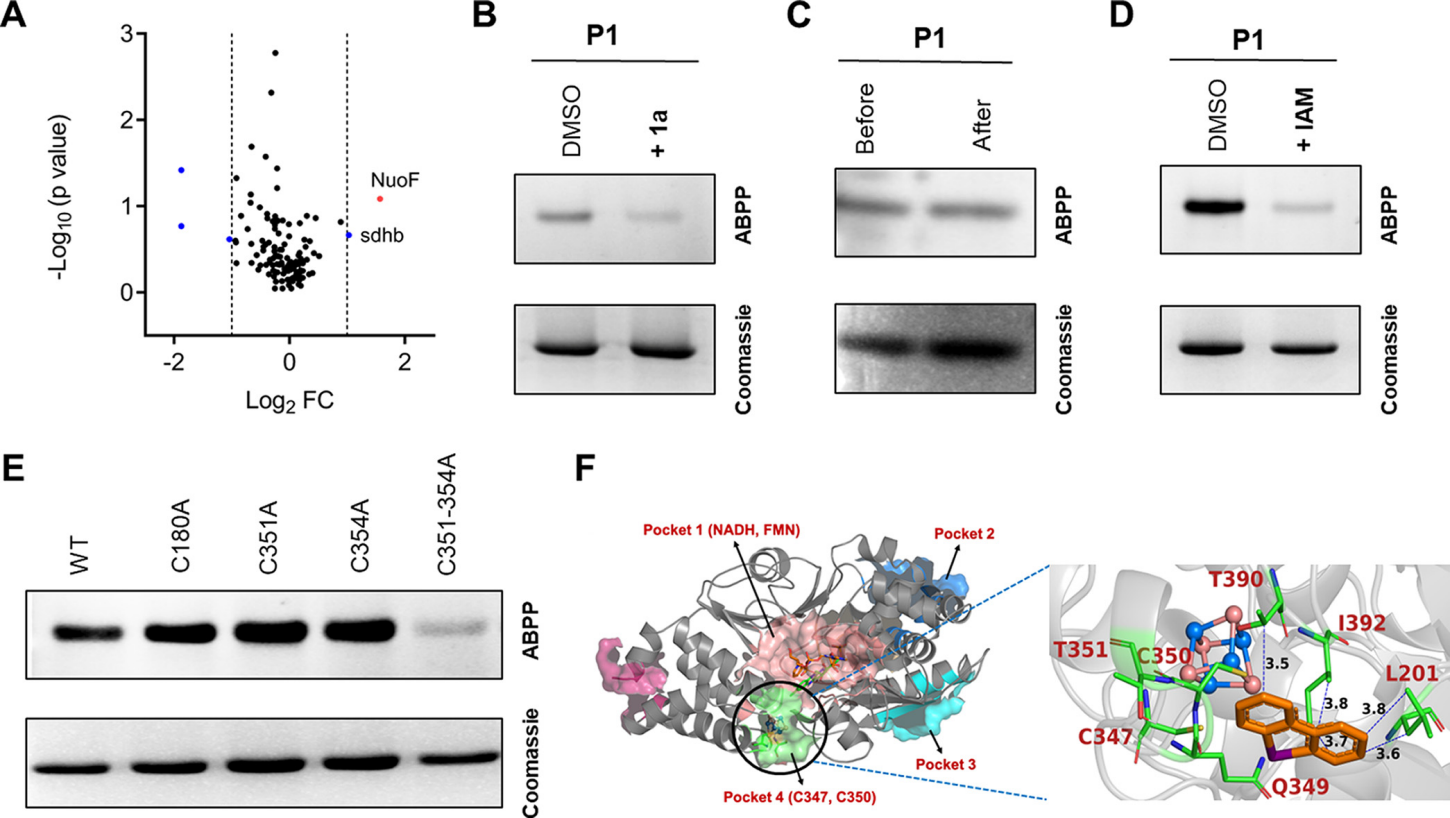
(A) Volcano plot of chemoproteomics for E. coli using 1a and P1, depicting protein data *P* values versus fold change (FC). Blue points represent data points with a fold change of > ±2. (B) E. coli NuOF protein (5 μM) treated with DMSO or 1a (250 μM, 1 h) followed by treatment with P1 (5 μM) shows inhibition of labeling by P1 of E. coli NuOF by 1a. (C) E. coli NuoF (5 μM) treated with P1 (25 μM). This mixture was separated into two parts. One was desalted using dialysis membrane (MWCO, 6 to 8 kDa). Visualization was carried out on samples before and after dialysis. (D) E. coli NuOF (5 μM) was treated with IAM (0.5 mM, 1 h) followed by treatment with either DMSO or P1 (100 μM, 1 h) and indicates the modification of cysteine residues of protein. (E) Labeling of NuOF (5 μM) with P1 (50 μM) compared to its alanine point variants (C180A, C351A, C354A, C351-354A), suggesting C351 and C354 as the sites for modification by P1. (F) Labeling of NuOF (5 μM) with P1 (50 μM) compared to its alanine point variants (C180A, C351A, C354A, C351-354A) suggesting C351 and C354 as the sites for modification by P1. (I) Ligand-binding pockets in NuOF protein from *A. aeolicus* (PDB ID number 6Q9K) for 1a using the Computed Atlas of Surface Topography of proteins (CASTp 3.0) web server. The protein surface is illustrated as a gray-colored cartoon, bound ligands (NADH and FMN) as sticks, Fe-S cluster as ball and stick representation, and the identified pockets as transparent surfaces. The primary pocket is shown in salmon red, the secondary pocket in marine blue, the tertiary pocket in cyan, and the last pocket containing C347 and C350 in green. (Inset) *In silico* docking analysis into the catalytic site (pocket 4) of NuOF (PDB ID number 6Q9K) with 1a shows a favorable binding conformation. The protein is depicted in ribbon style, Fe-S cluster is represented in ball and stick model, while the docked ligand and the residues (indicated by one-letter code) are shown in stick representation.

Finally, the targets of 1a in E. coli were identified using previously reported liquid chromatography-tandem mass spectrometry (LC-MS/MS) proteomics experiment (22, 26). Among 125 identified proteins (see the supplemental material), three proteins, namely, NADH-quinone oxidoreductase subunit F (NuOF) and succinate dehydrogenase iron-sulfur subunit, were shortlisted as putative targets based on the selection threshold *a priori* set by us (fold change, >2; *P* value, <0.09) (Fig. 7A).

### Biochemical validation of the targets of 1a in E. coli.

Not surprisingly, and consistent with literature, the putative protein targets of 1a were in fact redox proteins (27–30), among which, the most inhibited protein was the NuOF, which is known to part of the respiratory complex in E. coli (31). We cloned and recombinantly purified NuOF from E. coli using affinity chromatography and performed ABPP experiments to validate the interaction of 1a with NuOF. First, we pretreated NuOF with vehicle (DMSO) or 1a (250 μM, 1 h) and then chased with P1 (5 μM, 1 h). This experiment suggests that 1a has effectively blocked the labeling of NuOF by P1 and, thus, showed that NuOF was in fact a target of 1a (Fig. 7B). Second, to determine if the interaction of 1a with NuOF was indeed covalent, we treated NuOF with P1 (25 μM, 1 h), and a part of this protein mixture was dialyzed using a dialysis membrane (molecular weight cutoff [MWCO], 6 to 8 kDa). A click reaction was performed with both dialyzed and nondialyzed protein mixture and visualized for in-gel fluorescence after SDS-PAGE. This experiment revealed that the extent of labeling by P1 with or without dialysis was almost identical, and hence, it appears that P1 (and in turn 1a) forms an irreversible, covalent adduct with NuOF (Fig. 7C).

Third, to confirm if the interaction of P1 (or 1a) with NuOF was covalent through a cysteine residue, we pretreated NuOF with vehicle (DMSO) or IAM (0.5 mM, 1 h) and then chased with P1 (100 μM, 1 h). We found that treatment with IAM almost completely ablated the ability of P1 to label NuOF (Fig. 7D), suggesting that the interaction of P1 or 1a with NuOF (and other protein targets) is through the formation of a covalent protein adduct with a cysteine residue. A bioinformatics analysis suggested that E. coli NuOF has five conserved cysteine residues that, in principle, can be modified by P1. Of these, Cys 180 is involved in binding of flavin mononucleotide (FMN), and Cys 351, Cys 354, Cys 357, and Cys 398 are part of an iron-sulfur cluster (31). Hence, we decided to make single-point alanine mutants for protein cysteine residues as follows: Cys 180, Cys 351, and Cys 354 (see Fig. S4B to D in the supplemental material). Next, we treated all of these alanine variants with P1. (Fig. 7D; see also Fig. S5A to C in the supplemental material). Interestingly, we found no significant change in the labeling profile with any of the single alanine variants. Hence, we decided to make a double alanine mutant for Cys 351 and Cys 354 residues (see Fig. S4E). When the labeling experiment was conducted, we found that the double mutant, C351A C354A, showed a significant decrease in labeling with P1 (Fig. 7D; see also Fig. S5D). Unfortunately, the structure of E. coli NuOF is not publicly available in PDB. However, a high-resolution three-dimensional structure of NuOF from Aquifex aeolicus (32) that shares 43% sequence identity with E. coli NuOF and has all of the aforementioned conserved cysteine residues as part of the structure is available, and we used this structure for further computational analysis. We performed a molecular docking study on possible 1a binding sites in NuOF from *A. aeolicus* and identified four putative binding sites (Fig. 6F). Notably, and in sync with our experimental studies, we found that binding pocket 4 showed the lowest energy conformation and was in closest proximity to two cysteine residues Cys 347 and Cys 350, which correspond to Cys 351 and Cys 354 in E. coli NuOF. Together, our studies showed with sufficient experimental evidence, that Cys 351 and Cys 354 are covalent targets for 1a and/or P1 for E. coli NuOF. In addition, the predicted structure by AlphaFold algorithm was next used to study the binding of these analogues to NuOF (see Fig. S7 in the supplemental material). We found these binding modes analogous to the study with NuOF from *A. aeolicus* (see Fig. S6 in the supplemental material).

### Identification of targets of 3a in A. baumannii.

**FIG 8:**
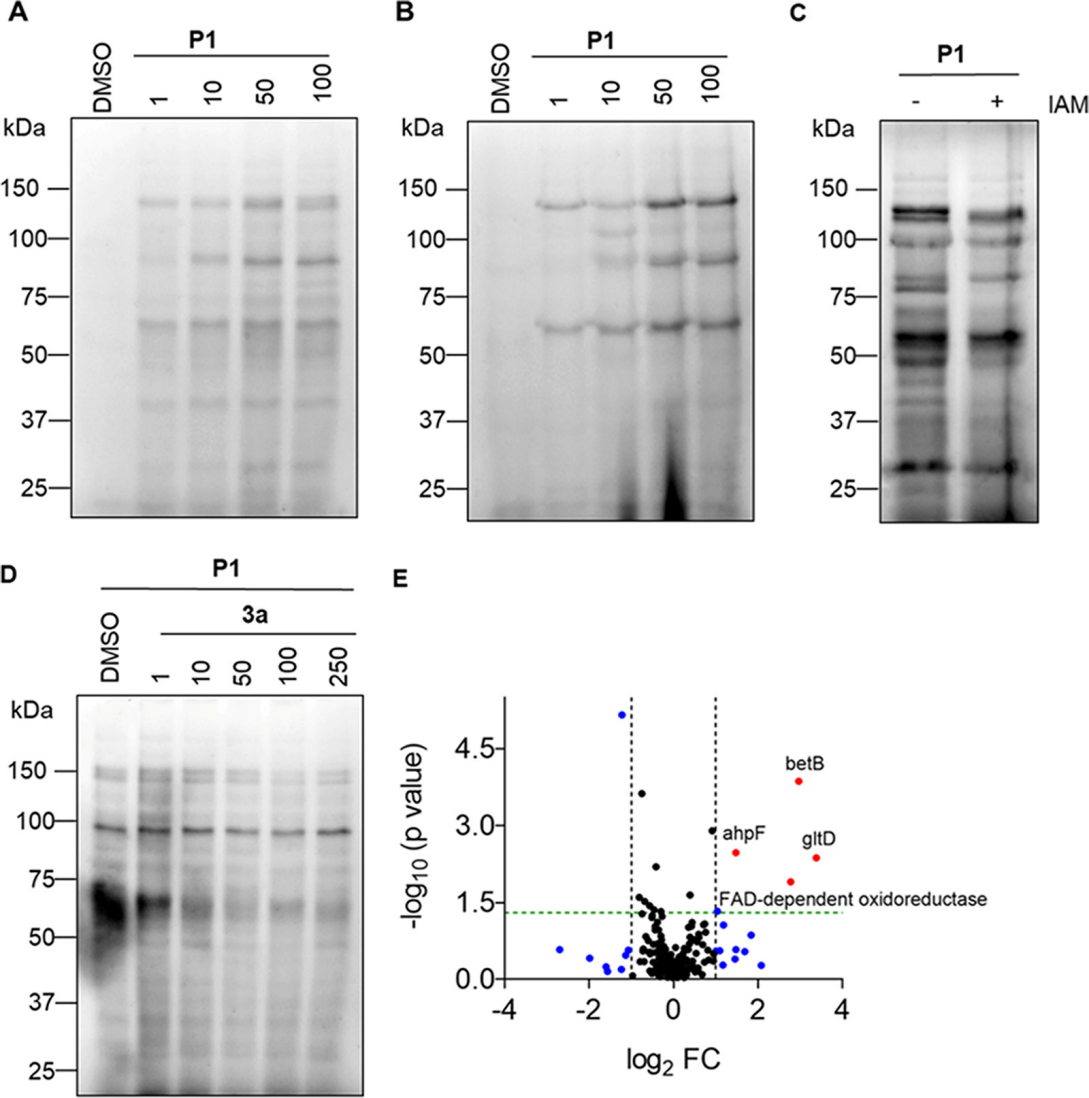
Dose-dependent protein labeling by P1 (1 to 100 μM) in soluble (A) membrane proteomic fraction (B) of A. baumannii ATCC 17978. (C) Proteome profiling of A. baumannii soluble protein by P1; the lysate was pretreated with DMSO or iodoacetamide (IAM, 10 mM), a thiol modifying electrophile followed by treatment with P1 (100 μM). (D) Proteome profiling by P1 (100 μM) of A. baumannii soluble protein pretreated with DMSO or 3a (250 μM), (see Fig. S8 in the supplemental material for Coomassie-stained gel image). (E) Volcano plot of chemoproteomics for A. baumannii using 3a and P1, depicting protein data *P* values versus fold change (FC). Blue points represent data points with a fold change of > ±2, and red points above the green horizontal line represent data points with *P* value < 0.05 and a fold change > 2.

Finally, to identify the targets of 3a in A. baumannii, we prepared soluble and membrane proteomic fractions by lysing A. baumannii cells using established protocols (22, 26). Next, as described before for E. coli, gel- and LC-MS/MS-based chemoproteomics experiments were carried out using lead compound 3a (250 μM, 1 h) as the compound of interest and P1 (100 μM, 1 h) as a probe to chase with. From the ABPP experiments, we found a dose-dependent labeling with P1 in both the soluble (Fig. 8A) and membrane (Fig. 8B) proteomes of A. baumannii. Similar to E. coli, we found a decrease in labeling by P1 (100 μM, 1 h) when the proteome was pretreated with IAM (100 μM, 1 h), a thiol-modifying agent (Fig. 8C). The soluble fraction of A. baumannii lysate was next treated with 3a (1 to 250 μM, 1 h) followed by treatment with P1 (100 μM, 1 h). A significant decrease in proteome labeling by P1 in the presence of 3a supports the use of P1 as a probe for further studies (Fig. 8D). Next, from the LC-MS/MS based chemoproteomics experiment, we identified a total of 219 identified targets of which, after applying the appropriate filters, four proteins were shortlisted as probable targets of 3a (Fig. 8E; see also Table S11 in the supplemental material). Like E. coli, not surprisingly, the identified candidate proteins in A. baumannii were also redox proteins that used NADH as a cofactor. Two particular proteins that were identified among the top hits are glutamate synthase subunit D (GltD) (33) and betaine aldehyde dehydrogenase (BetB) (34), which have important roles in metabolism and growth.

### Biochemical validation of the targets of 3a in A. baumannii.

**FIG 9:**
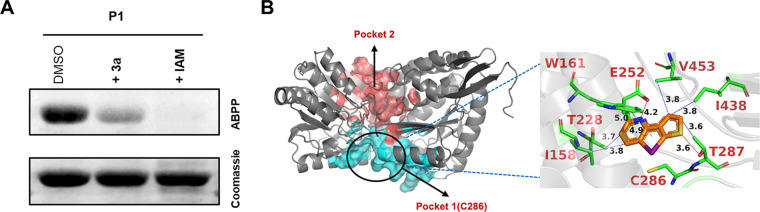
(A) A. baumannii BetB protein (5 μM) treated with DMSO or 3a (250 μM, 1 h) or IAM (0.5 mM, 1 h) followed by treatment with P1 (10 μM) shows inhibition of labeling by P1. (B) Ligand-binding pockets in alpha-fold model of BetB protein from A. baumannii AB0057 (B7I896 (BETB_ACIB5) for 3a using the Computed Atlas of Surface Topography of proteins (CASTp 3.0) web server with a probe radius of 1.4 Å. The protein is illustrated as a gray-colored cartoon, and the identified pockets are illustrated as transparent surfaces. The primary pocket containing cysteine residue (C286) is shown in salmon red and the last pocket in cyan. (Inset) *In silico* docking analysis into the catalytic site (pocket 1) of alpha-fold predicted model of BetB protein from A. baumannii with 3a shows a favorable binding conformation. The protein is depicted in ribbon style, while the docked ligand and the residues (indicated by one-letter code) are shown in stick representation. The blue and red dotted lines indicate hydrophobic and hydrogen bonding interactions, respectively, and the lengths are indicated.

The enzyme BetB was cloned and purified using standard biochemical techniques (Fig. S4F). A competition experiment was next carried out with P1, 3a, and IAM (Fig. 9A). The results of this experiment are consistent with a covalent modification of the protein by the P1, which is abrogated by pretreatment with 3a or IAM. *In silico* docking analysis into the catalytic site (pocket 1) of alpha-fold predicted model of BetB protein from A. baumannii with 3a shows a favorable binding conformation (Fig. 9B).

### Biochemical mechanism of activation and covalent modification.

**FIG 10:**
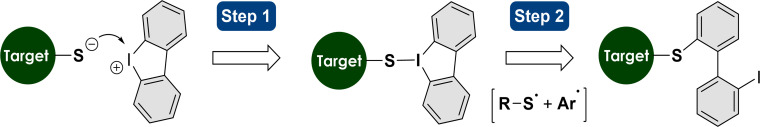
Proposed mechanism for the formation of a covalent adduct with dpI. Step 1 is the attack of the cysteine residue of the target protein (NuOF, for example) on the iodonium group leads to the formation of an adduct, which undergoes reductive elimination in step 2 to produce two radical species, i.e., the thiyl radical (R-S^•^) and aryl radical (Ar^•^), which then combine to give the covalent adduct.

The reaction of iodonium salts with nucleophiles in organic media is well documented (19, 35, 36). Based on these reports, we proposed a mechanism of covalent modification with two major steps (Fig. 10). First, addition of the cysteine-containing protein (for example, NuOF) to dpI produces a neutral adduct (step 1), which can then undergo a reductive elimination to produce a thiyl radical (R-S^•^) and an aryl radical (Ar^•^); recombination of these radicals (step 2) produces the covalent adduct (Fig. 10).

Hence, the adduct formation is expected to depend on the electrophilic nature of the iodonium functional group, C–I bond strength, as well as radical stability. Orbital calculations show that the LUMO of dpI was 26.16 meV while the lowest unoccupied molecular orbital (LUMO) for hpI and dhI were 13.97 meV and 24.85 meV, respectively, suggesting the order of reactivity with nucleophile as dpI > dhI > hpI (see also Fig. S6 in the supplemental material). In order to understand the propensity of iodonium salts to undergo reduction, cyclic voltammetry analysis on dpI was conducted. As expected, an irreversible reduction process and a potential of −0.853 V was obtained (see Fig. S9 and Table S12 in the supplemental material), while no significant oxidation step was observed. A similar column volume (CV) profile for hpI was obtained with a slightly lower reduction potential of −0.711 V, suggesting a higher propensity for this compound to undergo reduction when compared with that of dpI.

## DISCUSSION

The efficacy of dpI and derivatives has been previously demonstrated in Gram-positive bacteria Staphylococcus aureus and Streptococcus pyogenes, Gram-negative pathogen Helicobacter pylori, as well as Mycobacterium tuberculosis, including MDR strains of these bacteria (11–14). Most derivatives were found to have moderate inhibitory potency against E. coli. Nguyen and coworkers showed 5-chlorodiphenyleneiodonium triflate as a potent inhibitor of MDR ESKAPE (Enterococcus faecium, Staphylococcus aureus, Klebsiella pneumoniae, Acinetobacter baumannii, Pseudomonas aeruginosa, and Enterobacter species) pathogens (15). This compound was, however, found to be quite toxic to mammalian cells. Our studies on heterocyclic derivatives, which to the best of our knowledge have not been previously reported, show a significant improvement in the selectivity index. We demonstrate the applicability and translational potential for the lead compound dhI through a series of complementary *in vitro* and *in vivo* experiments. Targets for dpI in bacteria have not been systematically investigated, and little is known about the targets in Gram-negative bacteria. The iodonium series of compounds have been investigated in mammalian systems, and dpI inhibits NADPH oxidase (20), nitric oxide synthase (NOS) (28), monoamine oxidase (MAO) (16), and NADH-ubiquinone oxidoreductase (27) as well as other flavin-containing enzymes (37). Although some studies show that the mode of binding of certain enzymes is irreversible and covalent, the amino acid residue in question is not clear. Other studies on dpI show that this compound forms a covalent bond with flavin to form an adduct but is not a specific flavin binder (19, 38). In cellular model systems, iodonium salts significantly inhibit in a dose-dependent way both the pentose phosphate pathway and the tricarboxylic acid cycle and inhibit mitochondrial function (29). Together, these studies primarily carried out in mammalian cells and purified enzymes show that dpI can bind to certain proteins reversibly and bind irreversibly with certain other proteins. It is likely not a specific NADPH oxidase inhibitor, and this compound may interact with several other proteins whose identities remain to be determined. Here, using a new alkyne-containing probe and advanced chemoproteomics platforms, we demonstrated that in E. coli, the possible targets for dpI are respiratory proteins, all dependent on redox cofactors such as NADH. This observation is consistent with previous data with mammalian cells where dpI affects respiration in mammalian cells (30). We conclusively showed that the modification by the probe is covalent and through a systematic site-directed mutagenesis study, identified key cysteine residues that are targets for modification by dpI. The preference for covalent modification of certain cysteine residues over others is important since previous studies on other cysteine-containing proteins such as MAO show that the binding was reversible. Whereas, with other enzymes such as NOS, it was shown that the modification is covalent. NOS contains cysteine residues (39), and these may contribute to covalent modification of NOS by dpI. Hence, dpI can act both through noncovalent and covalent binding depending on the protein. This observation is useful to address the differences in outcomes that are observed in previous studies.

In A. baumannii, the major proteins that were identified as the targets were also those dependent on redox cofactors. Glutamate synthetase, which is key to contributing to the amino acid pool, is among the top proteins modified by iodonium salts and contains four iron-sulfur clusters (33). The other target protein identified is BetB, which belongs to the aldehyde dehydrogenase family and contains one key cysteine residue that is crucial for catalysis (40). This protein is involved in the biosynthesis of glycine betaine, where it irreversibly oxidizes betaine aldehyde to glycine betaine. The sulfhydryl group of cysteine attacks the aldehyde of betaine to produce a tetrahedral intermediate, which then transfers hydride to NAD^+^ to produce NADH to give a thioester. Water-mediated ester hydrolysis gives a carboxylic acid and restores the catalytic cysteine residue (41). Hence, covalent modification of the cysteine is likely to impact catalysis by this enzyme (42). Furthermore, glycine betaine is an osmoprotectant, which protects the bacterial cells against environmental stress or drought conditions (34, 43). Inhibition of BetB would also block the oxidation of betaine aldehyde, which itself is toxic to cells. GltD is involved in the l-glutamate biosynthesis pathway along with the amino acid synthesis pathway. Hence, 3a can stress bacteria through different mechanisms and may hence contribute to weakening of the pathogen’s defense mechanisms.

Due to polar groups in their structure, heterocyclic compounds when compared with their aryl counterparts will have differences in binding to the protein target, cell permeability, radical stabilizing capability, as well as reactivity with cysteine. Together, these factors contribute to differences in potency among the dpI, hpI, and dhI derivatives. Docking analysis of NuOF with various analogues showed no major difference in binding (see Fig. S6 and Table S10 in the supplemental material). However, further systematic studies on the other parameters through an expansion of this library will help delineate these effects and progress toward identification of more potent analogues. Much like other iodonium salts, the targets for dhI may vary depending on the cells in question and may have pleiotropic effects on cells. Nevertheless, the potent activity against drug-resistant strains, in particular, CRAB strains, and the promising data in animal studies underscore the therapeutic potential for this class of compounds. The lack of observable resistance being developed and the high potency of inhibiting patient-derived multidrug-resistant strains is highly encouraging from a translational standpoint. The lead compound 3a was also found to have potent inhibitory activity against mycobacteria including nontubercular mycobacteria, which are increasingly difficult infections to treat (see Table S7 in the supplemental material). Lastly, we find that 3a synergizes with aminoglycosides (amikacin) among all other classes of antibiotics tested (see Fig. S1A in the supplemental material). Aminoglycosides inhibit protein synthesis and synergy supports the favorable overlap of targets and the possible use of 3a as an adjuvant with amikacin. Glutamate is a key amino acid whose synthesis would be affected due to modification of GltD and would likely affect the amino acid pool that is required for protein synthesis. Together, these effects would increase the vulnerability of A. baumannii toward aminoglycosides, which are among the frontline antibiotics in the treatment of such infections.

In conclusion, our study with the new alkyne-containing iodonium probe P1 demonstrates that chemoproteomics is a powerful approach to identify covalently modified targets in bacteria. The major proteins that were modified covalently by iodonium salts all have redox cofactors. Our study shows that two cysteine residues of several possible cysteines were selectively modified in E. coli NuOF by 1a. In A. baumannii, the major targets of 3a identified were BetB and GltD, which are cysteine-containing proteins involved in key cellular metabolism processes. Given the wide use of dpI in several mammalian cellular and animal model systems, from the translational standpoint, a major roadblock for further development has been the lack of selectivity and diminished activity against Gram-negative bacteria including A. baumannii. Our study shows the potential for using a focused library of molecules that can help improve potency of a primarily Gram-positive inhibitor to a Gram-negative antibacterial compound. Finally, the synergy with amikacin provides another avenue for further development of iodonium compounds as an adjuvant that can exacerbate the pressure on aminoglycosides as frontline antibiotics in the treatment of MDR A. baumannii infections.

## MATERIALS AND METHODS

### MIC determination of P1 against E. coli.

Briefly, E. coli ATCC 25922 cultures were inoculated in Luria-Bertani broth medium and optical density (OD) of the cultures was measured at the 600 nm wavelength, followed by dilution to achieve ~10^5^ CFU/mL. The probe P1 was tested ranging from 2 to 16 mg/L in 2-fold serial diluted fashion with 2.5 μL of each concentration added to each well of a 96-well round bottom microtiter plate. Later, 97.5 μL of bacterial suspension was added to each well containing the test compound along with appropriate controls. The plates were incubated at 37°C for 18 to 24 h following which the growth was enumerated and MIC was identified. The MIC is defined as the lowest compound concentration where there is no visible growth. For each compound, MIC determinations were carried independently 3 times using duplicate samples.

### Protocols for ABPP experiments. (i) Preparation of proteomic fractions.

E. coli ATCC 2592 was cultured in Luria-Bertani broth medium at 37°C overnight. The cultured bacteria were resuspended in 1× phosphate-buffered saline (PBS), lysed using a probe sonicator, followed by centrifugation at 100,000 × *g* at 4°C for 1 h to separate soluble and membrane protein fractions. Soluble fractions were then separated from the membrane pellets. The membrane pellets were washed, followed by resuspension in 1× PBS. The protein concentration was measured by conducting either Bradford assay (Bio-Rad) or bicinchoninic acid (BCA) assay using bovine serum albumin (BSA) as a standard, and lysates were finally diluted to 1 mg/mL protein concentration with 1× PBS, which was used for all further work (22).

### (ii) Chase experiment.

One hundred microliters of 1 mg/mL protein for both membrane and soluble fraction was taken in a 1.5-mL microcentrifuge tube. One microliter of a 100× stock solution of the compound 1a in DMSO was added and incubated for 60 min at 37°C at 700 rpm. To the above solution, IAA or P1 probe (1 μL of a 100× stock solution in DMSO) was added and incubated for 60 min at 37°C at 700 rpm. A “click” mixture (11 μL) consisting of a 6 μL tris((1-benzyl-4-triazolyl)methyl)amine (TBTA) (1.7 mM in 4:1 DMSO-*^t^*BuOH), 2 μL CuSO_4_·5H_2_O (50 mM in water), 2 μL Tris(2-carboxyethyl)phosphine hydrochloride (TCEP) (50 mM in DPBS [Dulbecco’s PBS], 1×), and 1 μL rhodamine azide (Alexa Fluor 488, 10 mM in DMSO) was added to every tube and incubated for 60 min at 25°C at 700 rpm. The loading dye (4×, 40 μL) was added to every tube prior to loading the samples onto the gel.

### (iii) Mass spectrometry-based chemoproteomics.

E. coli or A. baumannii cells were lysed in 1× PBS by sonication and fractionated into membrane and soluble fractions by ultracentrifugation (100,000 × *g*, 4°C, 1 h). The soluble fraction (1 mL) was incubated with 1a, 3a, or vehicle (DMSO) at 250 μM final concentration for 1 h at 37°C at 700 rpm. Each group contained three biological replicates. The compound- or vehicle-treated soluble proteomes were chased with P1 (100 μM, 1 h at 37°C, 700 rpm). The click reaction was done with biotin-azide. Postbiotinylation, the proteomes were denatured and precipitated using methanol/chloroform (4:1) at 4°C and resuspended in urea (0.5 mL 6 M in DPBS) by sonication. Reduction and alkylation were carried out with TCEP (10 mM) for 30 min at 37°C with constant shaking and IAM (50 mM) for 30 min at room temperature (25°C) in the dark, respectively. The biotinylated proteins were enriched using avidin-agarose beads (100 μL; Sigma-Aldrich) by shaking at room temperature for 1.5 h in DPBS containing 0.2% (wt/vol) SDS in a final volume of 6 mL. The beads were pelleted by centrifugation (1,000 × *g*, 25°C, 5 min) and sequentially washed using 10 mL of 0.2% (wt/vol) SDS in DPBS (3 times), 10 mL of DPBS (3 times), and 10 mL of deionized water (3 times). The beads were transferred to a Protein LoBind 1.5-mL microcentrifuge tube. On-bead protein digestion was performed using sequence grade trypsin (1.5 μg; Promega) in 200 μL of urea (2 M in 100 mM triethylammonium bicarbonate buffer [TEAB]) at 37°C for 14 h at 180 rpm. The reaction was quenched by adding trifluoroacetic acid (TFA) to a final concentration of 1% (vol/vol). Peptides were cleaned on C_18_ stage tips and subjected to LC-MS/MS analysis on an Agilent 6540 accurate-mass quadrupole time-of-flight (Q-ToF) mass spectrometer coupled with an Agilent high-performance liquid chromatography (HPLC)-chip cube system. Peptides were separated on Agilent HPLC-chip consisting of C_18_ enrichment and analytical column (75 μm, 10 cm) system. The LC run was a 6-h-long linear acetonitrile gradient (5% to 50%). Mass spectrometry data were collected in an information-dependent acquisition (IDA) mode over a mass range of 300 to 2,000 *m/z*, and each full MS survey scan was followed by 10 fragment scans. Dynamic exclusion was enabled for all experiments (repeat count, 1; exclusion duration, 30 s). Protein identification and quantitation was carried out using Protein Piolet software 5.0.2. Spectral data were searched against E. coli K-12 MG1655 and A. baumannii ATCC 17978 protein databases downloaded from the NCBI FTP server. Precursor mass tolerance of 0.01 Da and fragment mass tolerance of 40 ppm were allowed. The peptides and proteins were filtered at 1% false discovery rate. Label-free quantitation was performed using the LFQ option in the Protein Piolet software 5.0.2. Protein LFQ intensity value was accepted only when two or more quantifiable peptides were identified in more than 2 replicates per experimental group. Quantitation was performed by taking a ratio of the average intensity with respect to control sample (DMSO treated) (23, 26).

### (iv) *In vitro* susceptibility against ESKAP pathogens.

Antibiotic susceptibility testing was carried out according to the CLSI guidelines for broth microdilution assay. The 10 mg/mL stock solutions of test compounds were prepared in DMSO. Bacterial cultures were inoculated in Mueller-Hinton broth II (MHBII), and the optical density (OD) of the cultures was measured at the 600-nm wavelength, followed by dilution to achieve ~10^5^ CFU/mL. The compounds were tested ranging from 64 to 0.5 mg/L in 2-fold serial diluted fashion with 2.5 μL of each concentration added to each well of a 96-well round bottom microtiter plate. Later, 97.5 μL of bacterial suspension was added to each well containing the test compound along with appropriate controls. The plates were incubated at 37°C for 18 to 24 h following which the growth was enumerated and MIC was identified. The MIC is defined as the lowest compound concentration where there is no visible growth. For each compound, MIC determinations were carried independently 3 times using duplicate samples (44).

### (v) Cell cytotoxicity studies.

MTT [3-(4,5-dimethyl-2-thiazolyl)-2,5-diphenyl-2H-tetrazolium bromide] assay was performed against Vero cells to assess *in vitro* cytotoxicity of the compounds. Approximately 10^3^ cells/well were seeded in a 96-well plate and incubated at 37°C in a 5% CO_2_ atmosphere. After 24 h, compounds and controls were added ranging from 100 to 12.5 μg/mL concentration and incubated for 72 h. After the incubation was over, MTT was added in each well, incubated at 37°C for a further 4 h, residual medium was discarded, 0.1 mL of DMSO was added to solubilize the formazan crystals, and OD was taken at 540 nm for the calculation of CC_50_. CC_50_ is defined as the lowest concentration of compound that leads to a 50% reduction in cell viability. Doxorubicin was used as a positive control, and each experiment was repeated in triplicate (45).

### (vi) Time-kill kinetics of 3a against A. baumannii.

The bactericidal activity of 3a was assessed by the time-kill method. Briefly, A. baumannii BAA-1605 was diluted ~10^5^ CFU/mL in MHBII and treated with 1× and 10× MIC of 3a and polymyxin B and incubated at 37°C with shaking for 24 h. The samples (0.1 mL) were collected at time intervals of 0 h, 1 h, 6 h, and 24 h, serially diluted, and plated on Mueller-Hinton agar (MHA) followed by incubation at 37°C for 18 to 20 h. Kill curves were constructed by counting the colonies from the plates and plotting the CFU/mL of surviving bacteria at each time point in the presence and absence of compound. Each experiment was repeated three time in duplicate, and the mean data is plotted (46).

### (vii) Determination of synergism of 3a with other drugs.

Interaction of 3a with ceftazidime, ceftriaxone, meropenem, polymyxin B, amikacin, tobramycin, minocycline, and rifampicin was tested by the checkerboard method as per CLSI guidelines. Serial 2-fold dilutions of each drug were freshly prepared prior to testing. While the antibiotics were serially diluted along the abscissa in a 96-well microtiter plate, 3a was 2-fold diluted along the ordinate. Ninety-five microliters of ~10^5^ CFU/mL was added to each well, and plates were incubated at 37°C for 18 to 24 h. After the incubation period was over, the ΣFICs (fractional inhibitory concentrations) were calculated as follows: ΣFIC = FIC A + FIC B, where FIC A is the MIC of drug A in the combination/MIC of drug A alone and FIC B is the MIC of drug B in the combination/MIC of drug B alone. The combination is considered synergistic when the ∑FIC is ≤0.5, indifferent when the ∑FIC is >0.5 to 4, and antagonistic when the ∑FIC is >4 (47).

### (viii) Determination of postantibiotic effect of 3a.

To determine the postantibiotic effect (PAE) of 3a, an overnight culture of A. baumannii BAA-1605 was diluted in MHBII at ~10^5^ CFU/mL, exposed to 1× and 5× MIC of 3a and control antibiotics, and incubated at 37°C for 1 h. After incubation, cultures were centrifuged and washed 2 times with prewarmed MHBII to remove any traces of antibiotics. Finally, cells were resuspended in drug-free MHBII and incubated further at 37°C. Samples were taken every 1 h, serially diluted, and plated on Trypticase soy agar (TSA) for enumeration of CFU. The PAE was calculated as PAE = T − C, where T refers to the difference in time required for 1 Log_10_ increase in CFU versus CFU observed immediately after the removal of drug and C is a similarly treated drug-free control (48).

### (ix) Determination of *in vivo* efficacy of 3a in murine neutropenic thigh infection model.

The use of mice for infectious diseases was approved by the Institutional Animal Ethics Committee at the CSIR-Central Drug Research Institute (IAEC/2019/2/Renew-1/Dated-22.06.2020). Prior to initiating the evaluation of the antimicrobial activity, a single 10-mg/kg dose of 3a was given to four Swiss albino mice weighing between 25 and 31 g intraperitoneally as a 100-μL injection. No mortality was observed over the period of 5 days (see Table S6 in the supplemental material). For *in vivo* evaluation of 3a’s antibacterial activity, Swiss mice weighing ~22 to 25 g were used. Neutropenia was caused via intraperitoneally administered 100 μL cyclophosphamide injections (150 mg/kg and 100 mg/kg of body weight) 4 days and 1 day prior to infection. Following neutropenia induction, the right thigh of mice was infected with 100 μL inoculum of ~10^8^ CFU/mL A. baumannii BAA-1605 intramuscularly (i.m.). Starting at 3 h postinfection, 3a (1 mg/kg) and polymyxin B (5 mg/Kg) were injected i.p. into mice, thrice and twice at an interval of 3 h between injections. Control animals were administered saline in the same volume and frequency as those receiving treatment. After 24 h, the mice were sacrificed; thigh tissue was collected, weighed, and homogenized in 5 mL of saline. The homogenate was serially diluted and plated on MHA plates for CFU determination. After incubation for 18 to 24 h at 37°C, CFU were enumerated, and the data was averaged across three experiments (49).

### Determination of induced resistant mutant generation of A. baumannii BAA 1605.

The potential emergence of bacterial resistance against 3a was assessed. The propensity of Acinetobacter baumannii ATCC BAA-1605 to generate resistance was evaluated using serial exposure of organisms to 3a and amikacin in the presence of subinhibitory concentrations and monitored the changes in MIC values over a period of 15 days.

### (i) Cloning of NuOF and its mutants.

Oligonucleotide primers were purchased from Eurofins, and DNA sequencing was also performed by Eurofins and 1st base. Amplification of PCR was performed with Hi-Proof DNA polymerase (Hi-media) using colony PCR for E. coli K-12 MG1655. The NuOF open reading frames was amplified by PCR. The standard PCR mixture (10 μL) contains 100 ng of template DNA and 100 to 250 ng of each forward and reverse primer of gene. The gradient PCR cycling conditions were as follows: 95°C (5 min); 35 cycles of 95°C (45 s), 56.7°C (30 s), 72°C (3 min), and 72°C (5 min).The PCR products were cloned into NdeI/BamHI site of pET-22b(+) vector using the SLIC (sequence- and ligation-independent cloning) method with a C-terminal 6×-His tag (Novagen) to yield plasmid pET22b-NuOF for protein expressions in E. coli BL21(DE3)(50).

For creating the NuOF C180A, C351A, C354A, and C351-354A mutant, a forward primer containing the altered sequence was used along with the T7 reverse primer to amplify the mutated part of the gene from pET-22b(+) containing the wild-type (WT) NuOF in PCR. DpnI enzyme was added to PCR tube and incubated for 1 h at 37°C and transformed into E. coli DH5-α cells.

The standard PCR mixture for megaprimer (10 μL) contains 100 ng of template DNA and 100 to 250 ng of each mutagenic forward primer and T7 reverse primer for mutants. The gradient PCR cycling conditions were as follows: 95°C (3 min); 35 cycles of 95°C (45 s), 56.8°C for C180A, 55.2°C for C351A, 58.7°C for C354A, and 59°C for C351-354A (30 s), and 72°C (3 min) and 72°C (5 min). The PCR products were isolated using Hi-media PCR purification kit. Next, amplification of these megaprimers was done with pET22b-NuOF. The standard PCR mixture (25 μL) contains 100 ng of template DNA and 100 to 250 ng of each mutagenic forward primer and T7 reverse primer for mutants. The gradient PCR cycling conditions were as follows: 95°C (2 min); 35 cycles of 95°C (45 s), 68°C (10 min), 68°C (1 min), and 68°C (15 min). After the PCRs, DpnI (10 U) was added, and the mixture was incubated at 37°C for 1 h. The PCR mixtures were transformed into E. coli competent DH5α cells and plated on LB medium supplemented with 1× ampicillin. All mutations were verified by sequencing analysis.

### (ii) Expression and purification of NuOF and its mutants.

Plasmid pET-22b(+)-NuOF was transformed into E. coli BL21(DE3). The cells were grown in LB broth supplemented with 100 μg/mL of ampicillin at 37°C. For protein expression, 500 mM isopropyl-β-d-thiogalactopyranoside (IPTG) was added to the culture to make a final concentration of 0.5 mM IPTG to induce protein expression when the cells grow to the log phase (OD_600_ ≈ 0.6). The cells were further incubated for 16 h at 16°C for protein expression.

After expression of proteins, cells were harvested by centrifugation (6,000 rpm, 4°C, 20 min) and binding buffer (50 mM Tris, pH 8, at room temperature [RT] and 20 mM imidazole) was added to the pellet in a 1:100 ratio. The cell suspension was sonicated by a tip probe sonicator on ice. The soluble and insoluble protein fractions were separated by centrifugation (18,000 × *g*, 4°C, 20 min) and the supernatant was collected for protein purification. The soluble fraction was passed through a prepacked Ni-nitrilotriacetic acid (Ni-NTA) column (5 mL; GE Life Sciences) prewashed with 10 column volumes (CV) of binding buffer. The column was then washed with 10 CV of wash buffer comprising 50 mM Tris, pH 8, at RT and 20 mM imidazole. The protein was eluted with buffer comprising 50 mM Tris, pH 8, at RT and 500 mM imidazole and dialyzed against 50 mM Tris, pH 8, at RT. Aliquots of the purified proteins were flash frozen in liquid nitrogen and stored at −80°C until further use. All mutants were purified and stored as described above for the wild-type enzyme.

### Cloning of *betB*.

Amplification of the *betB* gene was performed with Hi-Proof DNA polymerase (Hi-media) using PCR for A. baumannii ATCC 17978 genomic DNA. The *betB* open reading frames was amplified by PCR. The standard PCR mixture (10 μL) contains 100 ng of template DNA and 100 to 250 ng of each forward and reverse primer of gene. The gradient PCR cycling conditions were as follows: 95°C (5 min); 35 cycles of 95°C (45 s), 59.2°C (30 s), 72°C (3 min), and 72°C (5 min). The PCR products were cloned into the NdeI/BamHI site of pET-22b(+) vector using SLIC (sequence- and ligation-independent cloning) method with a C-terminal 6×-His tag (Novagen) to yield plasmid pET22b-BetB for protein expressions in E. coli BL21(DE3)(50).

### (i) Expression and purification of *betB*.

Plasmids pET-22b(+)-BetB was transformed into E. coli BL21(DE3). The cells were grown in LB broth supplemented with 100 μg/mL of ampicillin at 37°C. For protein expression, 500 mM IPTG was added to the culture to make a final concentration of 0.5 mM IPTG to induce protein expression when the cells grow to the log phase (OD_600_ ≈ 0.6). The cells were further incubated for 16 h at 18°C for protein expression.

After expression of proteins, cells were harvested by centrifugation (6,000 rpm, 4°C, 20 min) and lysis buffer (50 mM Tris, pH 8, at RT, 500 mM NaCl, 0.5 mM phenylmethylsulfonyl fluoride [PMSF], 10 mM β-mercaptoethanol, and 1% triton 100) was added to the pellet in a 1:100 ratio. The cell suspension was sonicated by a tip probe sonicator on ice. The soluble and insoluble protein fractions were separated by centrifugation (18,000 × *g*, 4°C, 20 min), and the supernatant was collected for protein purification. The soluble fraction was passed through a prepacked Ni-NTA column (5 mL; GE Life Sciences) prewashed with 10 column volumes (CV) of binding buffer (50 mM Tris, pH 8 at RT, 500 mM NaCl, 0.5 mM PMSF, 10 mM β-mercaptoethanol, 1% triton 100, and 10 mM imidazole). The column was then washed with 10 CV of wash buffer (50 mM Tris, pH 8, at RT, 500 mM NaCl, 10 mM β-mercaptoethanol, 25 mM imidazole). The protein was eluted with an elution buffer (50 mM Tris, pH 8, at RT, 500 mM NaCl, 10 mM β-mercaptoethanol, and 300 mM imidazole) and dialyzed against dialysis buffer (50 mM Tris, pH 8, at RT, 300 mM NaCl, 10 mM β-mercaptoethanol). Aliquots of the purified proteins were flash frozen in liquid nitrogen and stored at −80°C until further use.

### Target validation using ABPP.

The protein concentrations for both wild-type and mutant proteins were estimated using a Bradford assay and 5 μM stocks were prepared in dialysis buffer (50 mM Tris, pH 8, at RT) accordingly. One hundred-microliter aliquots were further used for all of the assays. These aliquots were treated with varying concentrations of P1 (0 to 100 μM) at 37°C for 1 h. Following this, the click reaction was performed as described earlier, and the reactions were quenched by adding 40 μL of 4× loading dye. The samples were resolved and activity was visualized using a 10% SDS-PAGE gel using established protocols (23, 26, 51, 52).

### (i) Sequence alignment.

The sequences of NuOE (*A. aeolicus*; UniProtKB ID number O66842; E. coli; UniProtKB ID number P0AFD1) and NuOF (*A. aeolicus*; UniProtKB ID number O66841; E. coli; UniProtKB ID number P31979) were obtained from the UniProt database. The unweighted sequence alignments between NuOE and NuOF from *A. aeolicus* and E. coli were performed using T-coffee at the European Bioinformatics Institute website (https://www.ebi.ac.uk) using the default settings and displayed using Jalview. The sequence name indicates the UniProt code and the organism of origin, and the numbers indicate the amino acid residues displayed. The consensus symbols “*,” “:,” and “.” under the amino acids indicate identical, conserved, and semiconserved residues, respectively.

### Prediction of putative ligand-binding pockets in NuOF protein.

The Computed Atlas of Surface Topography of proteins (CASTp 3.0) web server was used to predict the putative ligand-binding pockets and elucidate the amino acids lining each pocket in the protein. The protein structure of NuOF from *A. aeolicus* (PDB ID number 6Q9K) was submitted in a standard PDB format on the server, and a probe radius of 2.8 Å was used. The CASTp server uses the weighted Delaunay triangulation and the alpha complex method to measure the area and volume of each predicted pocket or void, utilizing both the solvent accessible surface model and molecular surface model. The scoring results of CASTp were provided in Table S9 in the supplemental material.

### (i) *In silico* molecular docking studies.

The energies of the systems were optimized theoretically by using the Gaussian 09 software (53, 54). These energies were computed using density functional theory (DFT) DFT methods by employing the B3LYP function. Energy calculations for highest occupied molecular orbital-lowest unoccupied molecular orbital (HOMO-LUMO) orbitals were performed and calculated using the basis set Lanl2dz for heavier atoms (I, Cl, or S) in conjunction with the 6-31G basis set (for all other atoms). The X-ray crystal structure of NuOF with a resolution of 1.99 Å was retrieved from PDB (PDB ID number 6Q9K). The protein and ligand PDB QT files were prepared using AutoDock Tools 1.5.6 (ADT) following the standard protocol. A grid box (15 × 15 × 15 Å^3^) defined for pocket ID 4 (Table 1, entry D) in NuOF centered at the coordinates *x* = 21.872, *y* = 10.022, and *z* = −77.327 was used for focused docking of flexible ligands (dpI, hpI, dhI) into the active site containing active cysteines (C347 and C350). The docking parameters were set to default except for the following: exhaustiveness = 64, energy range = 3 kcal/mol, and number of modes = 20. The best-scored docking pose with the lowest binding energy was selected for analysis, and figures were visualized using PyMOL (The PyMOL Molecular Graphics System, Version 2.0; Schrödinger, LLC). LigPLOT+ was used to depict the 2D interactions of ligand and protein.

### Electrochemical studies.

The electrochemical reduction experiments were performed with a standard three-electrode setup connected to a CHI760E electrochemical workstation (55). Glassy carbon and platinum wire were used as the working electrode and counter electrode respectively. Ag/AgCl was used as a reference electrode. Stock solutions of compounds 1a and 3a (10 mM in DMSO) were added to respective volumetric flasks and were diluted with electrolyte solution to achieve a final concentration of 0.5 mM. The electrolyte used was tetra-butyl ammonium hexafluorophosphate (TBAP) (0.1 M solution in ACN). The cyclic voltammograms (CV) were recorded at room temperature (20°C) following purging with argon for 2 min at a sweep rate of 100 mV/s in the potential window of −2 V to +2 V.

### HPLC traces.

Stock solutions of compounds (10 mM in DMSO) were prepared. From the stock solution, an aliquot of compounds (100 μM, 500 μL) in acetonitrile was prepared and HPLC analysis was conducted. A diode array detector (DAD) operating at 280 nm was used. An aliquot (100 μM in ACN, 25 μL) was injected and a mobile phase of water/acetonitrile (0.1% HCOOH) was used. A multistep gradient was used with a flow rate of 1 mL/min and a run time of 18 min starting with 40:60 → 0 to 4 min, 40:60 to 20:80 → 4 to 12 min, 20:80 to 40:60 → 12 to 15 min, and 40:60 to 40:60 to 40:60 → 15 to 18 min.

### Statistical analysis.

Statistical analysis was performed using GraphPad Prism 6.0 software (GraphPad Software, La Jolla, CA, USA). Comparison between three or more groups was analyzed using one-way analysis of variance (ANOVA), with *post hoc* Tukey's and Dunnett’s multiple comparisons tests. *P* values of <0.05 were considered to be significant.

### Data availability.

Supporting information includes experimental procedures, biological assay protocols, characterization data, computational protocols, and spectral data is in supplemental material. Proteomics data files are available free of charge.
