# Supplementary material for: Heterocyclic Diaryliodonium-Based Inhibitors of Carbapenem-Resistant Acinetobacter baumannii
Source: Microbiol Spectr. 2023 Mar 28;11(2):e04773-22. doi: 10.1128/spectrum.04773-22 (PMC10101131; doi:10.1128/spectrum.04773-22)

Title: Heterocyclic Diaryliodonium-Based Inhibitors of Carbapenem-Resistant *Acinetobacter baumannii* (CRAB)

Authors: Pooja Kumari,[a] Grace Kaul,[b,c] T. Anand Kumar,[a] Abdul Akhir,[c] Manjulika Shukla,[c] Suraj Sharma,[a] Siddhesh S. Kamat,*[,d] Sidharth Chopra*[,b,c] and Harinath Chakrapani*[,a]

Author Affiliations: [a]Department of Chemistry, Indian Institute of Science Education and Research Pune, Pune 411008, Maharashtra, India. [b]AcSIR: Academy of Scientific and Innovative Research (AcSIR), Ghaziabad 201002, India. [c]Division of Molecular Microbiology and Immunology, CSIR-Central Drug Research Institute, Janakipuram Extension, Sitapur Road, Lucknow-226031, Uttar Pradesh, India. [d]Department of Biology, Indian Institute of Science Education and Research Pune, Pune 411008, Maharashtra, India.

Table of Contents

Table of Contents 2
1. General methods : 3
2. Synthetic procedures and characterization of compounds: 4
3. HPLC traces and area% report 14
4. Supplementary Tables: 18
5. Supplementary Figures: 24
6. References: 31
7. NMR Spectra: 32

1. General methods :

All the chemicals and solvents were purchased from commercial sources and used as received unless stated otherwise. Column chromatography was performed using silica gel-Rankem (60–120 mesh) or silica gel Spectrochem (100-200 mesh) or Rankem neutral alumina as stationary phase. Preparative high performance liquid chromatography (HPLC) was done using Combiflash EZ prep UV using a Kromasil®C-18 preparative column (250 mm × 21.2 mm, 5 µm). ^{1}H and ^{13}C spectra were recorded on a JEOL 400 MHz (or 100 MHz for ^{13}C) or a Bruker 400 MHz (or 100 MHz for ^{13}C) spectrometer unless otherwise specified using either residual solvent signals $CDCl_3$ (δ_H, = 7.26 ppm, δ_C = 77.2 ppm), DMSO-d6 (δ_H, = 2.50 ppm, δ_C = 39.5 ppm) and CD_3OD (δ_H, = 3.31 ppm, δ_C = 49.0 ppm) or as an internal tetramethylsilane (δ_H = 0.00, δ_C = 0.0). Chemical shifts (δ) are reported in ppm and coupling constants (*J*) in Hz. The following abbreviations are used: m (multiplet), s (singlet), d (doublet), t (triplet) and dd (doublet of doublets), dt (doublet of triplets). High-resolution mass spectra were obtained from HRMS-ESI-Q-Time of Flight LC/MS. FT-IR spectra were recorded using Bruker-Alpha-FTIR spectrometer. Photometric measurements were performed using a Thermo Scientific Varioskan microtiter plate reader. Analytical HPLC was performed on an Agilent1260-infinitywith Phenomenex®C-18 reverse phase column (250 mm × 4.6 mm, 5 µm). The key compound was found to be >95% pure as determined by HPLC analysis. HPLC trace for key compound is included in the Supporting Information. The cyclic voltammetry experiments were conducted using a standard three-electrode setup connected to a CHI760E electrochemical workstation.

Bacterial strains and Eukaryotic cell lines and media:

The compounds were screened against multidrug-resistant *A. baumannii* BAA-1605 and the hits were additionally screened against a ESKAPE pathogen panel consisting of *Escherichia coli* ATCC 25922, *Klebsiella pneumoniae* BAA-1705, *Pseudomonas aeruginosa* ATCC

27853 and *Staphylococcus aureus* ATCC 29213 and drug-resistant, clinical *A. baumannii* strains. These strains were procured from BEI/ATCC, USA and routinely cultivated on Mueller-Hinton Agar (MHA). For every experiment, a starting culture was produced by inoculating a single colony picked from agar plate into liquid medium and incubated for overnight at 37 ˚C with continuous shaking. Levofloxacin was purchased from TCI. Vero cells (ATCC CCL-81) were maintained in Dulbecco's Modified Eagle's Medium (DMEM) at 37 ˚C in an atmosphere containing 5% CO_2. DMEM and Fetal Bovine Serum (FBS) were purchased from Lonza (Lonza, USA).

2. Synthetic procedures and characterization of compounds:

Compounds **1a**, **11,**(1) and **2a** (2) were synthesised as per reported protocols.

General procedure for the synthesis of 6a-6e, or 9(1)**:**

To a stirred solution of **5a-5e, or 8** (1g, 1.0 eq.) in EtOH (15 mL) were added arylboronic acid (1.2 eq.), K_3PO_4 (2.5 eq.) and $Pd(PPh_3)_4$ (0.05 eq.). The reaction was refluxed for 16 h under nitrogen atmosphere before EtOH was removed by rotary evaporation. After completion of the reaction as monitored by TLC, the reaction mixture was cooled down from RT before EtOH was removed by rotary evaporation. The residue was dissolved in EtOAc and filtered through celite. The mixture was extracted with EtOAc(3 × 10 mL), and the combined organic layers were washed with H_2O and brine, filtered through anhydrous Na_2SO_4, concentrated by rotary evaporation. The crude product was purified by column chromatography on a silica gel to afford **6a-6e,** or **9** as a yellow liquid.

General procedure for the synthesis of 7a-7e:

To a stirred solution of compound **6a-6e** (1.0 eq.) in EtOH: H_2O [4:1] (15 mL), were added Fe (Powder) (3.0 eq.) and NH_4Cl (3.0 eq.) at RT. Then the reaction was refluxed for 15h.

After completion of the reaction as monitored by TLC, EtOAc was added to the reaction mixture and filtered through celite and the filtrate was evaporated by rotary evaporation. The organic components were extracted with EtOAc (3 X 15 mL) and quenched with $NaHCO_3$. The collected organic phases were filtered through anhydrous Na_2SO_4, concentrated under vacuum to obtain crude amine as a yellow liquid. This crude was subjected to next step without purification. The residue was dissolved in anhydrous MeCN (10 mL) was added *p*-TsOH (3.0 eq.) at 0 ^{o}C. A solution of $NaNO_2$ (2.0 eq.) and KI (2.5 eq.) (dissolved in minimum amount of water) were added dropwise to the reaction mixture. Then the reaction mixture was allowed to stir for the next 10 min at 0 ^{o}C and 12 h at RT. After completion of the reaction as monitored by TLC, H_2O was added to the reaction mixture and quenched with $NaHCO_3$ until pH became 9. Then $Na_2S_2O_3$ was added and the colour of the solution turned to light orange. The organic components were extracted with Et_2O (3 × 15 mL) and collected organic phases were filtered through anhydrous Na_2SO_4, concentrated under vacuum to obtain the crude product as a yellow-coloured residue. The residue was purified by column chromatography on neutral alumina using EtOAc: Hexane (0:100 to 5:95) as the mobile phase to afford **7a-7e** as a dark brown liquid.

General procedure for the synthesis of 10:

To a stirred solution of **9** (1.0 eq.) in anhydrous MeCN (10 mL) was added *p*-TsOH (3.0 eq.) at 0 ^{o}C. A solution of $NaNO_2$ (2.0 eq.) and KI (2.5 eq.) (dissolved in minimum amount of water) were added dropwise to the reaction mixture. Then the reaction mixture was allowed to stir for the next 10 min at 0 ^{o}C and 12 h at RT. After completion of the reaction as monitored by TLC, EtOAc was added to the reaction mixture. The organic components were extracted with EtOAc (3 × 15 mL) and collected organic phases were dried over anhydrous Na_2SO_4, concentrated under vacuum to obtain the crude product as a yellow-coloured

residue. The residue was purified by column chromatography on neutral alumina using EtOAc: Hexane (0:100 to 5:95) as the mobile phase to afford **10** as a dark brown liquid.

General procedure for the synthesis of iodoniumtriflate (2c, 2d, 2e, 2g, 3b):

To a stirred solution of aryl iodide(1 eq.) in anhydrous CH_2Cl_2 (5 mL) were added *m*-CPBA (1 eq.), TfOH (3 eq.). The solution was stirred for 1 h at 0 ^oC before CH_2Cl_2 was removed by rotary evaporation. To the residue Et_2O was added, and the mixture was stirred for 20 min, and filtered. The obtained solid was washed with ice cold H_2O and Et_2O three times, dried in vacuum to afford **2c, 2d, 2e, 2g, 3b** as a grey-white solid

General procedure for the synthesis of iodonium chloride (2b, 2f, 3a):

To a stirred solution of aryl iodide (1 eq.) in anhydrous CH_2Cl_2 (5 mL) were added *m*-CPBA (1 eq.), TfOH (3 eq.). The solution was stirred for 1 h at 0 ^oC before CH_2Cl_2 was removed by rotary evaporation. The residue was dissolved in HCOOH (0.5 mL) was added saturated aq. NaCl (2 mL), and the mixture was stirred for 20 min at 0 ^oC, and filtered. The collected solid was washed with ice cold H_2O and Et_2O three times, dried in vacuum to afford **2b, 2f, 3a** as a grey-white solid.

Synthesis of 2-(prop-2-yn-1-ylcarbamoyl) dibenzo[b,d]iodol-5-ium chloride (P1):

To a stirred solution of **3** (310 mg, 0.85 mmol) in anhydrous CH_2Cl_2 (5 mL) was added *m*-CPBA (151 mg, 0.87 mmol), TfOH (228 µL, 2.57 mmol). The solution was stirred for 1 h at RT before $CH_2Cl_2$was removed by rotary evaporation. To the residue mixture dissolved in HCOOH (0.5 mL) was added saturated aq. NaCl (2 mL), and the mixture was stirred for 20 min, and filtered. The collected solid was washed with ice cold H_2O and Et_2O three times, dried in vacuum to afford **P1** as a white solid in 32 % yield.

Preparation and Characterization of Compounds:

3-Nitro-2-phenylpyridine (**6a**): Starting from **5a** (882 mg, 5.56 mmol), **6a** (556 mg, 50%) was isolated as a brown solid:^{1}H NMR (400 MHz, $CDCl_3$) δ: 8.85 (dd, *J* = 4.7, 1.6 Hz, 1H), 8.14 (dd, *J* = 8.2, 1.6 Hz, 1H), 7.58 – 7.55 (m, 2H), 7.48 – 7.46 (m, 3H), 6.84 (dd, *J* = 8.2, 4.7 Hz, 1H); ^{13}C NMR (100 MHz, $CDCl_3$) δ: 153.0, 152.2, 146.4 136.4, 132.3, 129.9, 128.9, 128.2, 122.5.

4-(3-fluorophenyl)-3-nitropyridine (**6b**): Starting from **5a** (1 g, 6.31 mmol), **6b** (590 mg, 43%) was isolated as a brown solid: ^{1}H NMR (400 MHz, $CDCl_3$) δ: 8.87 (dd, *J* = 4.8, 1.7 Hz, 1H), 8.17 (dd, *J* = 8.2, 1.4 Hz, 1H), 7.50 – 7.39 (m, 2H), 7.34 – 7.28 (m, 2H), 7.20 – 7.15 (m, 1H).

3-Nitro-2-(*m*-tolyl) pyridine (**6c**): Starting from **5a** (1 g, 6.31 mmol), **6c** (770 mg, 57%) was isolated as a brown solid: ^{1}H NMR (400 MHz, $CDCl_3$) δ: 8.85 (dd, *J* = 4.7, 1.6 Hz, 1H), 8.12 (dd, *J* = 8.2, 1.6 Hz, 1H), 7.44 – 7.40 (m, 2H), 7.35 – 7.27 (m, 3H), 2.41 (s, 3H).

2-(2-Methoxyphenyl)-3-nitropyridine (**6d**): Starting from **5a** (1 g, 6.31 mmol), **6d** (780 mg, 53%) was isolated as a brown solid: ^{1}H NMR (400 MHz, $CDCl_3$) δ: 8.87 (dd, *J* = 4.8, 1.6 Hz, 1H), 8.21 (dd, *J* = 8.2, 1.6 Hz, 1H), 7.67 (dd, *J* = 7.6, 1.8 Hz, 1H), 7.46 – 7.40 (m, 2H), 7.14 (td, *J* = 7.5, 1.0 Hz, 1H), 6.91 (dd, *J* = 8.3, 0.9 Hz, 1H), 3.71 (s, 3H).

3-Nitro-4-phenylpyridine (**6e**): Starting from **5b** (1 g, 4.93 mmol), **6e** (320 mg, 26%) was isolated as a brown solid: ^{1}H NMR (400 MHz, $CDCl_3$) δ: 9.07 (s, 1H), 8.80 (d, *J* = 5.0 Hz, 1H), 7.49 – 7.46 (m, 3H), 7.42 (d, *J* = 5.0 Hz, 1H), 7.36 – 7.32 (m, 2H).

2-(Thiophen-3-yl)pyridin-3-amine (**9**): Starting from **8** (600 mg, 4.66 mmol), **9** (366 mg, 45%) was isolated as a brown solid: ^{1}H NMR (400 MHz, $CDCl_3$) δ: 8.10 (dd, *J* = 3.7, 2.2 Hz, 1H), 7.70 (dd, *J* = 4.5, 1.2 Hz, 1H), 7.53 (dd, *J* = 4.9, 1.2 Hz, 1H), 7.43 (dd, *J* = 5.0, 2.8 Hz, 1H), 7.07-7.02 (m, 2H), 3.92 (s, 2H); ^{13}C NMR (100 MHz, $CDCl_3$) δ140.9, 140.2, 140.1,

140.0, 128.2, 126.2, 123.9, 123.0, 123.0; HRMS Calcd for $C_9H_8N_2S^+$ $[M+H]^+$: 177.0486 found $[M+H]^+$: 177.0486.

3-Iodo-2-(thiophen-3-yl)pyridine (10): Starting from **9** (350 mg, 1.98 mmol), **10** (300 mg, 53%) was isolated as a dark brown liquid: ^{1}H NMR (400 MHz, $CDCl_3$) δ 8.59 (dd, J = 4.6, 1.6 Hz, 1H), 7.81 (dd, J = 2.9, 1.2 Hz, 1H), 7.52 (dd, J = 5, 1.3 Hz, 1H), 7.37 (dd, J = 5.0, 2.9 Hz, 1H), 6.93 (dd, J = 7.9, 4.6 Hz 1H); ^{13}C NMR (100 MHz, $CDCl_3$) δ 156.9, 148.7, 148.1, 142.6, 128.9, 126.6, 125.0, 123.2, 93.7; HRMS Calcd for $C_9H_6INS^+$ $[M+H]^+$: 287.9344 found $[M+H]^+$: 287.9348.

Benzo [4, 5]iodolo[3,2-b]pyridin-5-ium chloride (2a):Starting from **7a** (150 mg, 0.53 mmol), 2**a** (51 mg, 30%) was isolated as a white solid: ^{1}H NMR (400 MHz, DMSO-d_6) δ: 8.94 (dd, J = 4.6, 1.2 Hz, 1H), 8.90 (dd, J = 8.2, 1.2Hz, 1H), 8.64 (d, J = 7.6 Hz, 1H), 8.42 (dd, J = 7.6, 1.4 Hz, 1H), 7.87-7.78 (m, 2H), 7.70 (dd, J = 8.2, 4.6 Hz, 1H); ^{13}C NMR (100 MHz, DMSO-d_6) δ 157.9, 151.5, 139.8, 139.6, 132.8, 130.9, 130.7, 128.1, 125.8, 125.1, 122.3; FTIR (ν_{max} ,cm^{-1}); 3042, 1568, 1398, 1012, 739; HRMS m/z [M+]calcd for $C_{11}H_7IN^+$, 279.9618, found, 279.9626.

8-Fluorobenzo[4,5]iodolo[3,2-b]pyridin-5-ium chloride (2b): Starting from **7b** (50 mg, 0.167 mmol), **2b** (12 mg, 21%) was isolated as a white solid: ^{1}H NMR (400 MHz, DMSO-d6) δ: 8.95-8.91 (m, 2H), 8.67 (dd, J = 8.9, 5.1 Hz, 1H), 8.15 (dd, J = 9.0, 2.9 Hz, 1H), 7.75-7.67 (m, 2H); ^{13}C NMR (100 MHz, DMSO-d6) δ: 163.7 (d, J = 246 Hz), 156.1 (d, J = 3.3 Hz), 151.1, 141.8 (d, J = 8.6 Hz), 139.2, 132.3 (d, J = 8.6 Hz), 125.8, 122.4, 119.6 (d, J = 23.5 Hz), 119.1, 113.7 (d, J = 24.3 Hz); FTIR (ν_{max} ,cm^{-1});2917, 2857, 1734, 543; HRMS m/z $[M+H]^+$calcd for $C_{11}H_6FIN^+$, 297.9523, found, 297.9527.

8-Fluorobenzo[4,5]iodolo[3,2-b]pyridin-5-ium trifluoromethanesulfonate (2c): Starting from **7b** (114 mg, 0.381 mmol), **2c** (40 mg, 23%) was isolated as a white solid: ^{1}H NMR (400

MHz, DMSO-d6) δ: 9.02 (dd, *J* = 4.6, 1.3 Hz, 1H), 8.61 (dd, *J* = 8.4, 1.4 Hz, 1H), 8.30 (dd, *J* = 9, 4.9 Hz, 1H), 8.22 (dd, *J* = 8.9, 2.9 Hz, 1H), 7.80-7.73 (m, 2H); ^{13}C NMR (100 MHz, DMSO-d6) δ: δ: 164.0 (d, *J* = 247 Hz), 156.5 (d, *J* = 3.1 Hz), 151.7, 142.1 (d, *J* = 8.9 Hz), 139.5, 132.5 (d, *J* = 9.0 Hz), 126.6, 120.6 (d, *J* = 24.0 Hz), 120.3, 115.8, 114.8 (d, *J* = 24.7 Hz); ^{19}F NMR (400 MHz, DMSO-*d6*) δ: -77.76, -109.98; FTIR (ν_{max} ,cm^{-1}); 1225, 1022, 635; HRMS m/z [M+]calcd for $C_{11}H_6FIN^+$, 297.9523, found, 297.9533.

8-Methylbenzo[4,5]iodolo[3,2-b]pyridin-5-ium trifluoromethanesulfonate (**2d**): Starting from **7c** (285 mg, 0.965 mmol), **2d** (128 mg, 30%) was isolated as a white solid:^{1}H NMR (400 MHz, DMSO-d6) δ: 8.98 (dd, *J* = 4.6, 1.3 Hz, 1H), 8.50 (dd, *J* = 8.4, 1.3 Hz, 1H), 8.39 (d, *J* = 1.2 Hz, 1H), 8.06 (d, *J* = 8.5 Hz, 1H), 7.72-7.68 (m, 2H), 2.61 (s, 3H); ^{13}C NMR (100 MHz, CD_3OD) δ: 159.1, 152.6, 143.2, 140.7, 139.7, 135.2, 130.4, 130.3, 126.5, 119.2, 117.3, 20.8; FTIR (ν_{max} ,cm^{-1}); 1225, 1026, 634; HRMS m/z [M+H]$^+$calcd for $C_{11}H_6MeIN^+$, 293.9774, found, 293.9785.

9-Methoxybenzo[4,5]iodolo[3,2-b]pyridin-5-ium trifluoromethanesulfonate (**2e**): Starting from **7d** (194 mg, 0.623 mmol), **2e** (60 mg, 21%) was isolated as a white solid:^{1}H NMR (400 MHz, CD_3OD) δ 8.93 (dd, *J* = 4.6, 1.3 Hz, 1H), 8.53 (dd, *J* = 8.3, 1.3 Hz, 1H), 8.32 (d, *J* = 8.7 Hz, 1H), 7.81 (d, *J* = 2.4 Hz, 1H), 7.67 (dd, *J* = 8.3, 4.6 Hz, 1H), 7.48 (dd, *J* = 8.7, 2.4 Hz, 1H), 3.94 (s, 1H); ^{13}C NMR (100 MHz, CD_3OD) δ 163.0, 159.9, 152.1, 140.0, 134.3, 127.7, 125.6, 122.9, 121.8, 117.5, 115.8, 57.1; ^{19}F NMR (400 MHz, CD_3OD) δ -77.76.HRMS m/z [M+H]$^+$ calcd for $C_{11}H_6$ OMeIN$^+$, 309.9723, found, 309.9730.

Benzo[4,5]iodolo[2,3-c]pyridin-9-ium chloride (**2f**): Starting from **7e** (85 mg, 0.302 mmol), **2f** (31 mg, 33%) was isolated as a white solid:^{1}H NMR (400 MHz, DMSO-d6) δ: 9.58 (s, 1H), 8.9 (d, *J* = 5.2 Hz, 1H), 8.7 (dd, *J* = 8.1, 1.0 Hz, 1H), 8.59 (dd, *J* = 7.8, 1.4 Hz, 1H), 8.43 (dd, *J* = 5.3, 0.6 Hz, 1H), 7.89 (td, *J* = 7.3/7.7, 1.1 Hz, 1H) 7.82-7.78 (m, 1H); ^{13}C NMR

(100 MHz, DMSO-$_{d6}$) δ 150.6, 149.8, 148.7, 139.7, 132.5, 130.9, 130.5, 127.5, 125.9, 121.9, 120.3; FT-IR (ν_{max} ,cm^{-1}); 3049.01, 1567.39, 1384.43, 979.14, 606.18;HRMS m/z $[M+H]^+$calcd for $C_{11}H_7IN^+$, 279.9618, found, 279.9625.

Benzo[4,5]iodolo[2,3-c]pyridin-9-ium trifluoromethanesulfonate (2g): Starting from **7e** (175 mg, 0.622 mmol), **2g** (60 mg, 22%) was isolated as a white solid:^{1}H NMR (400 MHz, DMSO-d6) δ: 9.29 (s, 1H), 8.98 (d, *J* = 5.2 Hz, 1H), 8.66 (dd, *J* = 7.9, 1.4 Hz, 1H), 8.50 (dd, *J* = 5.3, 0.6 Hz, 1H), 8.27 (dd, *J* = 8.2, 0.9 Hz,1H), 7.96-7.92 (m, 1H), 7.87-7.82 (m, 1H); ^{13}C NMR (100 MHz, DMSO-d6) δ: 150.6, 150.5, 149.4, 140.4, 133.5, 131.1, 131.0, 128.4, 123.8, 121.3, 119.5; ^{19}F NMR (400 MHz, DMSO-$_{d6}$) δ: -77.76; FTIR (ν_{max} ,cm^{-1}); 2984, 1736, 1372, 1233, 1043, 633; HRMS m/z [M+] calcd for $C_{11}H_7IN^+$, 279.9618, found, 279.9625.

Thieno[2',3':4,5]iodolo[3,2-b]pyridin-4-ium chloride (**3a**): Starting from **10** (280 mg, 0.975 mmol), **3a** (147 mg, 47%) was isolated as a white solid:^{1}H NMR (400 MHz, DMSO-d6) δ: 9.09 (dd, *J* = 9.0, 1.4 Hz, 1H), 8.80 (dd, *J* = 4.7, 1.4 Hz, 1H), 8.0 (d, *J* = 5.2 Hz, 1H), 7.74 (d, *J* = 5.3, Hz, 1H), 7.50 (dd, *J* = 8.3, 4.6 Hz 1H); ^{13}C NMR (100 MHz, DMSO-$_{d6}$) δ: 154.1, 150.3, 146.5, 139.1, 135.6, 126.1, 124.0, 123.7, 123.1; HRMS Calcd for $C_9H_6INS^+$ $[M]^+$: 285.9182found $[M]^+$: 285.9193.

Thieno[3',2':4,5]iodolo[3,2-b]pyridin-8-ium trifluoromethanesulfonate (**3b**): Starting from **10** (270 mg, 0.940 mmol), **3b** (105 mg, 26%) was isolated as a white solid:^{1}H NMR (400 MHz, DMSO-d6) δ: 8.91, (dd, *J* = 4.6, 1.2 Hz, 1H), 8.58 (dd, *J* = 8.5, 1.3 Hz, 1H), 8.17 (d, *J* = 5.4 Hz, 1H), 7.83 (d, *J* = 5.4, Hz, 1H), 7.60 (dd, *J* = 8.3, 4.6 Hz 1H); ^{13}C NMR (100 MHz, DMSO-d6) δ: 153.7, 151.1, 147.9, 139.1, 137.1, 124.5, 124.0, 122.3, 114.7; ^{19}F NMR (400 MHz, DMSO-$_{d6}$) δ-77.75; HRMS Calcd for $C_9H_6INS^+$ $[M]^+$: 285.9182found $[M]^+$: 285.9197.

2-(Prop-2-yn-1-ylcarbamoyl) dibenzo[b,d]iodol-5-ium chloride (**P1**): Starting from **4** (310 mg, 0.858 mmol), **P1** (110 mg, 32%) was isolated as a white solid: ^{1}H NMR (400 MHz, DMSO-d6) δ 9.25 (t, J = 5.3 Hz, 1H), 8.84 (s, 1H), 8.59 (d, J = 7.8 Hz, 1H), 8.47 (d, J = 7.8 Hz, 1H), 8.07 (d, J = 8.5 Hz, 1H), 7.86 (t, J = 7.6 Hz, 1H), 7.71 (t, J = 7.6 Hz, 1H), 4.15 (d, J = 2.9 Hz, 2H), 3.20 (t, J = 2.6 Hz, 1H); ^{13}C NMR (100 MHz, DMSO-d6) δ 164.8, 143.8, 140.6, 130.8, 130.5, 130.4, 128.4, 127.0, 126.0, 125.3, 124.6, 81.1, 73.2, 28.8; FT-IR (υ_{max}, cm^{-1}) 3374, 3201, 1602, 1318, 1077, 610; HRMS Calcd for $C_{16}H_{11}INO^+$ [M]$^+$: 359.9880 found [M]$^+$: 359.9890.

Synthesis of 6-iodo-[1,1'-biphenyl]-3-carboxylic acid (12):

To a stirred solution of compound **11** (500 mg, 1.42 mmol) in EtOH: H_2O [1:1] (5 mL), were added KOH (199 mg 3.55 mmol) at RT and the reaction was stirred for 4 h at 80 °C. After completion of the reaction as monitored by TLC, the reaction mixture was cooled down from RT to 0 °C. The reaction mixture was quenched with 4 M HCl until pH became acidic. Then EtOAc was added to the reaction mixture. The organic components were extracted with EtOAc (3 X 15 mL). The collected organic phases were dried over anhydrous Na_2SO_4, concentrated under vacuum to obtain pure product **12** (454 mg, 99%) as a grey yellow solid.

Synthesis of 6-iodo-N-(prop-2-yn-1-yl)-[1,1'-biphenyl]-3-carboxamide (4):

To a stirred solution of compound **2** (300 mg, 0.9256 mmol) in dry DCM: THF (9:1, 10 mL) were added EDC.HCl (195 mg, 1.018 mmol), DMAP (28 mg, 0.2314 mmol) and propargyl amine (65 µL, 1.018 mmol) and stirred for 12 h at RT. Then water was added to the reaction mixture. The organic components were extracted with DCM, dried over Na_2SO_4 and concentrated. The residue was purified by column chromatography on a silica gel (0-17 % EtOAc/Hexane) to afford the white solid **4** with 61 % yield.

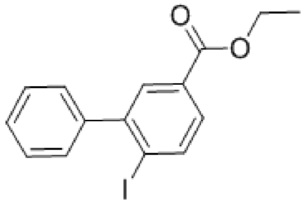

11

ethyl 6-iodo-[1,1'-biphenyl]-3-carboxylate (11):^{1}H NMR (400 MHz, $CDCl_3$) δ: 8.11 (d, *J* = 7.9 Hz, 2H), 7.97 (d, *J* = 7.8 Hz, 1H), 7.43-7.39 (m, 3H), 7.29 (d, *J* = 7.6 Hz, 1H), 7.06 (t, *J* = 7.4 Hz, 1H), 4.1 (q, *J* = 7.0 Hz, 2H), 1.41 (t, *J* = 7.0 Hz, 3H); ^{13}C NMR (100 MHz, $CDCl_3$) δ: 166.5, 148.6, 145.8, 139.8, 130.0, 129.8, 129.5, 129.4, 128.4, 98.0, 61.2, 14.5.

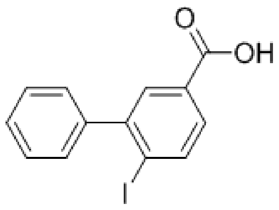

12

6-iodo-[1,1'-biphenyl]-3-carboxylic acid (12):^{1}H NMR (400 MHz, DMSO-$_{d6}$)δ:13.0 (s, 1H), 8.02-7.99 (m, 3H), 7.49 (dt, *J* = 7.5, 1.1 Hz, 1H), 7.45(d, *J* = 8.4 Hz, 2H), 7.36 (dd, *J* = 7.6, 1.5 Hz, 1H), 7.16 (dt, *J* = 7.6, 1.6 Hz, 1H), ^{13}C NMR (100 MHz, DMSO-$_{d6}$) δ: 167.1, 147.9, 145.1, 139.4, 130.0, 129.4, 129.1, 128.6, 98.2.

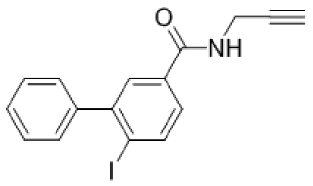

4

6-iodo-N-(prop-2-yn-1-yl)-[1,1'-biphenyl]-3-carboxamide (4):^{1}H NMR (400 MHz, $CDCl_3$) δ: 7.96 (dd, *J* = 7.9, 1.0 Hz, 1H), 7.86-7.84 (m, 2H), 7.43-7.41 (m, 2H), 7.39 (dd, *J* = 7.5, 1.1 Hz, 1H), 7.28 (dd, *J* = 7.6, 2.0 Hz, 1H), 7.06 (dt, *J* = 7.7, 1.7 Hz, 1H), 6.38 (s, 1H), 4.29 (dd, *J*= 6.0, 2.6 Hz, 2H), 2.30 (t, *J* = 2.6 Hz, 1H); ^{13}C NMR (100 MHz, $CDCl_3$) δ: 166.9, 147.7, 145.6, 139.8, 133.0, 130.1 129.8, 129.4, 128.4, 126.9, 98.1, 79.5, 72.2, 30.0.

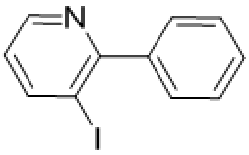

7a

3-iodo-2-phenylpyridine (7a): ^{1}H NMR (400 MHz, $CDCl_3$) δ: 8.63 (dd, *J* = 4.7, 1.5 Hz, 1H), 8.26 (dd, *J* = 8.2, 1.5 Hz, 1H), 7.60 – 7.57 (m, 2H), 7.48-7.42 (m, 3H), 6.97 (dd, *J* = 8.0, 4.7 Hz, 1H).

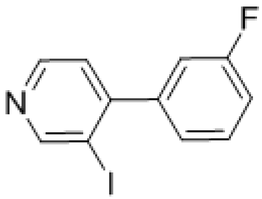

7b

4-(3-fluorophenyl)-3-iodopyridine (7b):^{1}H NMR (400 MHz, $CDCl_3$) δ: 8.63 (dd, *J* = 4.6, 1.5 Hz, 1H), 8.26 (dd, *J* = 8.0, 1.5 Hz, 1H), 7.45 – 7.36 (m, 2H), 7.32-7.28 (m, 1H), 7.15-7.10 (m, 1H), 7.01 (dd, *J* = 8.0, 4.6 Hz, 1H).

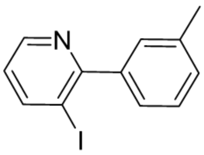

7c

3-iodo-2-(*m*-tolyl) pyridine (7c):^{1}H NMR (400 MHz, $CDCl_3$) δ: 8.61 (dd, *J* = 4.7, 1.6 Hz, 1H), 8.24 (dd, *J* = 8.0, 1.6 Hz, 1H), 7.39 – 7.30 (m, 3H), 7.22 (d, *J* = 7.6 Hz, 1H), 6.95 (dd, *J* = 8.0, 4.7 Hz, 1H), 2.41 (s, 3H).

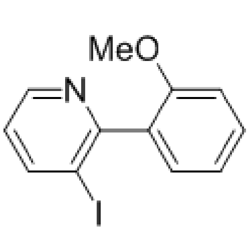

7d

3-iodo-2-(2-methoxyphenyl) pyridine (7d):^{1}H NMR (400 MHz, $CDCl_3$) δ: 8.64 (dd, *J* = 4.7, 1.6 Hz, 1H), 8.19 (dd, *J* = 8.0, 1.6 Hz, 1H), 7.43 – 7.39 (m, 1H), 7.23 (dd, *J* = 7.4, 1.8 Hz, 1H), 7.06 (td, *J* = 7.4, 1.0 Hz, 1H), 6.99-6.96 (m, 2H), 3.80 (s, 3H).

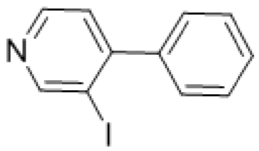

7e

3-iodo-4-phenylpyridine (7e):^{1}H NMR (400 MHz, $CDCl_3$) δ: 9.04 (s, 1H), 8.54 (d, *J* = 4.9 Hz, 1H), 7.48 - 7.45 (m, 3H), 7.36 –7.34 (m, 2H), 7.27 (dd, *J* = 5, 0.6 Hz, 1H).

3. HPLC traces and area% report: (For key compounds)

The compounds are found to be ≥95% pure, as determined by HPLC analysis. The detector wavelength used was 280 nm.

HPLC trace of **P1** (100 µM) in acetonitrile:

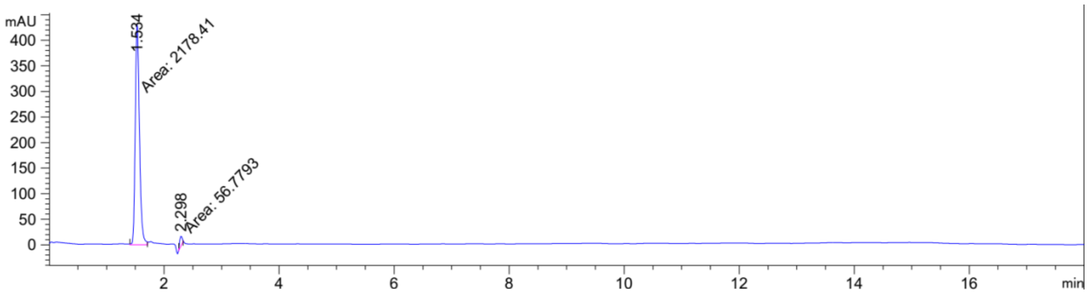


```
=====================================================================
                      Area Percent Report
=====================================================================

Sorted By             :      Signal
Multiplier            :      1.0000
Dilution              :      1.0000
Use Multiplier & Dilution Factor with ISTDs


Signal 1: DAD1 A, Sig=280,4 Ref=off

Peak RetTime Type  Width     Area      Height     Area
  #   [min]        [min]   [mAU*s]     [mAU]        %
----|-------|----|-------|----------|----------|--------|
   1   1.534 MM    0.0839 2178.41455  432.60638  97.4598
   2   2.298 MM    0.0500   56.77927   18.92602   2.5402

Totals :                  2235.19382  451.53241
```

HPLC trace of **3a** (100 µM) in acetonitrile:

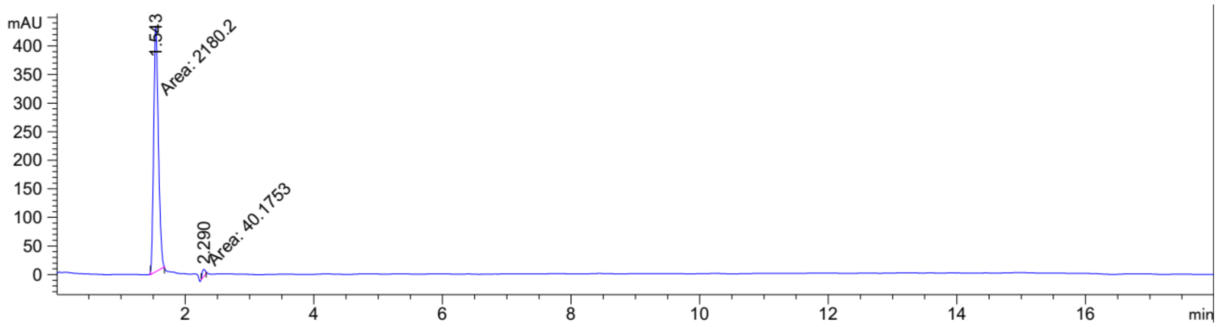


```
=====================================================================
                         Area Percent Report
=====================================================================

Sorted By             :      Signal
Multiplier            :      1.0000
Dilution              :      1.0000
Use Multiplier & Dilution Factor with ISTDs


Signal 1: DAD1 A, Sig=280,4 Ref=off

Peak RetTime Type  Width     Area      Height     Area
  #   [min]        [min]    [mAU*s]     [mAU]       %
----|-------|----|-------|----------|----------|--------|
   1   1.543 MM    0.0843 2180.19922  431.14145  98.1906
   2   2.290 MM    0.0523   40.17532   12.79218   1.8094

Totals :                  2220.37454  443.93363
```

HPLC trace of **2g** (100 µM) in acetonitrile:

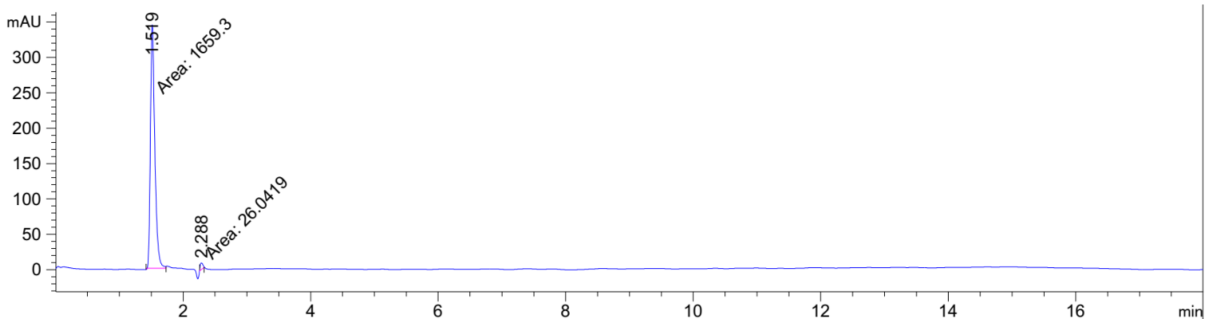


```
=====================================================================
                         Area Percent Report
=====================================================================

Sorted By             :      Signal
Multiplier            :      1.0000
Dilution              :      1.0000
Use Multiplier & Dilution Factor with ISTDs


Signal 1: DAD1 A, Sig=280,4 Ref=off

Peak RetTime Type  Width     Area      Height     Area
  #   [min]        [min]    [mAU*s]     [mAU]       %
----|-------|----|-------|----------|----------|--------|
   1   1.519 MM    0.0801 1659.30103  345.42682  98.4548
   2   2.288 MM    0.0448   26.04193    9.68649   1.5452

Totals :                  1685.34295  355.11331
```

HPLC trace of **2e** (100 µM) in acetonitrile:

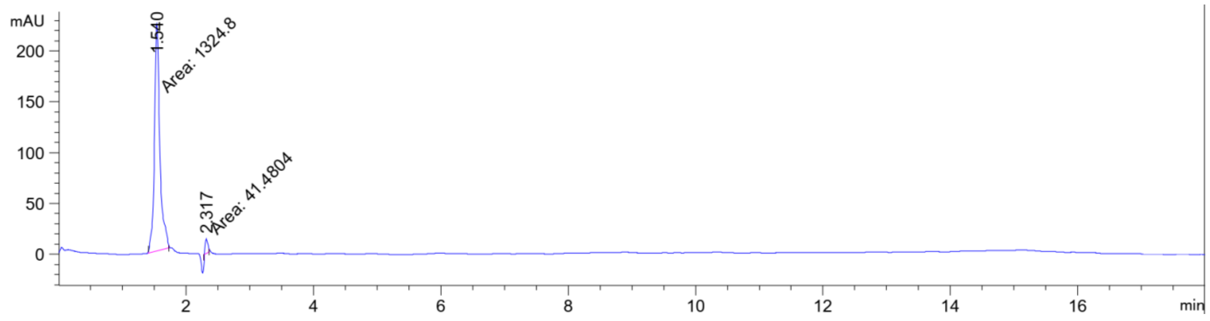

Area Percent Report

Sorted By : Signal
Multiplier : 1.0000
Dilution : 1.0000
Use Multiplier & Dilution Factor with ISTDs

Signal 1: DAD1 A, Sig=280,4 Ref=off

Peak #	RetTime [min]	Type	Width [min]	Area [mAU*s]	Height [mAU]	Area %
1	1.540	MM	0.0985	1324.80273	224.25330	96.9640
2	2.317	MM	0.0478	41.48039	14.47635	3.0360
Totals :				1366.28312	238.72964	

HPLC trace of **2d** (100 µM) in acetonitrile:

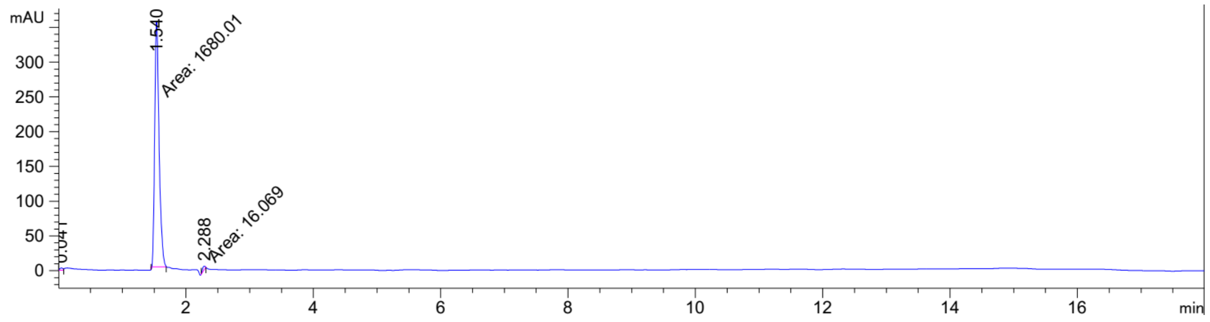

Area Percent Report

Sorted By : Signal
Multiplier : 1.0000
Dilution : 1.0000
Use Multiplier & Dilution Factor with ISTDs

Signal 1: DAD1 A, Sig=280,4 Ref=off

Peak #	RetTime [min]	Type	Width [min]	Area [mAU*s]	Height [mAU]	Area %
1	0.041	BV	0.0505	10.91324	3.30281	0.6393
2	1.540	MM	0.0790	1680.00842	354.31934	98.4193
3	2.288	MM	0.0473	16.06902	5.65981	0.9414
Totals :				1706.99068	363.28196	

HPLC trace of **2b** (100 µM) in acetonitrile:

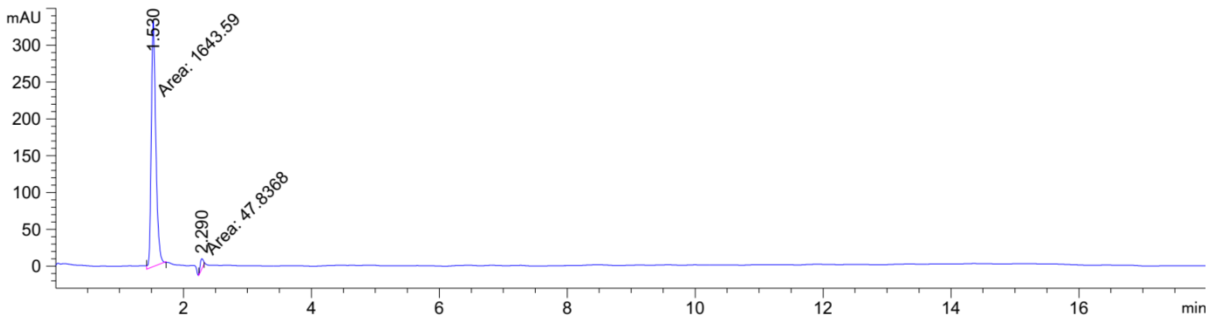


```
=====================================================================
                          Area Percent Report
=====================================================================

Sorted By             :      Signal
Multiplier            :      1.0000
Dilution              :      1.0000
Use Multiplier & Dilution Factor with ISTDs


Signal 1: DAD1 A, Sig=280,4 Ref=off

Peak RetTime Type  Width     Area      Height     Area
  #   [min]        [min]   [mAU*s]     [mAU]        %
----|-------|----|-------|----------|----------|--------|
   1   1.530 MM    0.0819 1643.59033  334.61649  97.1718
   2   2.290 MM    0.0542   47.83684   14.71494   2.8282

Totals :                  1691.42717  349.33143
```

HPLC trace of **2a** (100 µM) in acetonitrile:

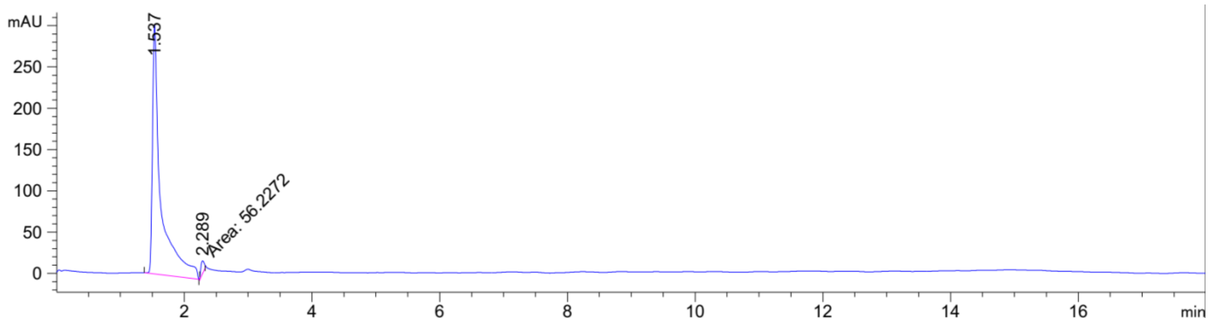


```
=====================================================================
                          Area Percent Report
=====================================================================

Sorted By             :      Signal
Multiplier            :      1.0000
Dilution              :      1.0000
Use Multiplier & Dilution Factor with ISTDs


Signal 1: DAD1 A, Sig=280,4 Ref=off

Peak RetTime Type  Width     Area      Height     Area
  #   [min]        [min]   [mAU*s]     [mAU]        %
----|-------|----|-------|----------|----------|--------|
   1   1.537 BB    0.1238 2735.04321  301.73746  97.9856
   2   2.289 MM    0.0581   56.22718   16.13600   2.0144

Totals :                  2791.27040  317.87346
```

HPLC trace of **1a** (100 µM) in acetonitrile:

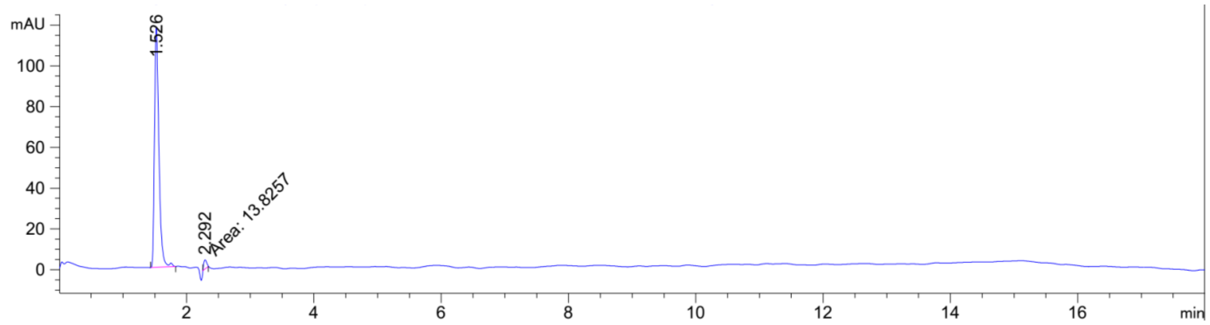


```
==================================================================
                         Area Percent Report
==================================================================

Sorted By             :      Signal
Multiplier            :      1.0000
Dilution              :      1.0000
Use Multiplier & Dilution Factor with ISTDs


Signal 1: DAD1 A, Sig=280,4 Ref=off

Peak RetTime Type  Width     Area      Height     Area
  #   [min]        [min]   [mAU*s]     [mAU]        %
----|-------|----|-------|----------|----------|--------|
   1   1.526 BV R  0.0735  567.82434  118.21446  97.6230
   2   2.292 MM    0.0500   13.82574    4.60583   2.3770

Totals :                   581.65008  122.82029
```

4. Supplementary Tables:

Table S1: Cytotoxicity profile of iodonium compounds against Vero cells (ATCC CCL-81).

**Entry**	**Cpd.**	**MIC**(µg/mL) **against** ***A. baumannii*** **BAA-1605**	**Selectivity index (SI=** CC_{50}/MIC**)**
1	**2a**	0.125	<1
2	**2c**	0.125	<10
3	**2d**	0.25	<10
4	**3a**	0.0625	>20
5	**3b**	0.0625	>20

Table S2: MIC of hpI compounds against MDR clinical isolates of *A. baumannii*

S.No	Strain	Cat. No.	1a	3a	3b	AMK	CAZ	LVX	MEM	MIN	PMB	TOB
1	H72721	NR-9667	2	0.5	0.125	16	64	8	64	4	0.25	1
2	Isolate 1	NR-13374	2	0.5	0.125	16	>64	32	32	8	0.5	1
3	Isolate 2	NR-13375	2	0.5	0.125	8	>64	>64	>64	8	0.5	<0.5
4	Isolate 3	NR-13376	1	0.25	0.0625	>64	>64	>64	32	8	0.5	<0.5
5	Isolate 4	NR-13377	2	0.25	0.125	4	>64	>64	64	8	0.25	<0.5
6	Isolate 5	NR-13378	2	0.5	0.125	64	>64	4	>64	16	0.5	>64
7	Isolate 6	NR-13379	1	0.5	0.125	64	>64	4	>64	16	0.5	>64
8	Isolate 7	NR-13380	2	0.5	0.125	64	>64	8	1	64	0.5	>64
9	Isolate 8	NR-13381	1	0.25	0.125	64	>64	4	>64	16	0.5	>64
10	Isolate 9	NR-13382	1	0.5	0.125	64	>64	4	>64	16	0.125	>64
11	Isolate 10	NR-13383	2	0.5	0.125	>64	>64	>64	32	8	0.5	8
12	Isolate 11	NR-13384	1	0.25	0.125	>64	>64	32	>64	8	0.5	1
13	Isolate 12	NR-13385	2	0.5	0.125	>64	>64	4	>64	16	0.5	>64
14		NR-17777	4	0.5	0.25	>64	64	8	2	4	0.5	>64
15	5-032	NR-17778	1	0.5	0.125	8	4	<0.5	1	0.5	0.5	<0.5
16	5-109	NR-17780	4	0.5	0.125	>64	64	8	2	4	0.5	>64
17	5-143	NR-17781	1	0.5	0.125	16	>64	1	2	<0.5	0.5	8
18	5-189	NR-17782	2	0.5	0.125	>64	>64	8	1	16	0.5	64
19	BC-5	NR-17783	2	0.5	0.125	>64	>64	16	>64	8	0.5	8
20	Naval-17	NR-17784	2	0.5	0.125	16	>64	16	2	64	0.25	<0.5
21	Naval-18	NR-17785	2	0.25	0.125	>64	32	4	>64	4	0.25	64
22	Naval-81	NR-17786	2	0.5	0.25	>64	32	8	2	4	0.25	64
23	WL-136	NR-19298	2	0.25	0.125	64	4	<0.5	1	0.5	0.5	1
24	WC-487	NR-19299	2	0.5	0.125	8	2	<0.5	0.5	<0.5	0.5	<0.5
25	Control	BAA-1605	1	0.25	0.125	8	>64	8	8	4	0.5	1
26	Control	ATCC-17978	2	0.125	0.0625	2	2	<0.5	<0.5	<0.5	0.5	<0.5

Amikacin: AMK, Ceftazidime: CAZ, Levofloxacin: LVX, Meropenem: MEM, Minocycline: MIN, Polymyxin B: PMB, Tobramycin: TOB. colour code: Red: Resistant, Blue: Intermediate, and yellow: susceptible

Table S3: Drug combination studies of **3a**with antibiotics/ compounds against *A. baumannii* BAA-1605

Drug/ Compound	Average FIC	Inference
Polymyxin B	2.00	No interaction
Meropenem	1.75	No interaction
Amikacin	0.33	Synergistic
Tobramycin	0.23	Synergistic
Minocycline	1.75	No interaction
Rifampicin	2.00	No interaction
Levofloxacin	0.56	No interaction
Disulfiram	3.00	No interaction

Average FIC: average of FIC **3a** + FIC B; B: antibiotic, from two independent experiments

Table S4: Drug combination studies with known antibiotics against *E. coli* ATCC 25922

Drug/ Compound	Average FIC	Inference
Polymyxin B	0.68	No interaction
Meropenem	2	No interaction
Amikacin	0.50	Synergistic
Tobramycin	0.50	Synergistic
Minocycline	0.75	No interaction
Rifampicin	0.88	No interaction
Levofloxacin	0.97	No interaction
Ceftazidime	0.75	No interaction

Average FIC: average of FIC **3a** + FIC B; B: antibiotic, from two independent experiment

Table S5: *In vitro* Post Antibiotic effect (PAE) of compounds at different concentrations against *A.baumannii*BAA-1605.

Treatments	Time for 1 $\log_{10}$(h)	PAE (h)
Ctrl	~1.5	0
**1a** 1x MIC	~1.5	0
**1a** 10x MIC	~2	~0.5

**3a** 1x MIC	~2	~0.5
**3a** 10x MIC	~2.5	~1
Amikacin 1x MIC	~2	~0.5
Amikacin 10x MIC	~3	~1.5

Table S6: Maximum Tolerable Dose (MTD) of **3a**

Sample	Weight(g)					
	Day 0	Day 1	Day 2	Day 3	Day 4	Day 5
Mice 1	30	31	31	33	33	34
Mice 2	31	31.5	32	33	33.5	35
Mice 3	30	30	30	31	31	33.5
Mice 4	25	25	26.5	29	30.5	32.5

A single 10 mg/kg dose of **3a** was given to four swiss albino mice weighing between 25-31g intraperitoneally (IP). No mortality was observed over the period of observation

Table S7: MIC values of the iodonium compounds against mycobacterial pathogens

		MIC (µg/mL)			
**Entry**	**Cpd.**	***M. tuberculosis* H37Rv ATCC 27294**	***M. abscessus* ATCC 19977**	***M. fortuitum* ATCC 6841**	***M. chelonae* ATCC 35752**
1	**1a**	0.03	0.125	0.125	0.25
2	**3a**	0.06	0.25	0.03	0.03
3	Isoniazid	0.03	NT	NT	NT
4	Rifampicin	0.06	NT	NT	NT
5	Streptomycin	1	NT	NT	NT
6	Ethambutol	1	NT	NT	NT
7	Levofloxacin	0.12	2	0.06	0.06
8	Amikacin	0.12	4	1	1

Table S8. Primers for wild typeNuOF, cysteine mutants of NuOF and wild type betB

Entry	Primer name	Plasmid constructed	Primer Sequence (5′ → 3′)
1	NuOF_ Fwd	WT NuOF	GAAGGAGATATACATAATGAAAA ACATTAT CCGTAC
2	NuOF_Rev	WT NuOF	AGCTCGAATTCGGATCCCAGCGCT CTTTCAG
3	C180A Fwd	NuOF_C180A	GGGCAGGGCGCTACATCGCCGGG GAAGAAA CAGCG
4	C351A Fwd	NuOF_C351A	TTT TTC GCC CGT GAG TCC GCC GGC TGG TGT ACG CCG TG
5	C354A Fwd	NuOF_C354A	GTGAGTCCTGCGGCTGGGCTACGC CGTGCCGCGACG G
6	C351-C354A Fwd	NuOF_C351-C354A	GTTTTTCGCCCGTGAGTCCGCCGG CTGGGCTACGCCGTG CCGCGACGGTC
7	T7 Terminator Rev		GCTAGTTATTGCTCAGCGG
8	betB_ Fwd	WT betB	TTA AGA AGG AGA TAT ACA TAA TGA GTG ATG TAC AAG TTC
9	betB_ Rev	WT betB	ACGGAGCTCGAATTCGGATCAAA AATGCTTTGATAATCG

Table S9. CASTp based scoring of the identified pockets in NuoF

Entry	Pocket ID	Area (SA)	Volume (SA)	Volume color	Representation style
A	1	563.314	522.663	Salmon red	Cartoon
B	2	98.003	56.840	Marine blue	
C	3	49.050	23.079	Cyan	
D	4	54.962	15.739	Green	
E	5	46.156	14.152	Yellow	
F	6	27.315	10.843	Warm-pink	
G	7	17.831	7.053	Not shown	
H	8	18.387	5.883	Not shown	
I	9	16.695	5.878	Not shown	
J	10	17.429	4.831	Not shown	

Table S10. Top-scored docked poses of ligands dPI (**1a**); hPI (**2a**) and dHI (**3a**) and their interaction with the amino acid residues in the active site (Pocket ID 4) of the NuOF (PDB: 6Q9K)

**Ligand**	**Affinity (kcal/mol)**	**Amino acid residues involved in the interaction**
dpI (**1a**)	-4.1	T390, I392, L201
hpI (**2a**)	-4.0	T390, I392, L201
dhI (**3a**)	-3.6	T390, I392, L201, T351

Table S11: Identified targets of **3a** in *A. baumannii*

**Entry**	**Accession**	**Name**	**log2 FC**	**- log10 (p value)**
1	WP_000840835.1	Glutamate synthase subunit β (gltD)	3.38234	2.370173
2	WP_001286300.1	betaine aldehyde dehydrgenase (betB)	2.961814	3.863535
3	WP_136040424.1	FAD-dependent oxidoreductase	2.776662	1.905118
4	WP_171249601.1	alkyl hydroperoxide reductase subunit F (ahpF)	1.477672	2.4707

Table S12. Electrochemical parameters of **1a** and **3a** (10 mM in DMSO) in 0.1 M TBAP solution versus Ag/AgCl

**Entry**	**Compounds**	**Onset reduction potential (V)**
1	**1a**	-0.853
2	**3a**	-0.711 and -1.057

5. Supplementary Figures:

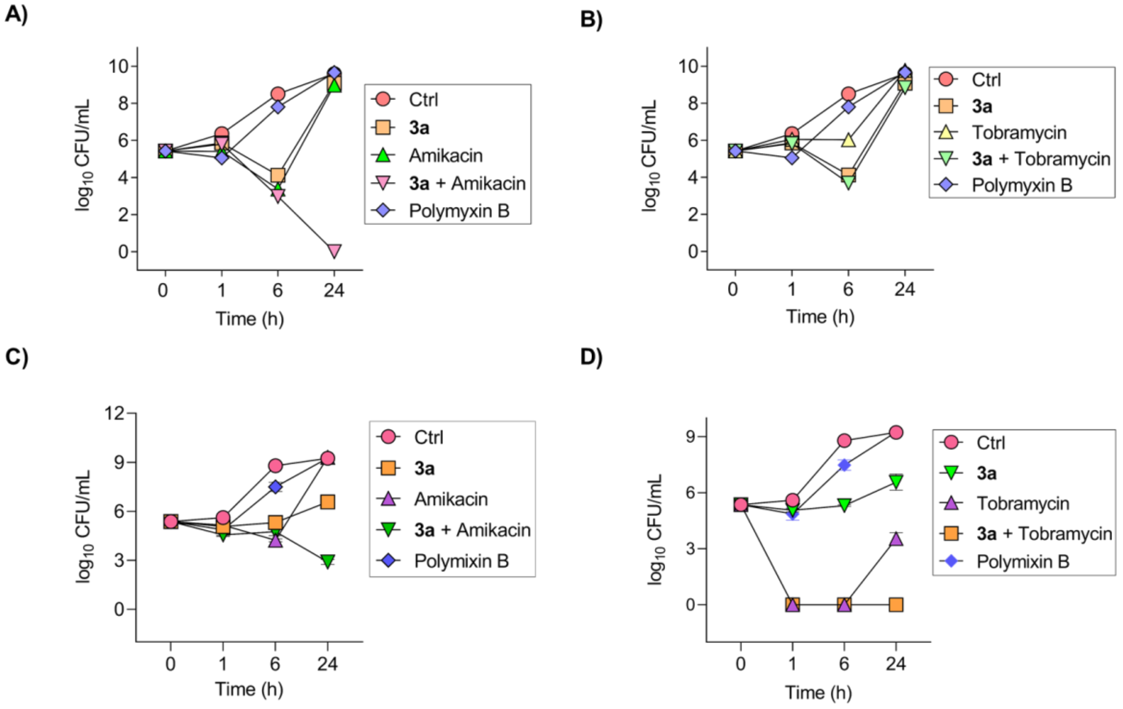

Figure S1: Drug combination study with known antibiotics (**A**) Amikacin and **3a** (1x MIC); (**B**) Tobramycin and **3a** (1x MIC) at against *A. baumannii* BAA-1605. (**C**) Amikacinand **3a** (1x MIC); (**D**) Tobramycin and **3a** at 1x MIC against *E. coli* HM 339 (amikacin susceptible but tobramycin intermediate resistant *E. coli* HM339)

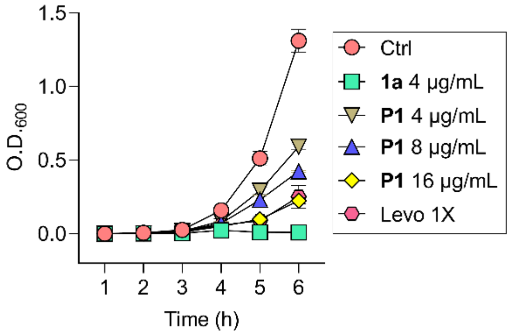

Figure S2: Growth curve of *E. coli* ATCC 25922 treated with P1 (16 µg/mL) shows growth inhibition; Ctrl indicates bacterial cells; **1a** (4 µg/mL); Levo1xindicates Levofloxacin (MIC, 0.0156 µg/mL).

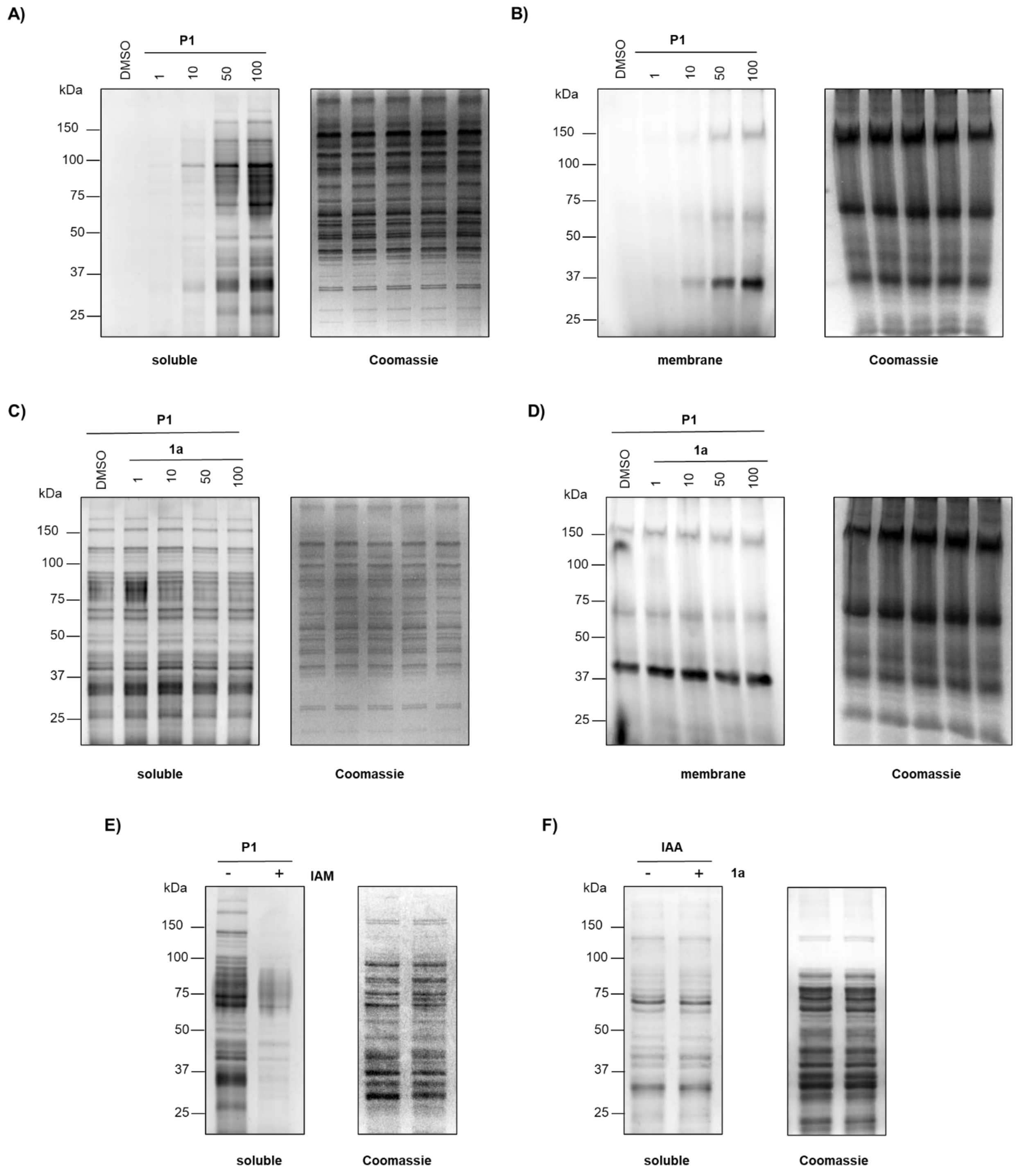

Figure S3: Dose-dependent protein labelling by **P1** (1-100 µM)(**A**) in the membrane and (**B**) soluble proteomic fraction of *E. coli* ATCC 25922.Proteome profiling by **P1** (100 µM) of *E. coli* (**C**) in the soluble and (**D**) membrane proteomic fraction pre-treated with DMSO or **1a** (1-100 µM); (E) Protein labelling by **P1** (100 µM) in pre-treated with DMSO or iodoacetamide (**IAM**, 10 mM) soluble proteomic fraction; (**F**) Protein labelling by **IAA** (100 µM) in pre-treated with DMSO or iodoacetamide (**1a**, 250 µM) soluble proteomic fraction

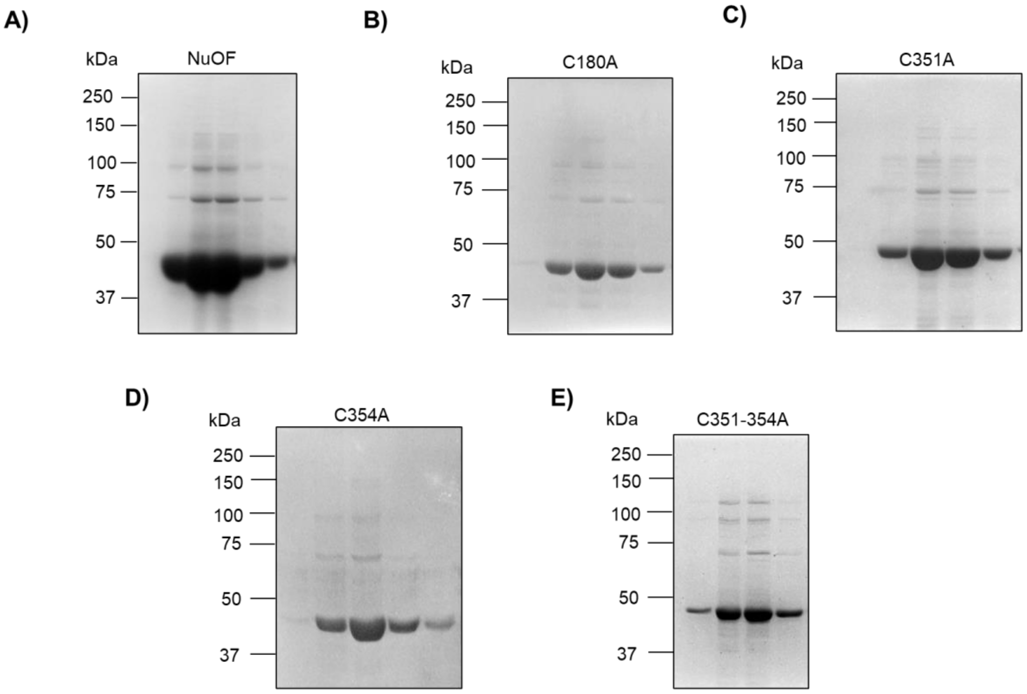

Figure S4: SDS-PAGE gel image for the purified protein (**A**) WT NuOF, (**B**) C180A, (**C**) C351A, (**D**) C354A and (**E**) C351-354A mutant.

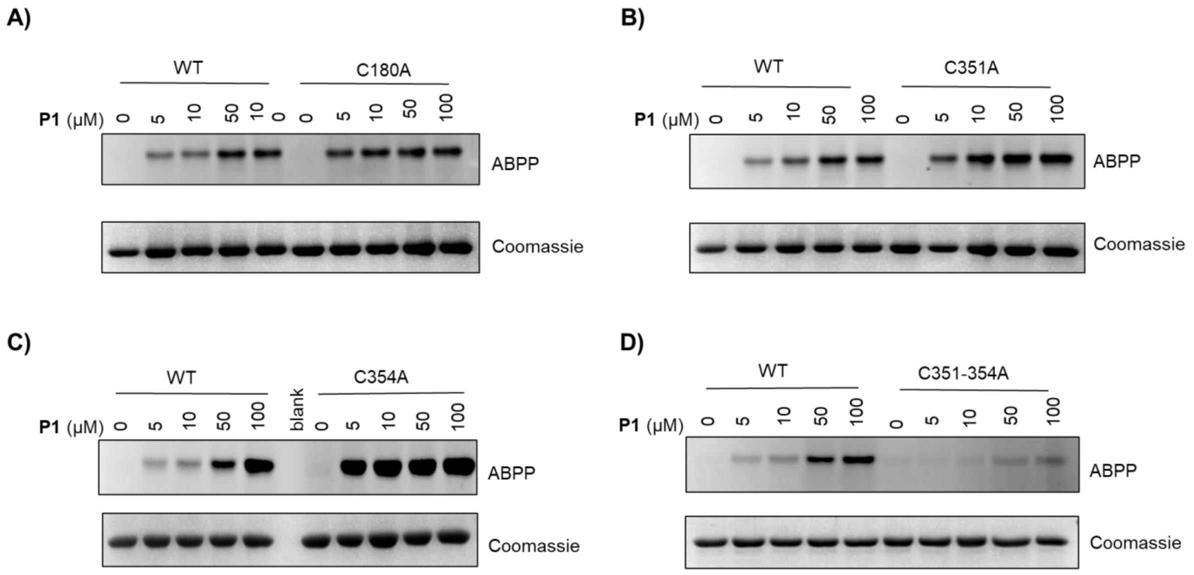

Figure S5: Dose-dependent labeling of NuOF (WT) with varying concentrations of probe **P1**, as compared with their alanine point variants (**A**) C180A, (**B**) C351A, (**C**) C354A and (**D**) C351-354A.

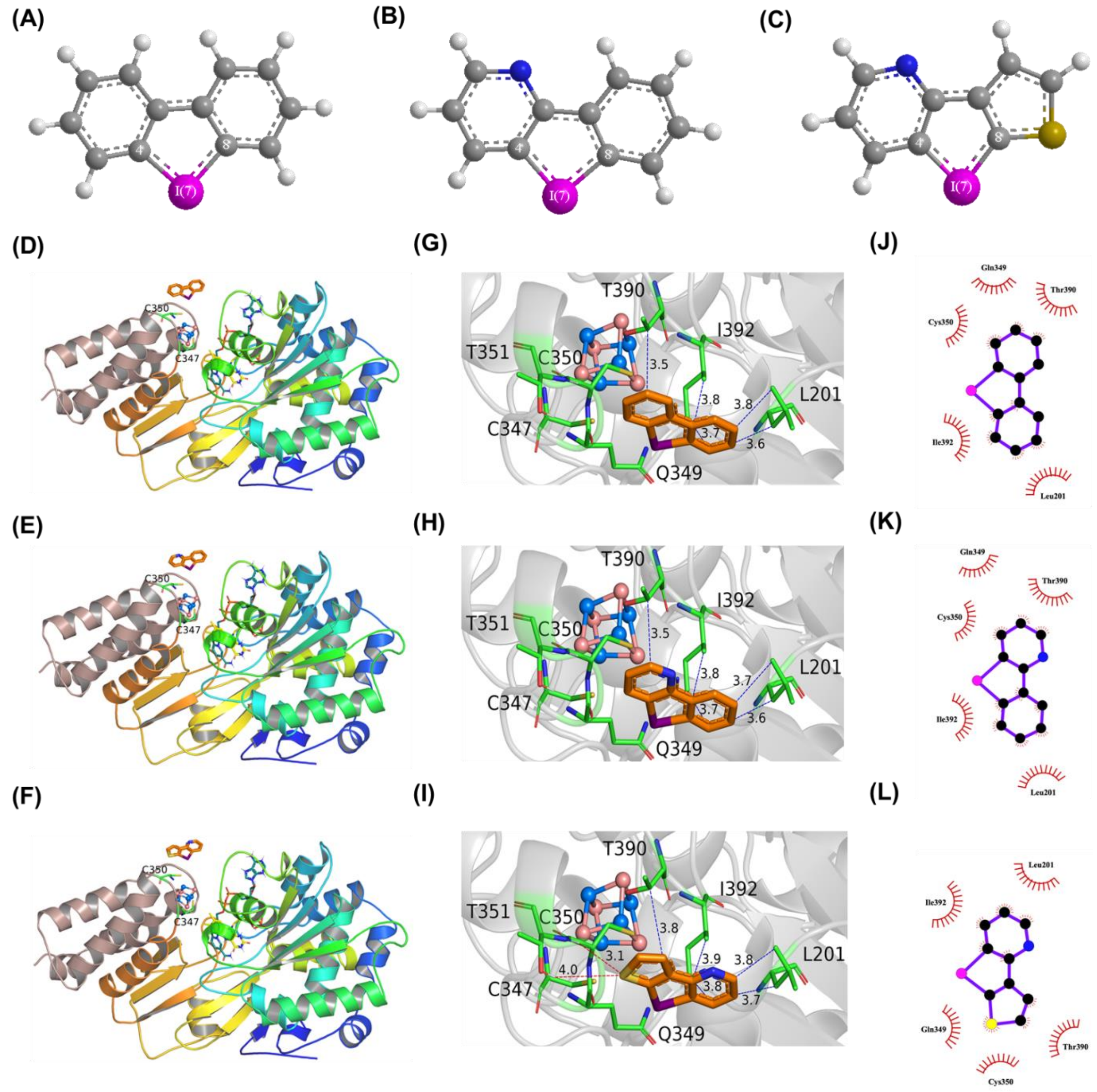

Figure. S6. Minimized energy structures of (**A**) **dpI**(**1a**), (**B**) **hpI** (**2a**) and (**C**) **dhI**(**3a**)calculated by RB3LYP/STO-3G and LUMO energy obtained for **dpI**,**hpI** and **dhI**were 26.16 meV, 13.97 meV and 24.85 meV respectively. Although all the molecules exhibited planarity, the C-I bond lengths of **dhI** are slightly shortened compared to corresponding bond lengths of **dpI**and**hpI**. While the theoretically calculated bond lengths of C(4)-I(7) and C(8)-I(7) for **dpI** and **hpI** were 2.00 Å and 2.00 Å respectively, the C(4)-I(7) and C(8)-I(7) bond lengths of 1.99 Å and 1.89 Å were obtained for **dhI**. *Insilico* docking analysis showing the (**D-F**) Cartoon representation and (**G-I**) Close view of docked ligands: **dPI** (top), **hPI** (middle) and **dHI** (bottom) into the active site (pocket ID 4) with reactive C350 into the catalytic site of NuOF (PDB code: 6Q9K) The protein is depicted in ribbon

style, Fe-S cluster is represented in ball and stick model, while the docked ligand and the residues (indicated by one-letter code) are shown in stick representation. The blue dotted lines indicate hydrophobic interactions, and the lengths are indicated. The figures were generated using PyMOL v 2.0. **(J-L)** LigPlot showing the 2D interactions of ligands: **dPI** (top), **hPI** (middle) and **dHI**(bottom) with NuOF. The molecule is represented in stick model with carbon, nitrogen, iodine and sulphur in black, blue, pink and yellow color respectively. Active site residues are labelled by 3-letter code and represented as ball and stick model. The residues forming hydrophobic interactions are shown as red arcs while the hydrogen bonds are shown as green dashed lines with indicated bond lengths.

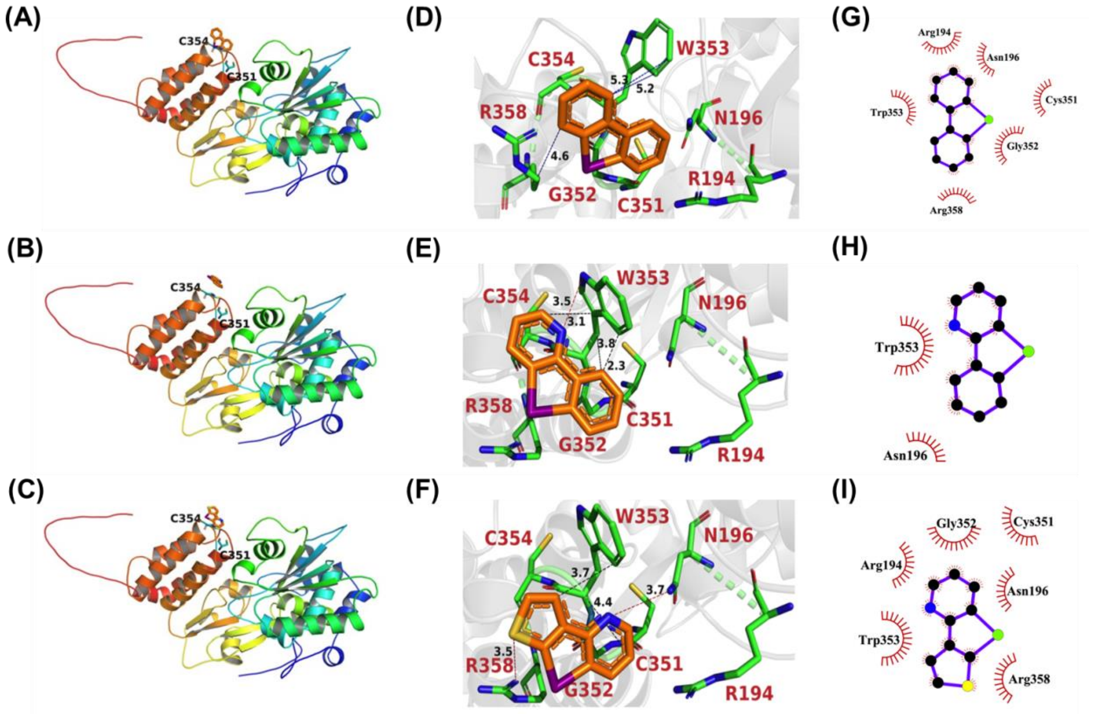

Figure. S7. Insilico docking analysis showing the (A-C) Cartoon representation and (G-I) Close view of docked ligands: dPI (top), hPI (middle) and dHI (bottom) into the active site (pocket ID 7) with reactive C351 and C354 into the catalytic site of of alpha-fold predicted model of NuoF protein from E. coli (P31979). All the considerations followed to represent the ligand, protein and active site residues were the same as indicated in Fig. S6.

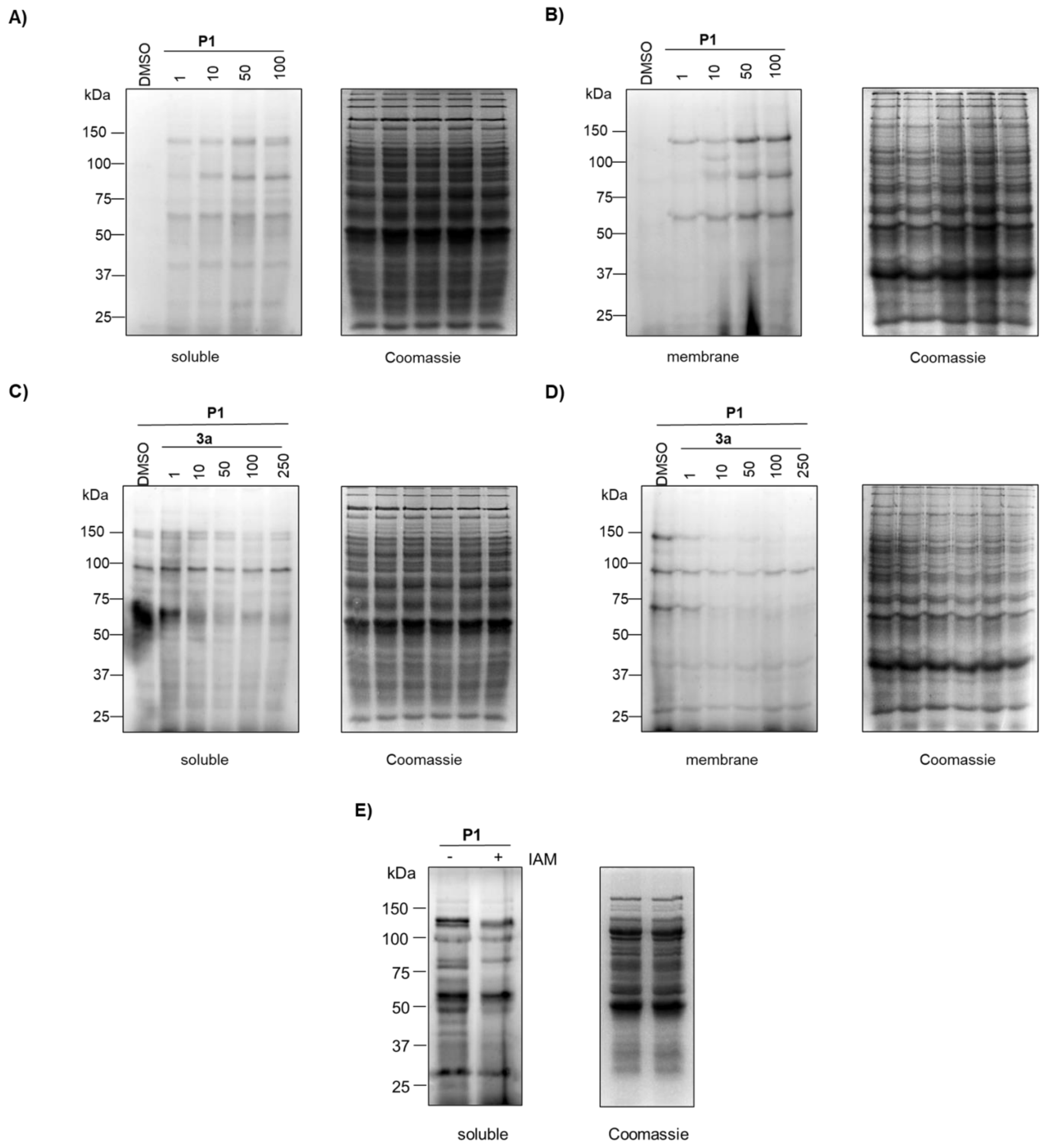

Figure S8: Dose-dependent protein labelling by **P1** (1-100 µM)(**A**) in the membrane and (**B**) soluble proteomic fraction of *A. baumannii* ATCC 17978.Proteome profiling by **P1** (100 µM) of *A. baumannii* (**C**) in the soluble and (**D**) membrane proteomic fraction pre-treated with DMSO or **3a** (1-250 µM); (**E**) Protein labelling by **P1** (100 µM) in pre-treated with DMSO or iodoacetamide (**IAM**, 10 mM) soluble proteomic fraction.

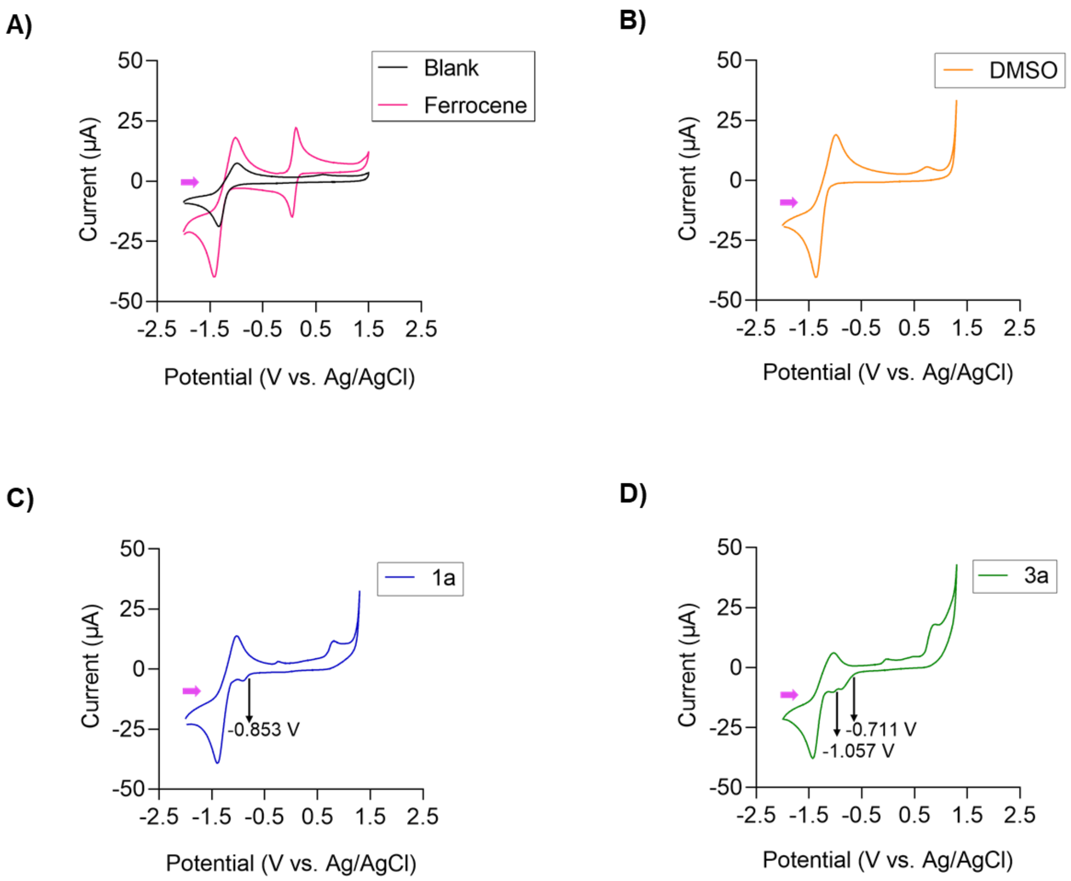

Figure S9: Cyclic voltammogram of (**A**) reversible redox reaction of ferrocene; (**B**) DMSO, (**C**) **1a** and (**D**) **3a** using the following conditions: glassy carbon as working electrode, platinum electrode as counter electrode and Ag/AgCl as reference electrode with an initial positive scan (represented by a pink arrow) at a rate of 100 mV/s; sample interval = 1 mV; quiet time = 2 s; sensitivity = 1e^{-5} A/V; 0.1 M TBAP as the background electrolyte in dry ACN purged with Argon for 2 min.The final concentration of the analytes was 0.5 mM. The onset reduction potentials are shown with arrows in the voltammogram. Blank indicates 0.1 M TBAP in ACN solution.

6. References:

1. Liu Z, Zhu D, Luo B, Zhang N, Liu Q, Hu Y, Pi R, Huang P, Wen S. 2014. Mild Cu(I)-Catalyzed Cascade Reaction of Cyclic Diaryliodoniums, Sodium Azide, and Alkynes: Efficient Synthesis of Triazolophenanthridines. Org Lett 16:5600–5603.
2. Letessier J, Detert H. 2012. First Synthesis of Benzopyridoiodolium Salts and Twofold Buchwald-Hartwig Amination for the Total Synthesis of Quindoline. Synthesis (Stuttg) 2012:290–296.

7. NMR Spectra:

^{1}H NMR of **6a**

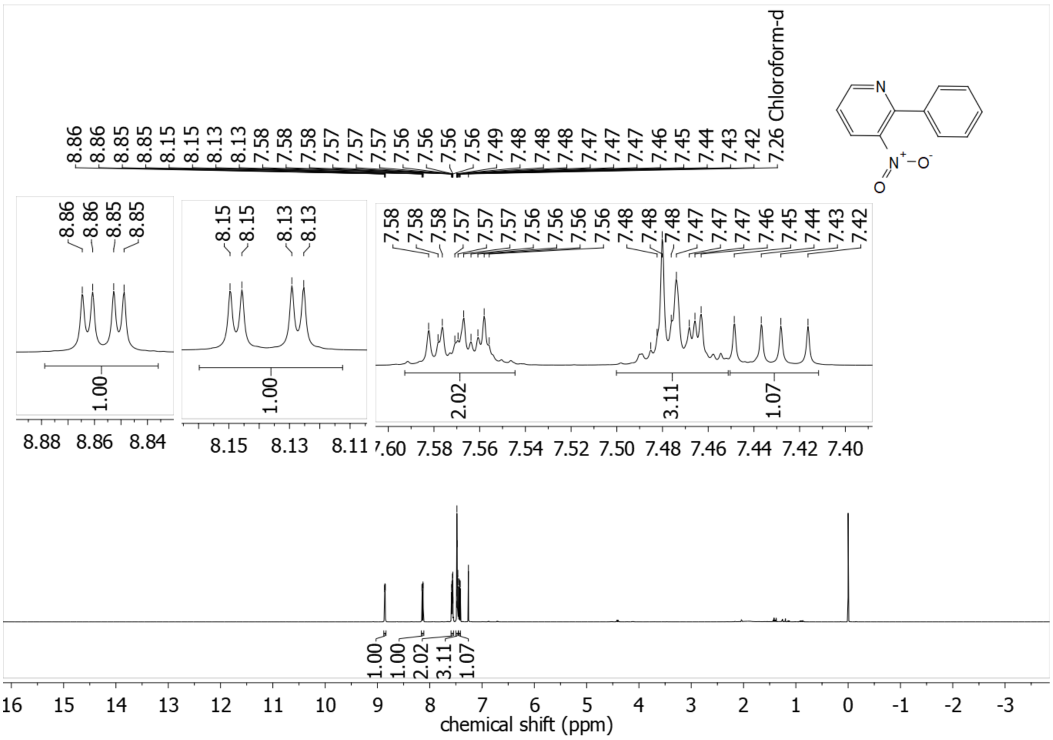

^{1}H NMR of **6b**

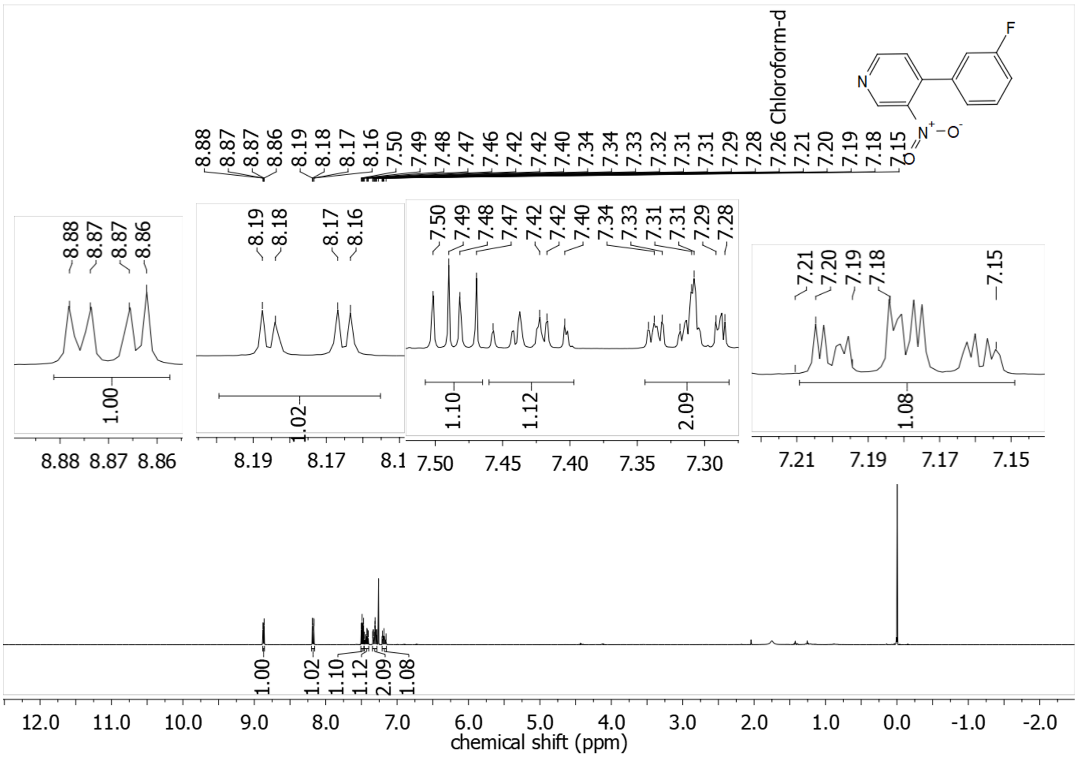

^{1}H NMR of **6c**

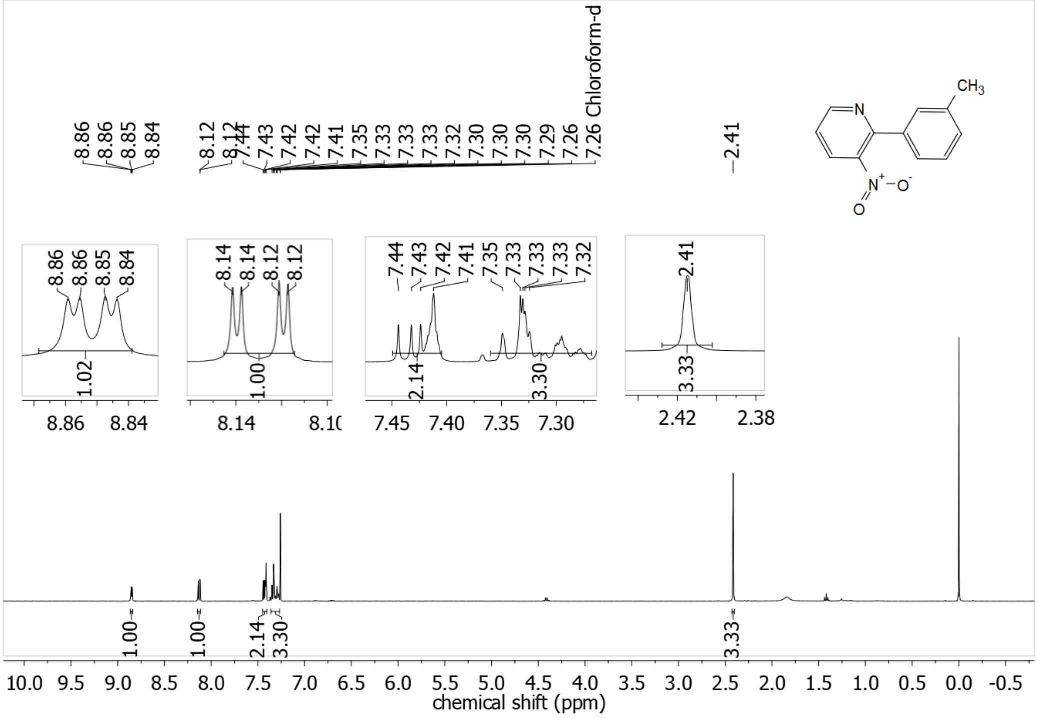

^{1}H NMR of **6d**

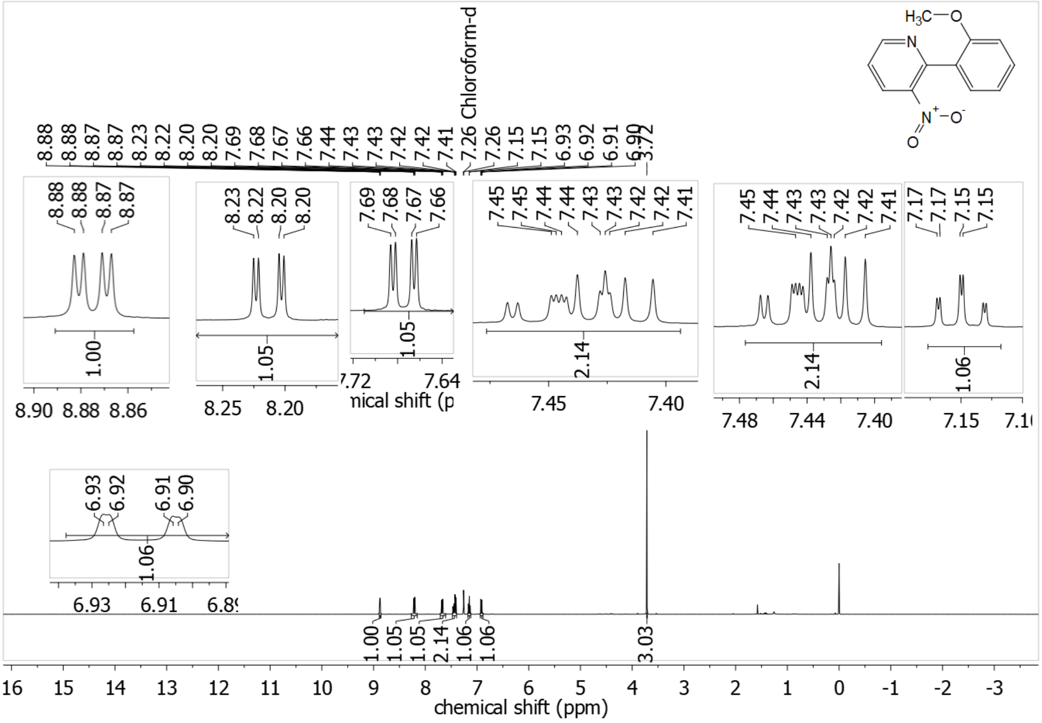

^{1}H NMR of **6e**

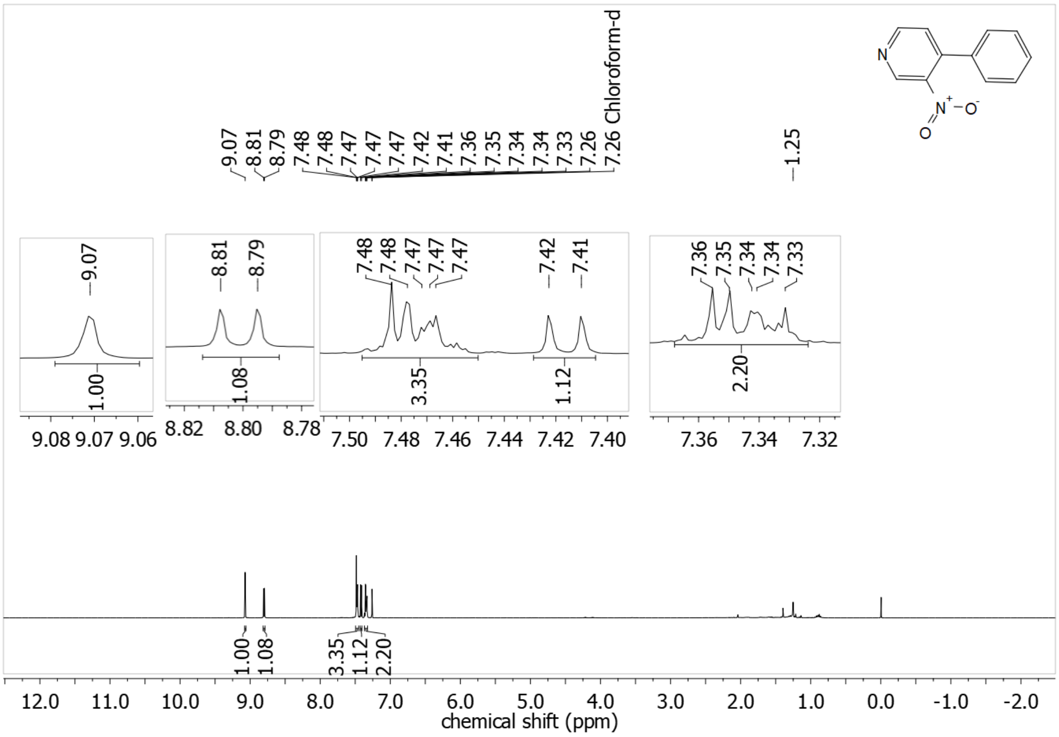

^{1}H NMR of **9**

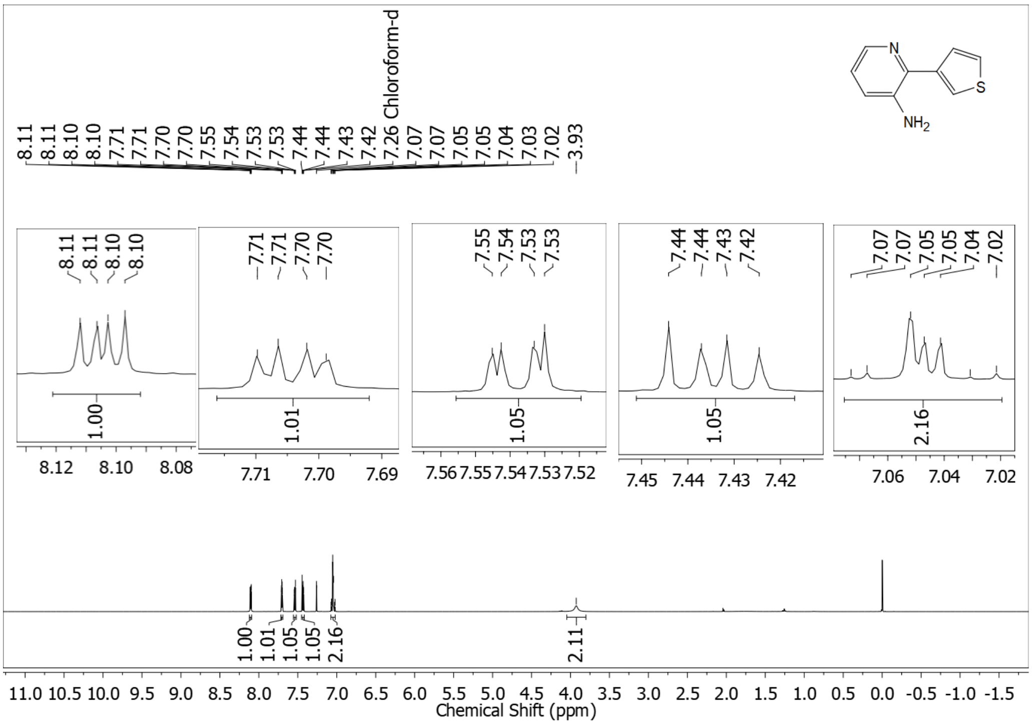

^{13}C NMR of **9**

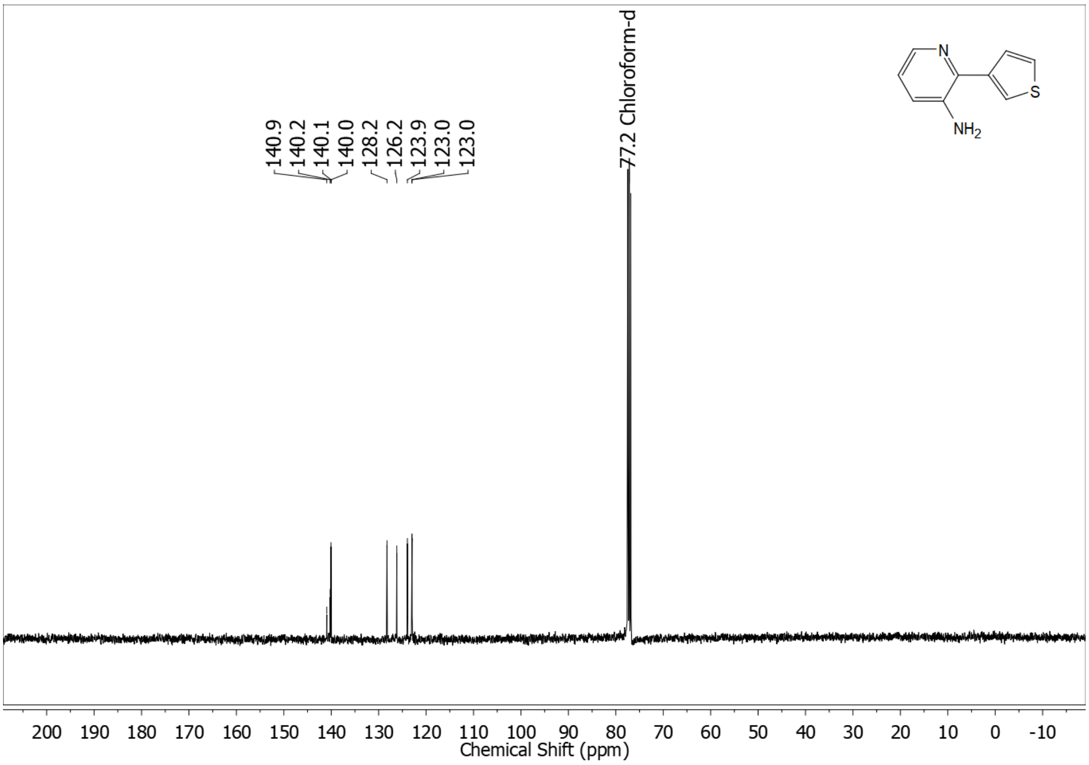

^{1}H NMR of **7a**

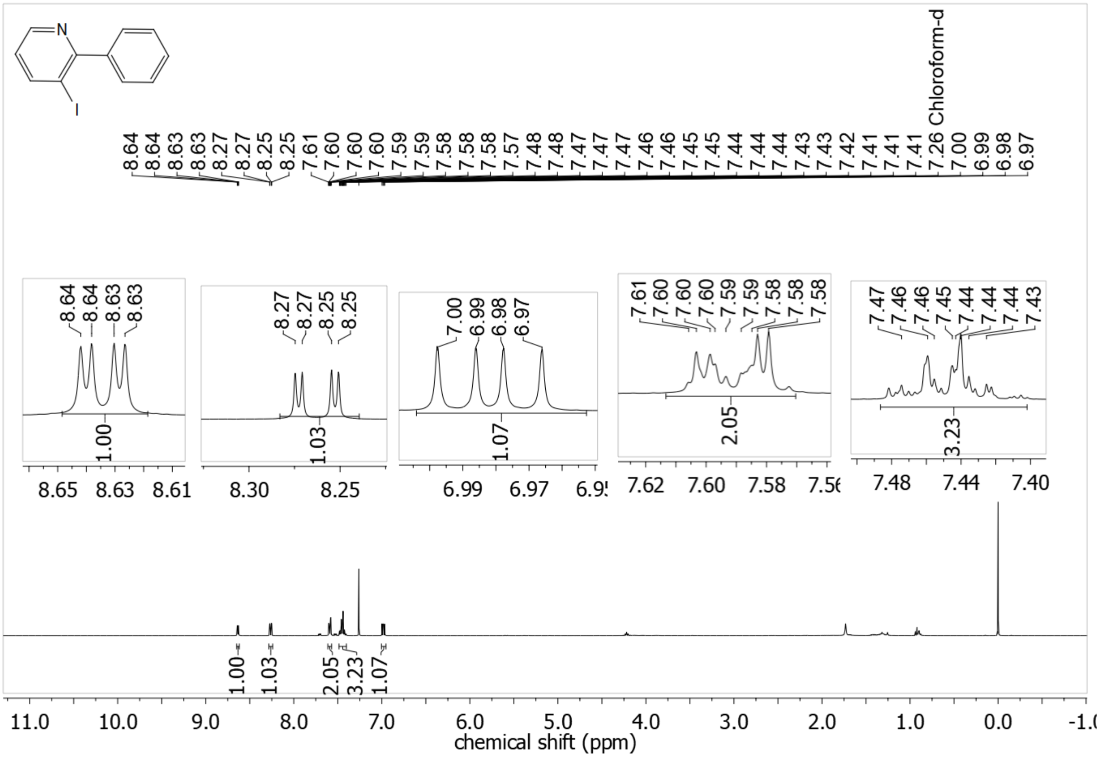

^{1}H NMR of **7b**

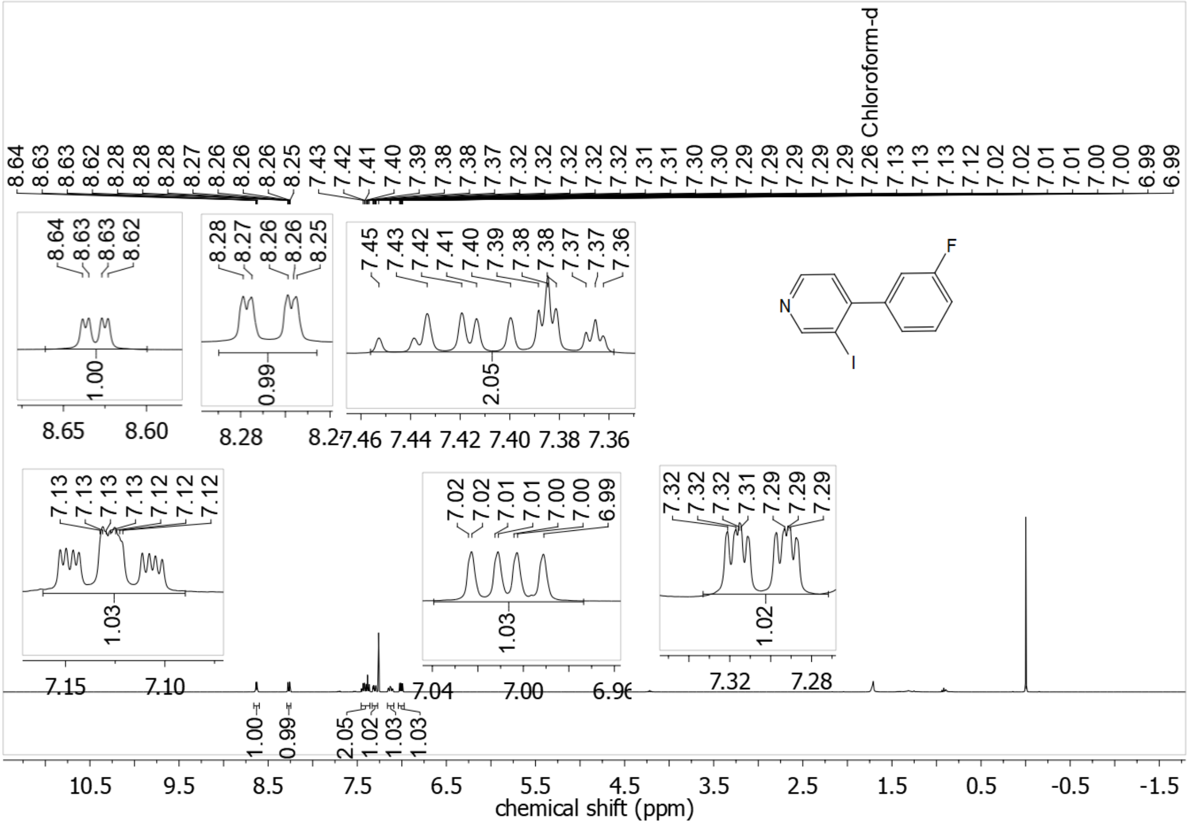

^{1}H NMR of **7c**

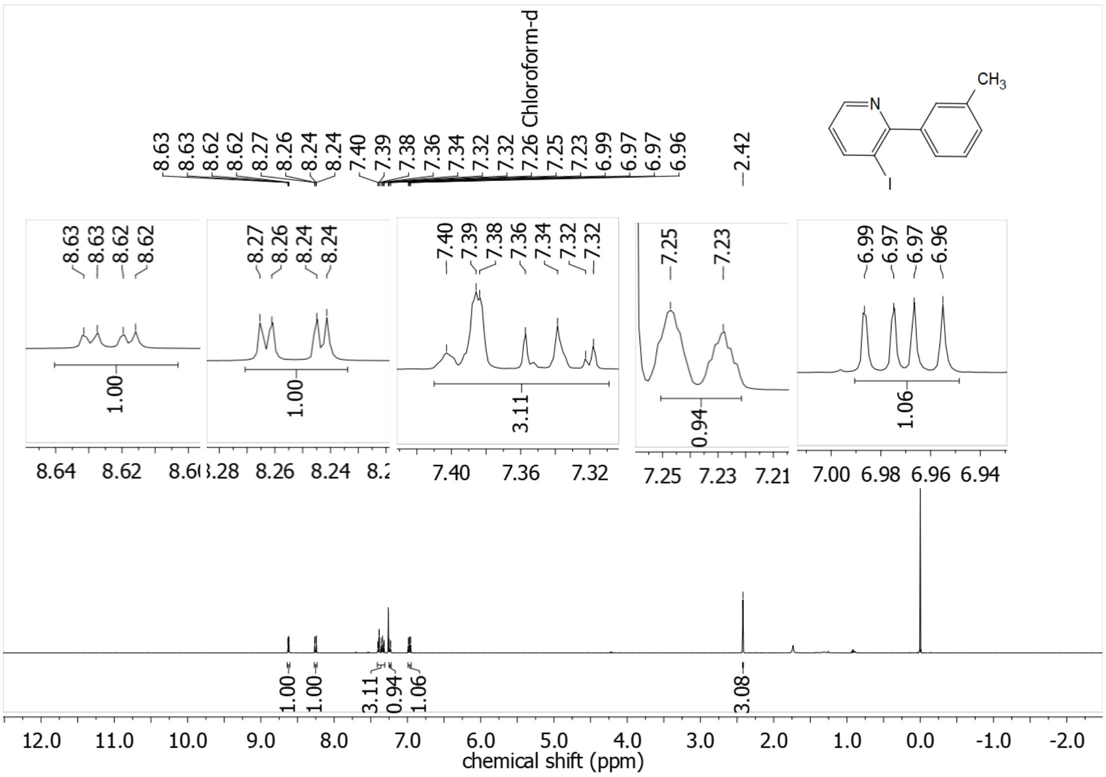

^{1}H NMR of **7d**

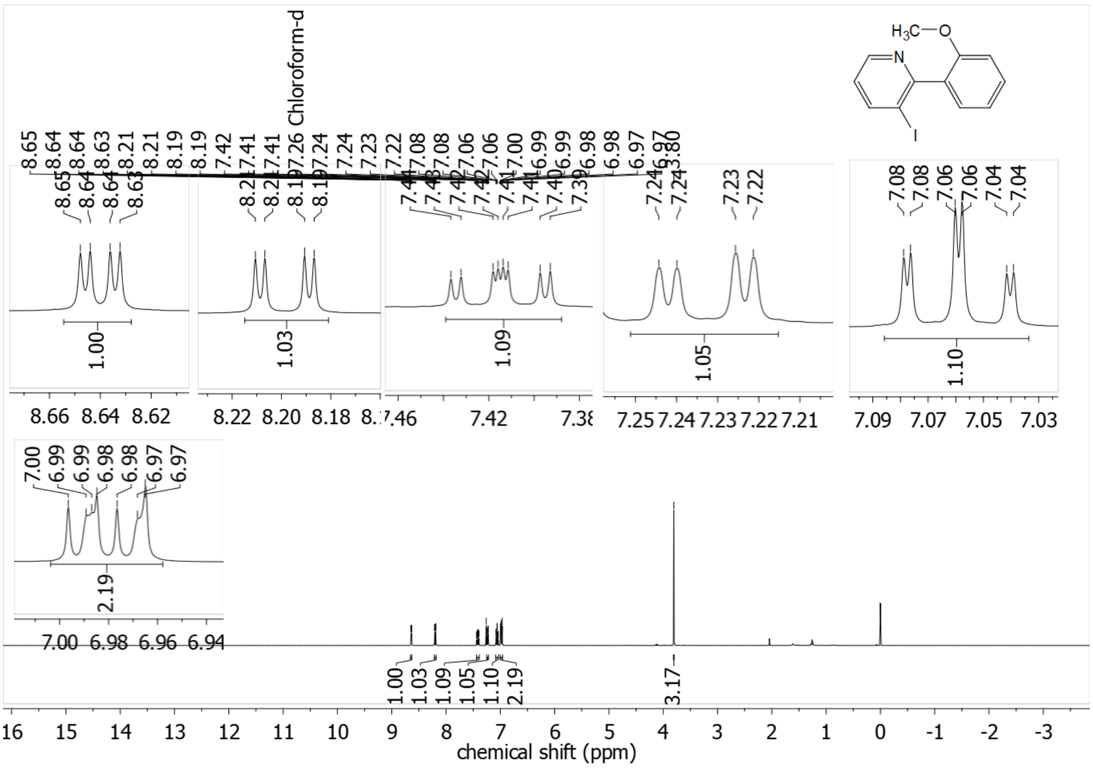

^{1}H NMR of **7e**

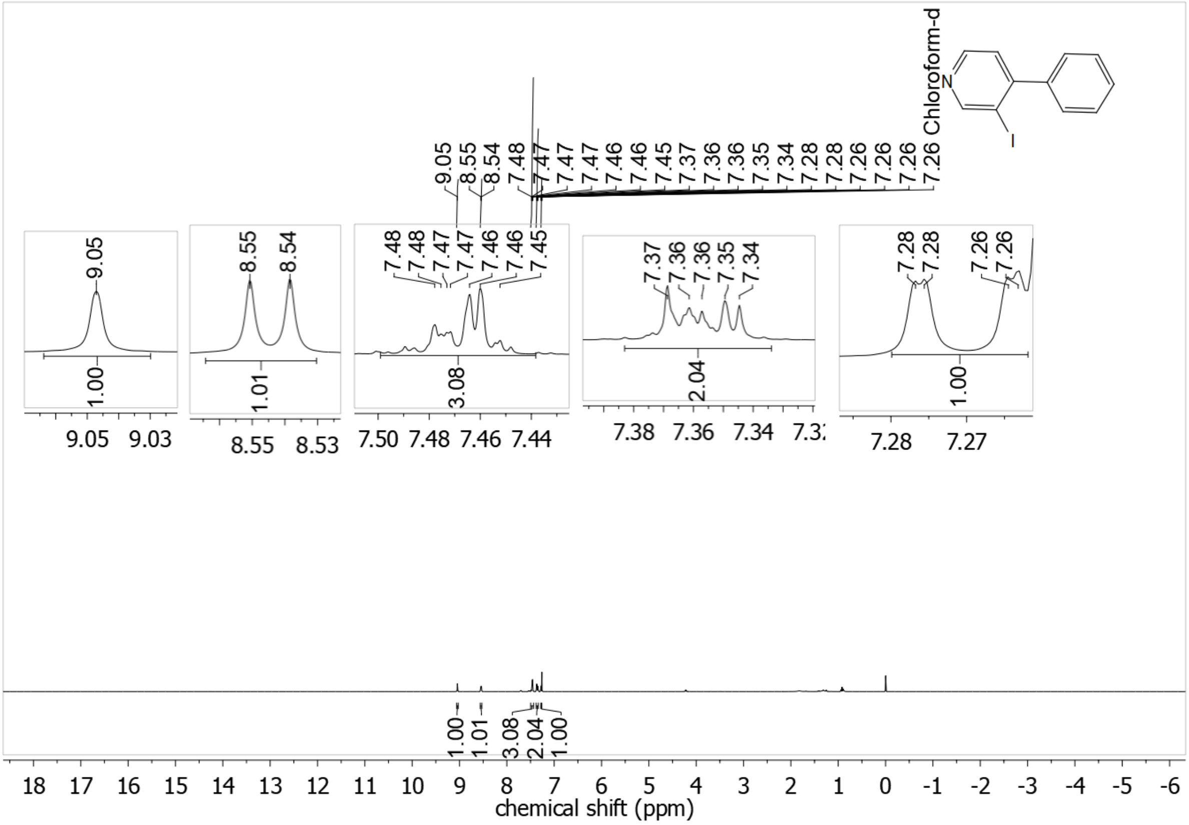

^{1}H NMR of **10**

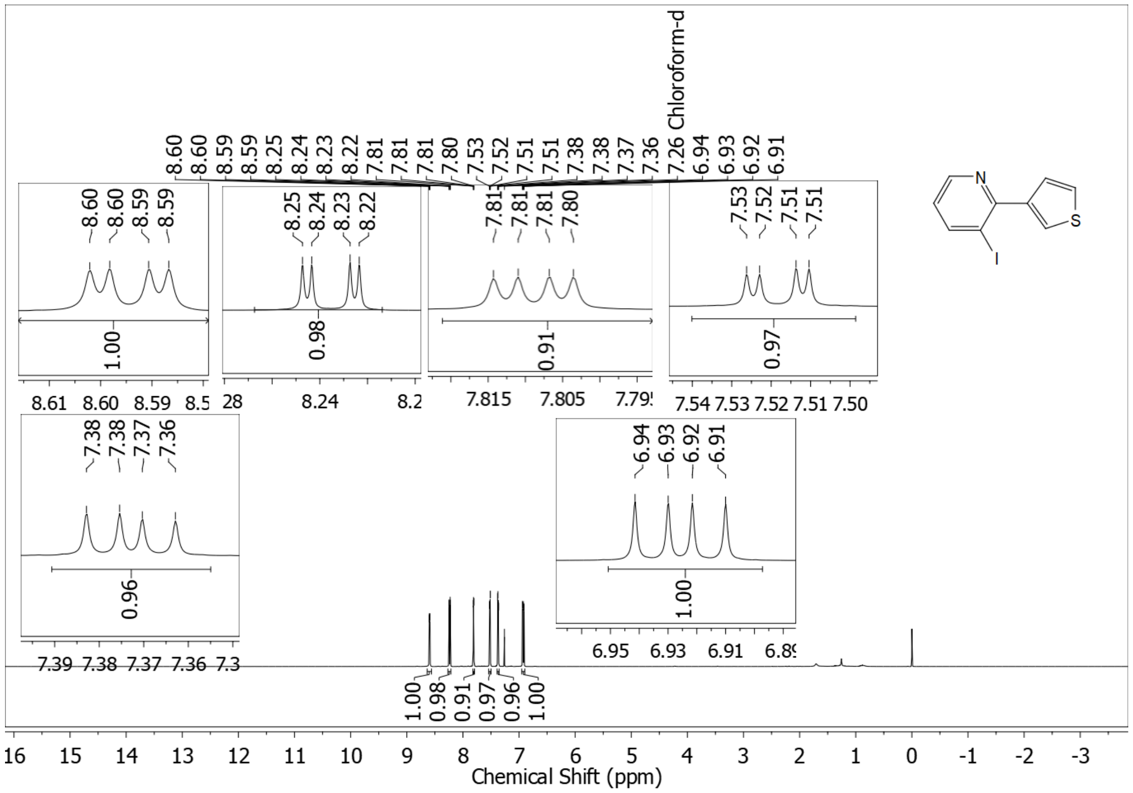

^{13}C NMR of **10**

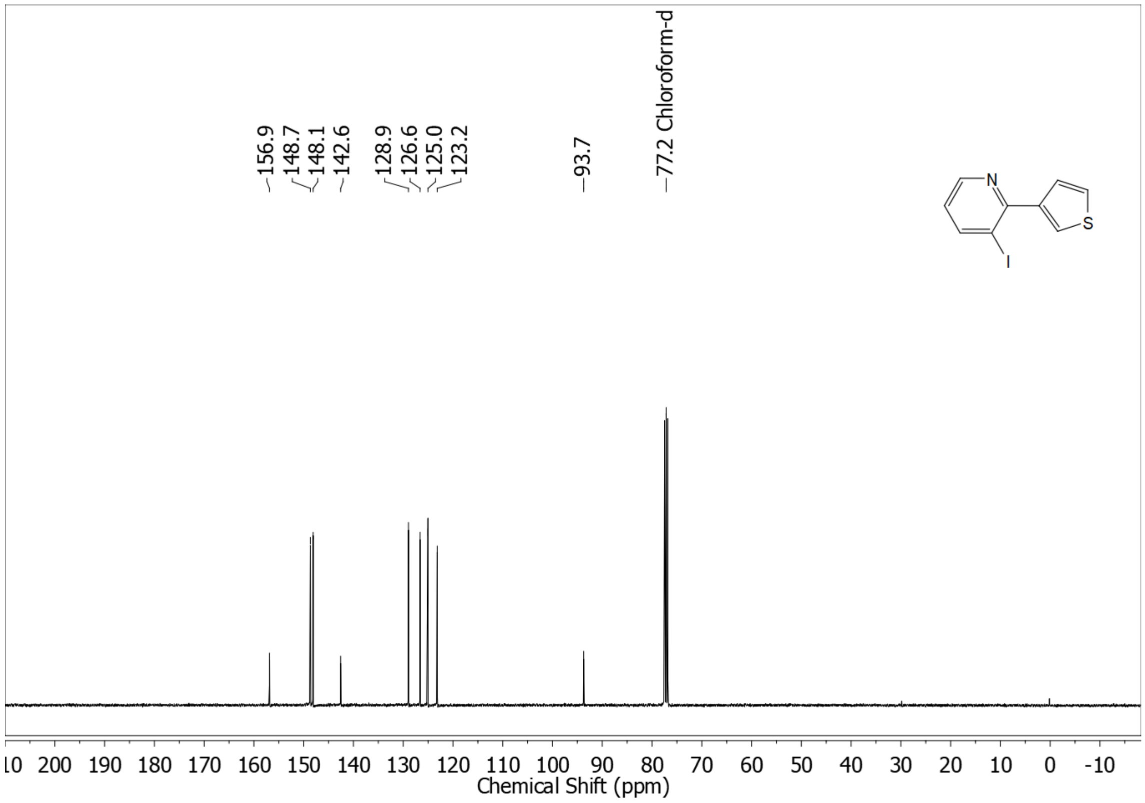

^{1}H NMR of **12**

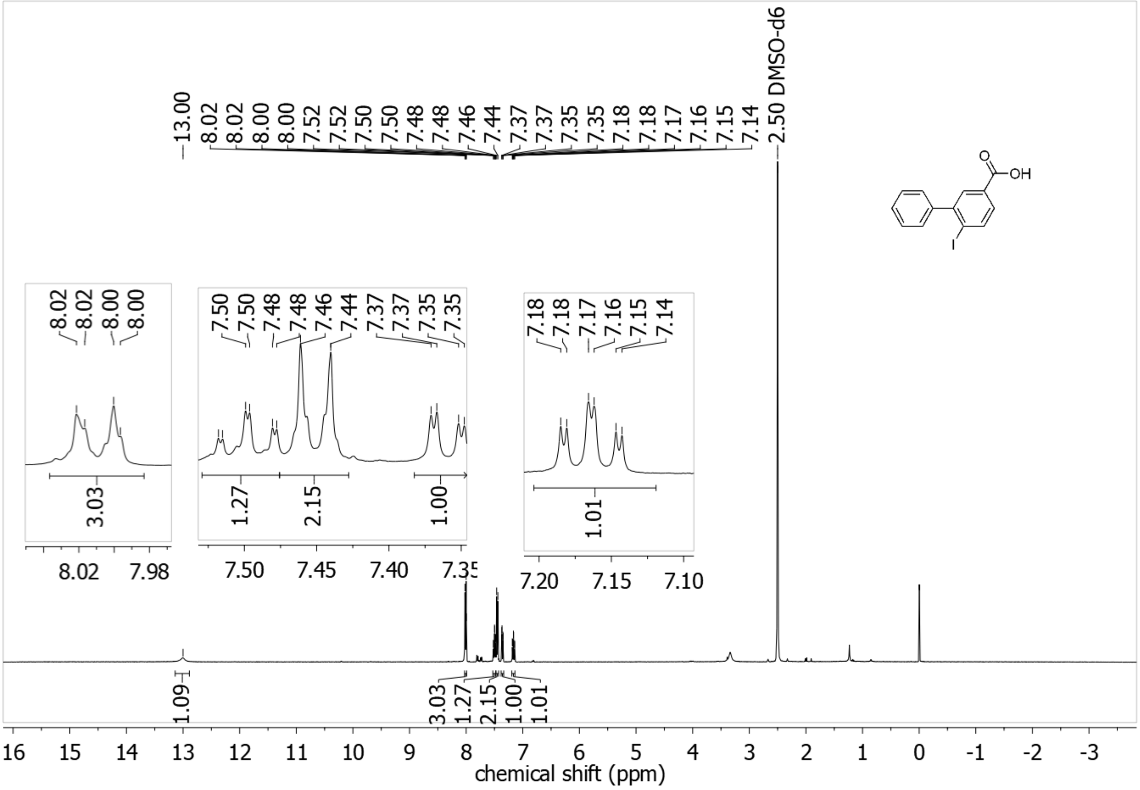

^{1}H NMR of **4**

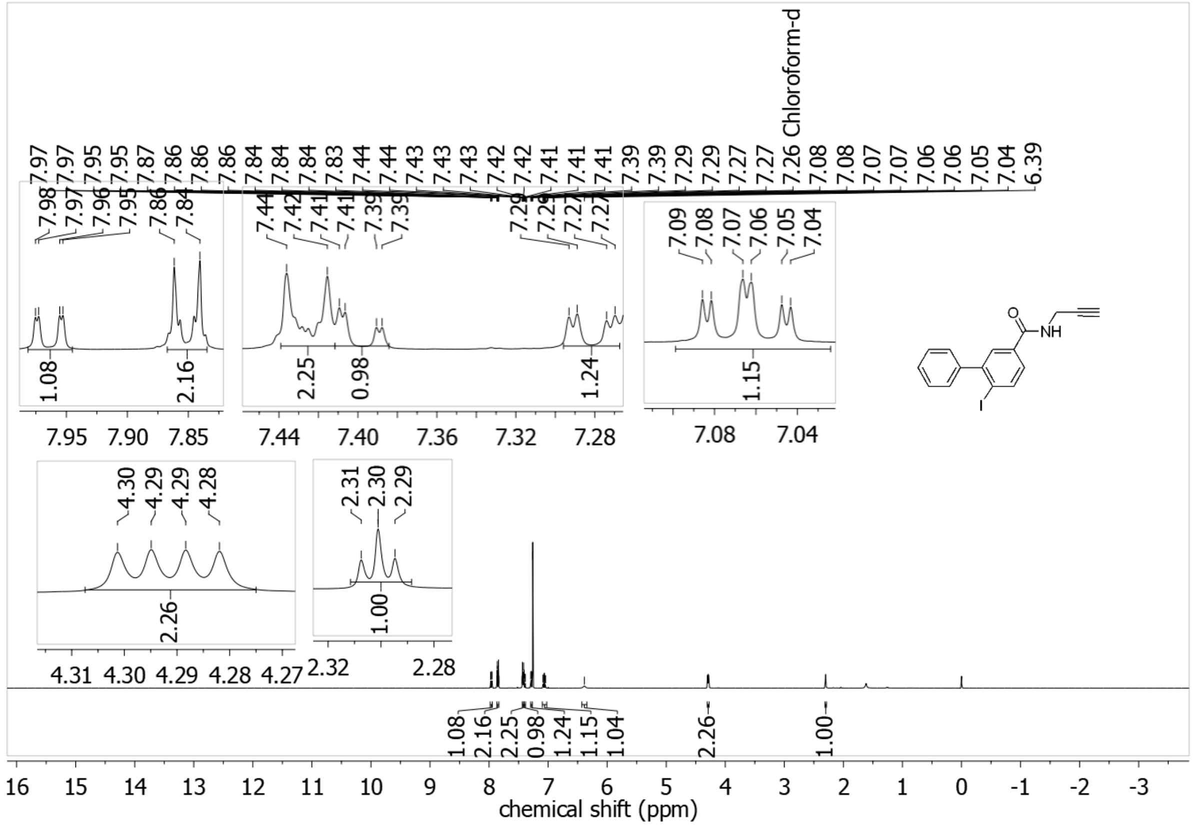

7.97 7.97 7.95 7.95 7.87 7.86 7.86 7.86 7.84 7.84 7.84 7.83 7.44 7.44 7.43 7.43 7.43 7.42 7.42 7.41 7.41 7.41 7.41 7.39 7.39 7.29 7.29 7.27 7.27 7.26 Chloroform-d 7.08 7.08 7.07 7.07 7.06 7.06 7.05 7.04 6.39
1.08 2.16 2.25 0.98 1.24 1.15 1.04 2.26 1.00
4.30 4.29 4.29 4.28
2.31 2.30 2.29
16 15 14 13 12 11 10 9 8 7 6 5 4 3 2 1 0 -1 -2 -3
chemical shift (ppm)

^{13}C NMR of **4**

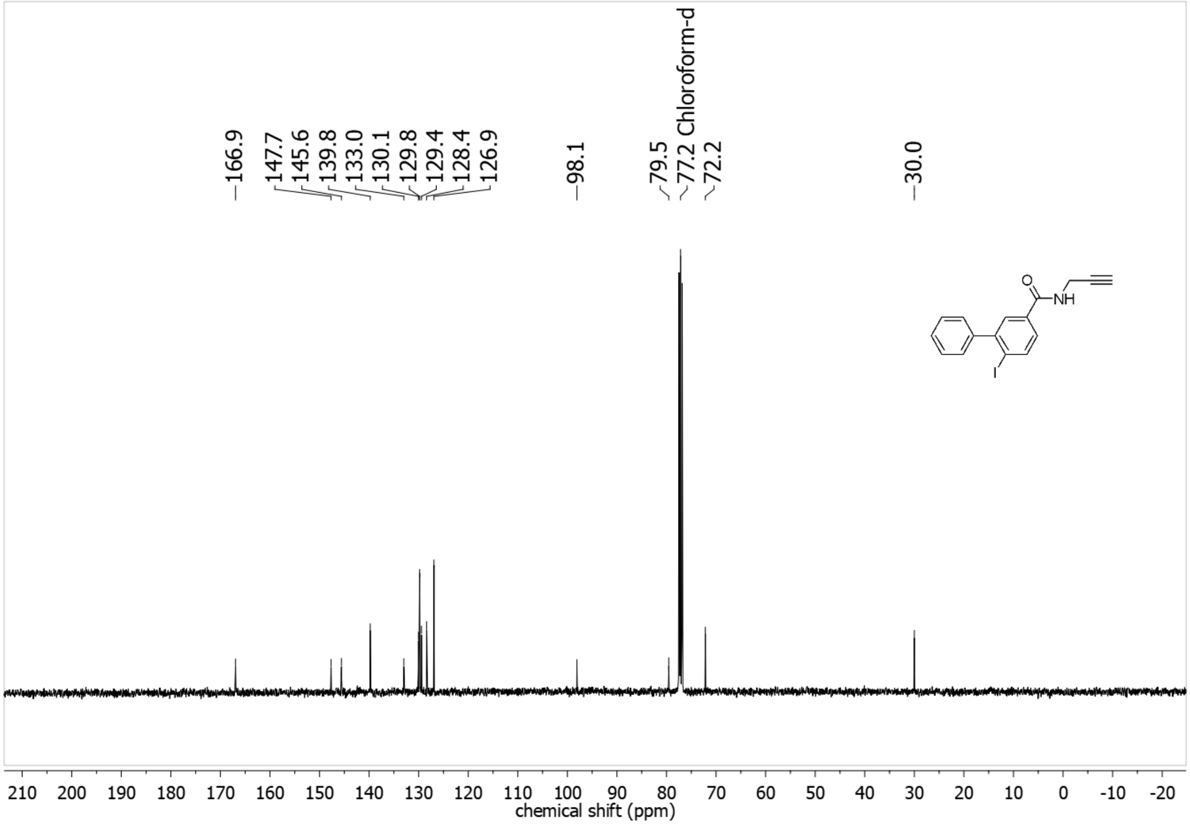

^{1}H NMR of **1a**

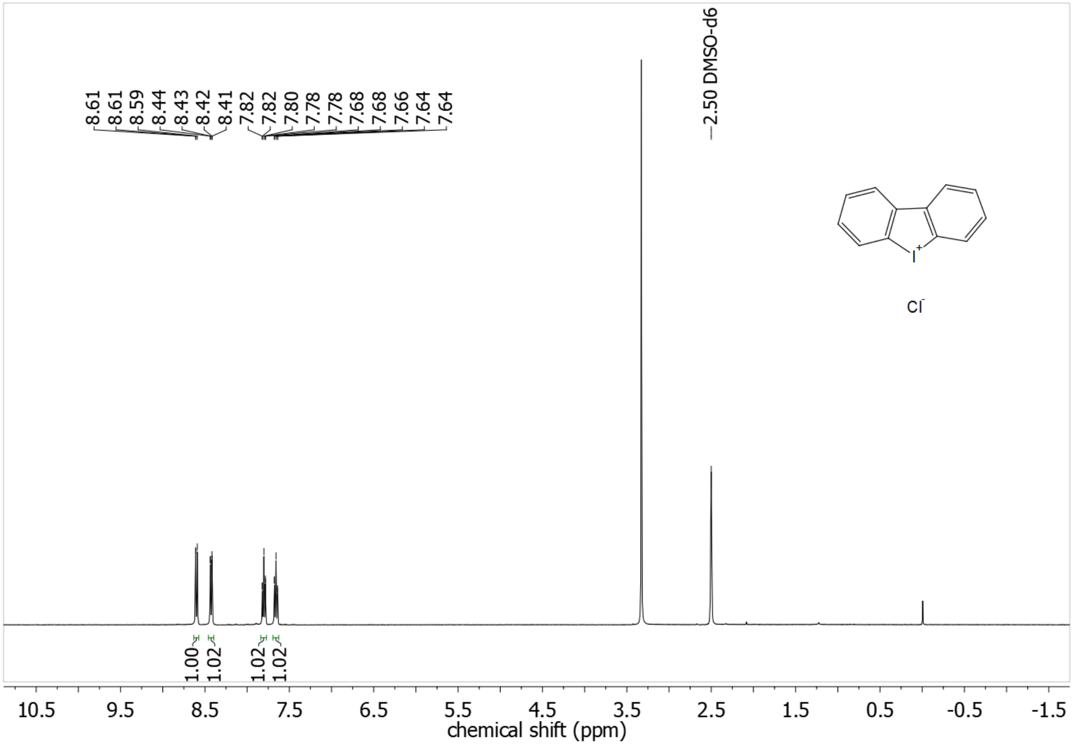

^{1}H NMR of **2a**

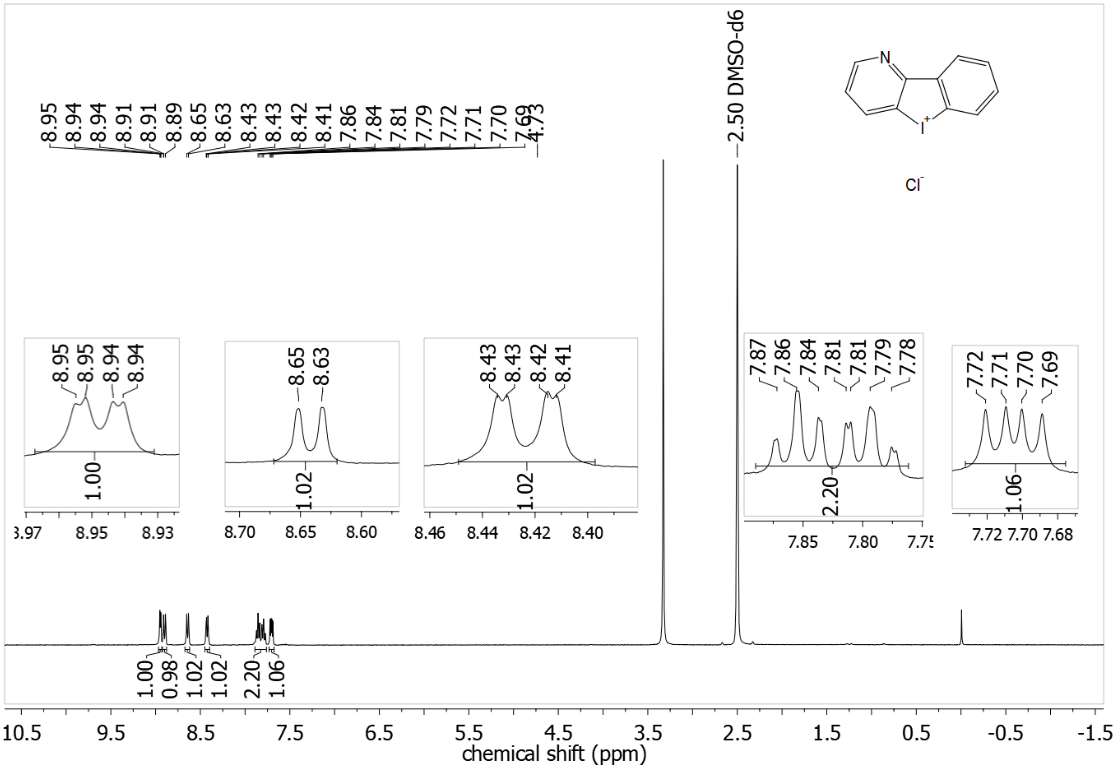

^{13}C NMR of **2a**

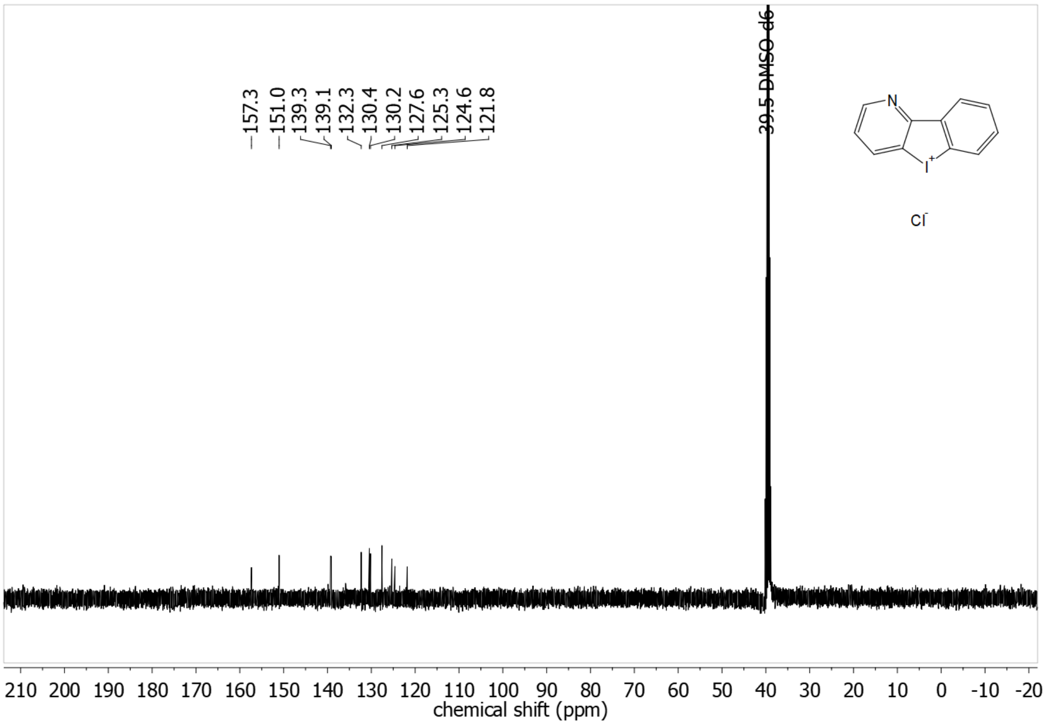

^{1}H NMR of **2b**

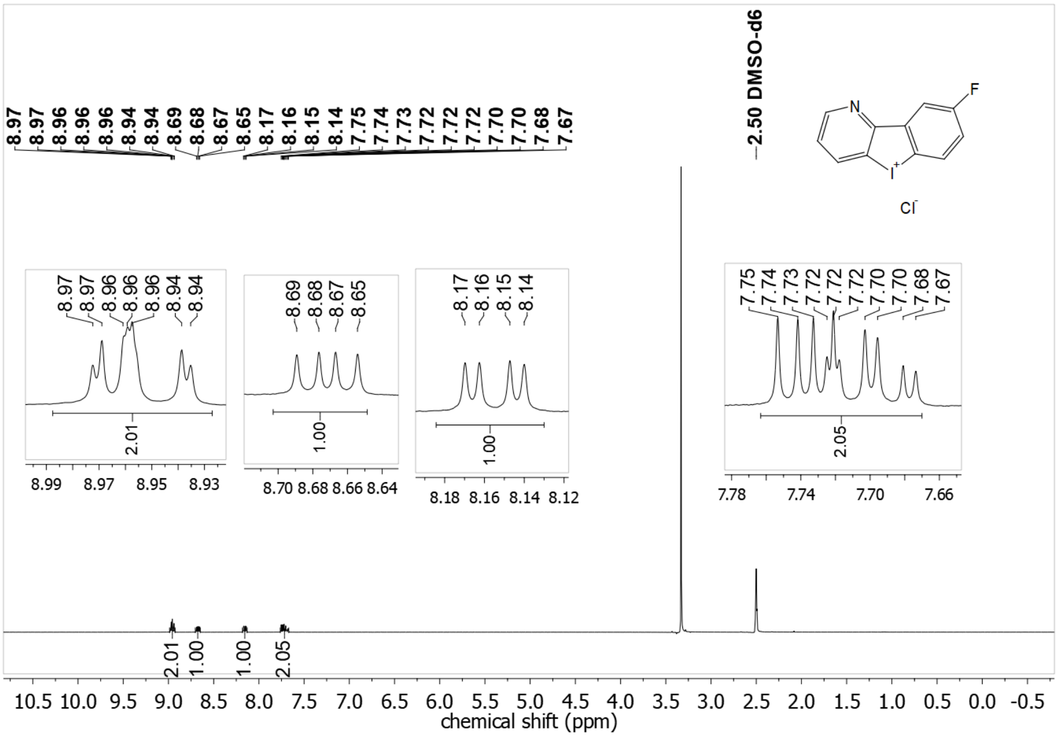

^{13}C NMR of 2**b**

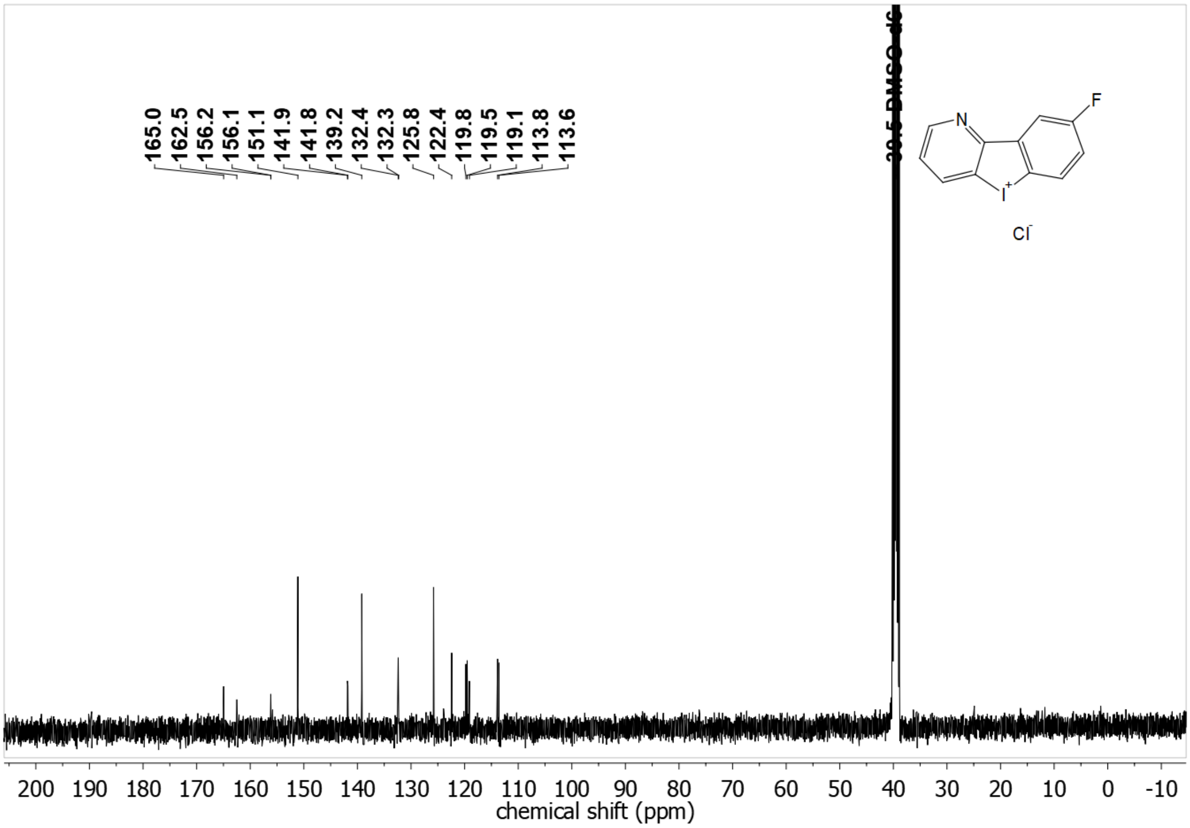

^{1}H NMR of 2**c**

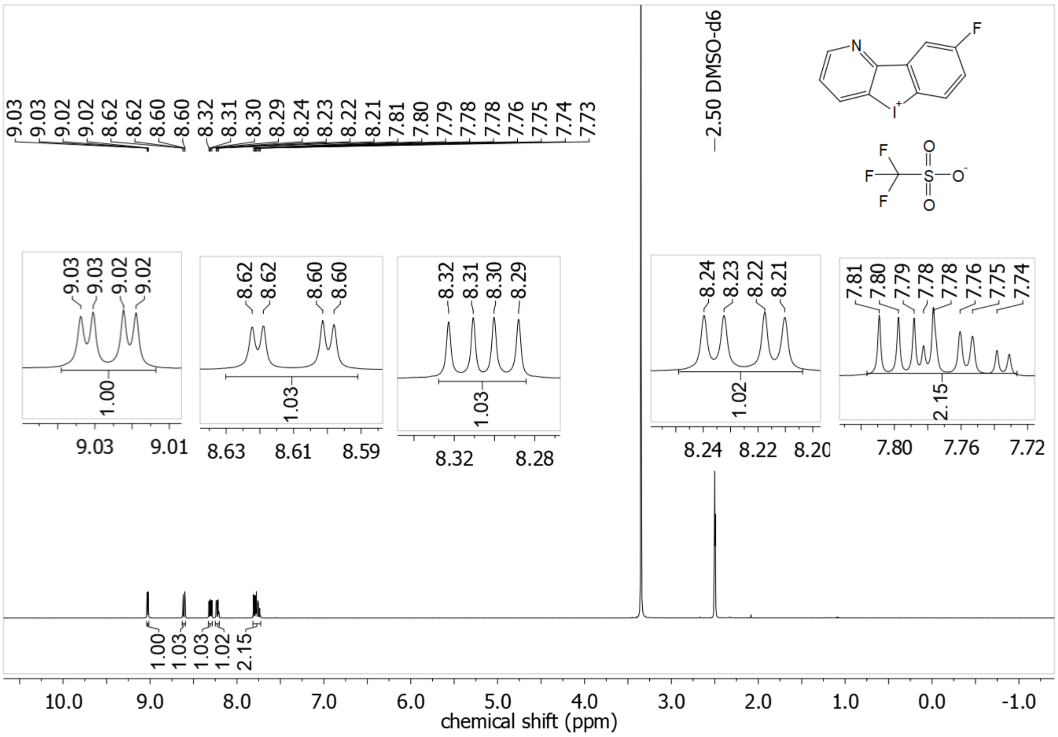

^{13}C NMR of 2**c**

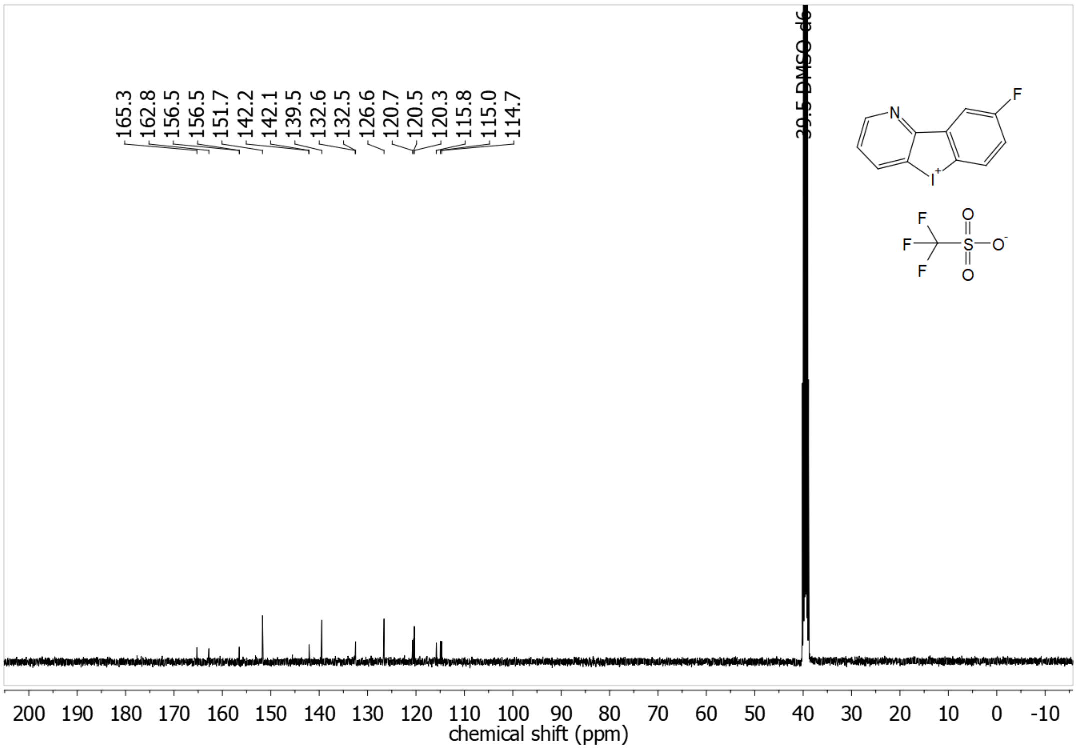

^{19}F NMR of **2c**

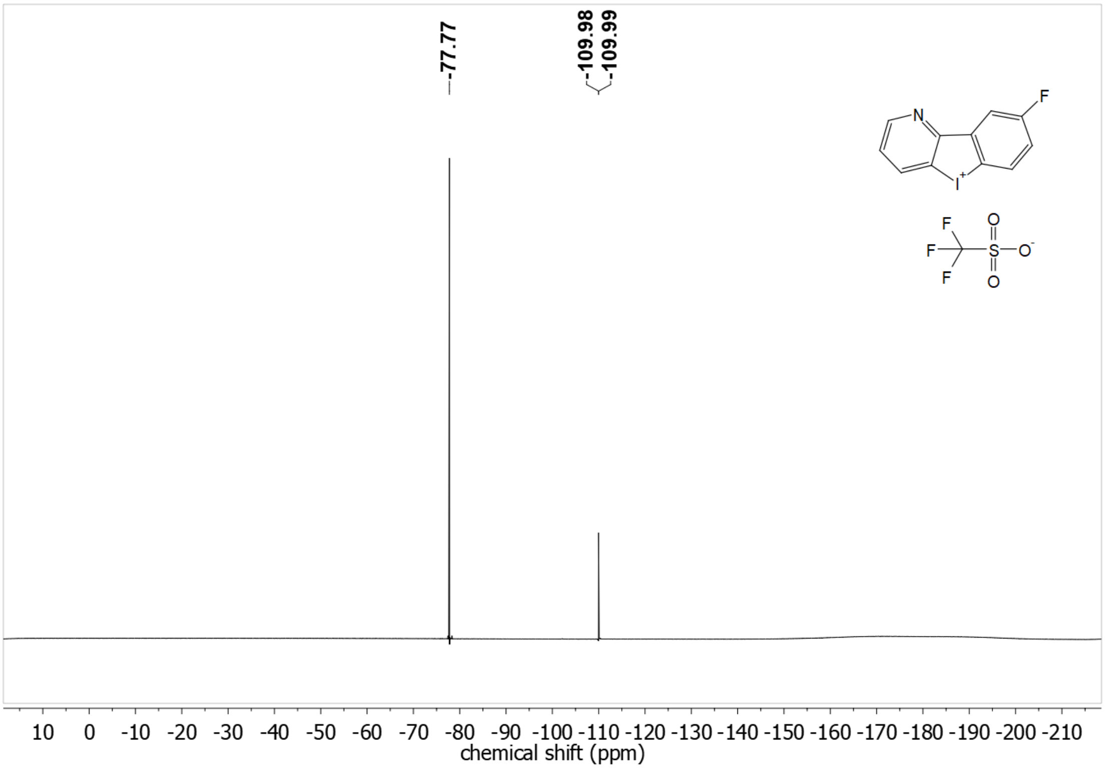

^{1}H NMR of **2d**

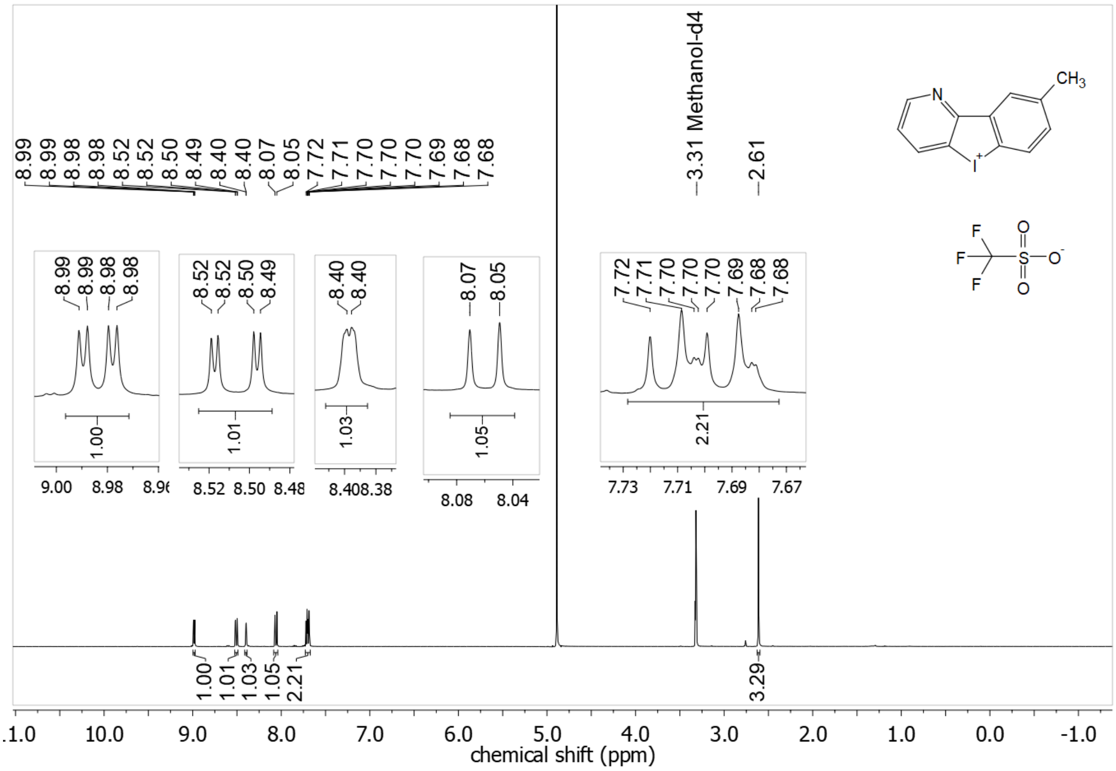

^{13}C NMR of 2**d**

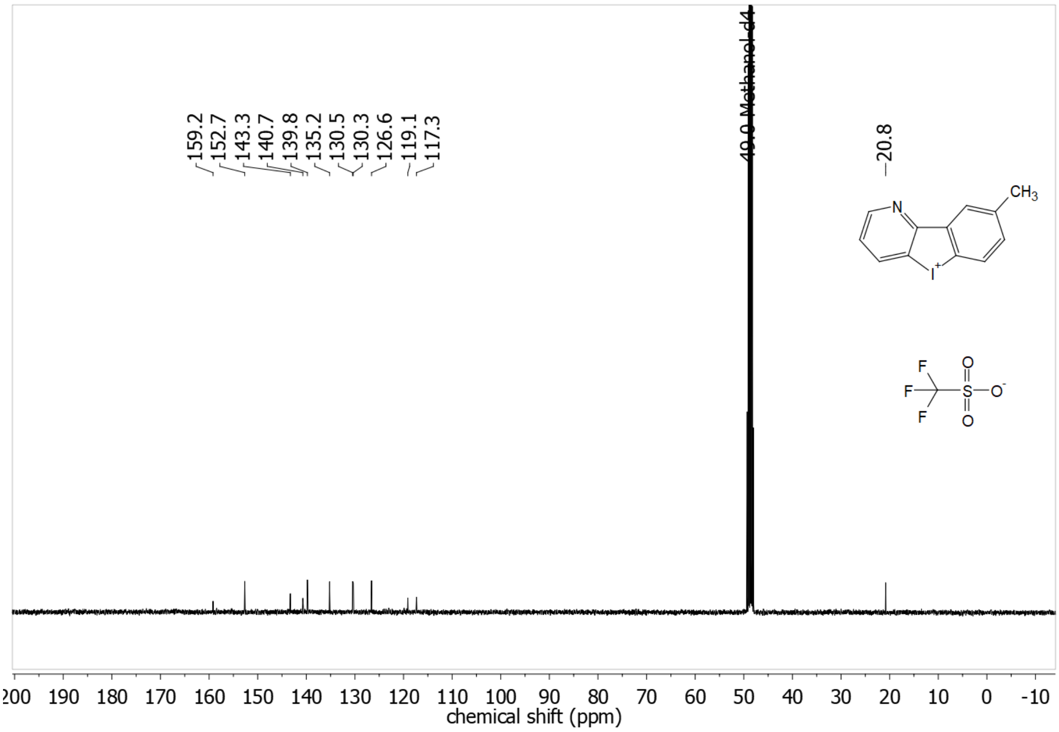

^{1}H NMR of **2e**

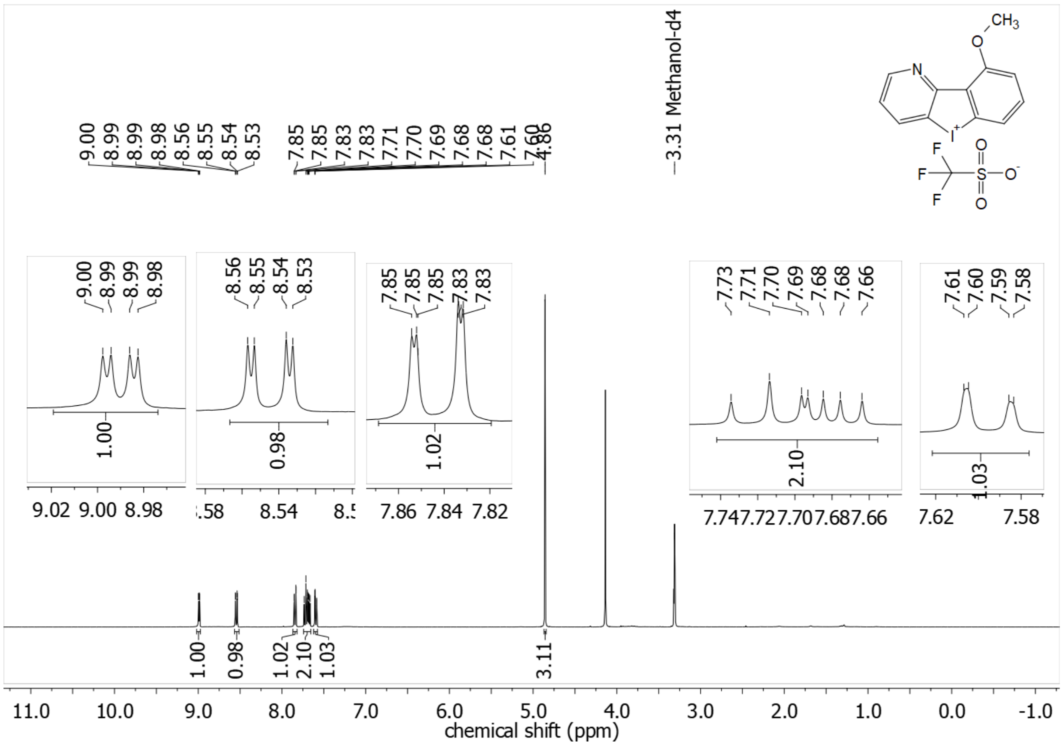

^{13}C NMR of **2e**

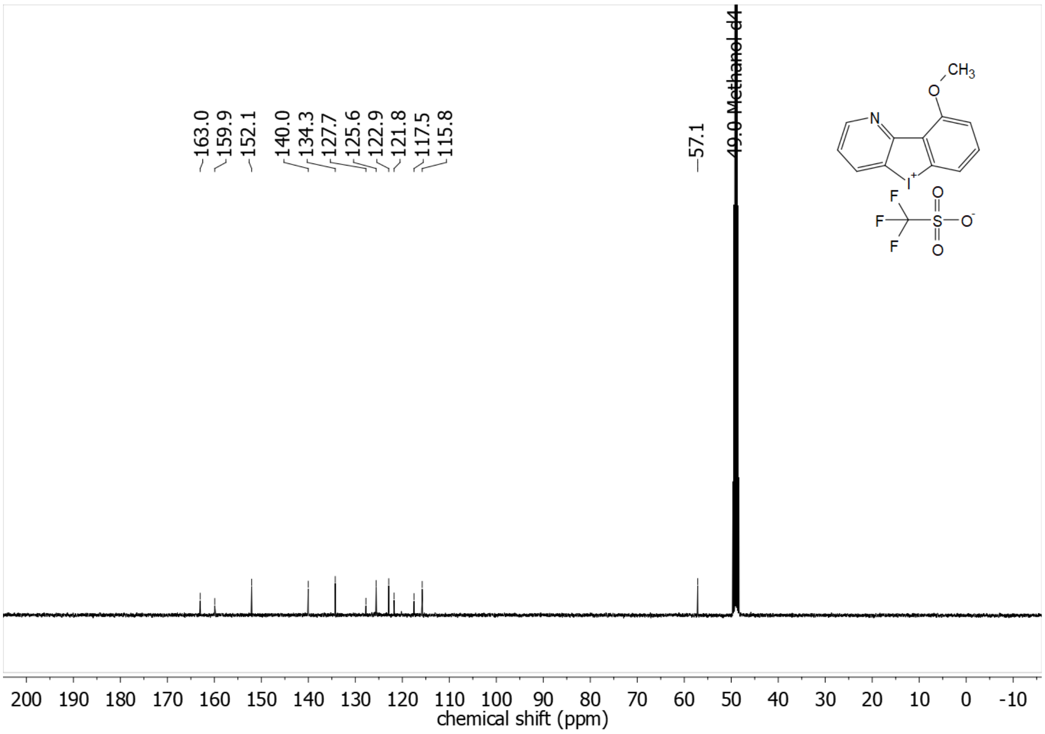

^{19}F NMR of **2e**

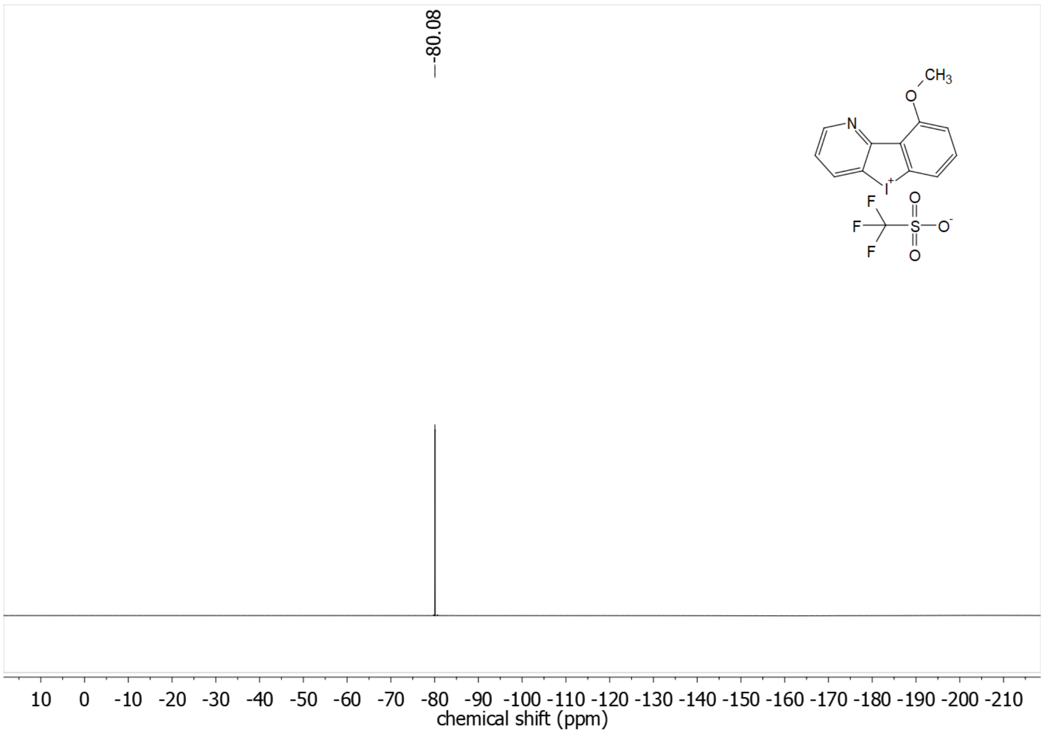

^{1}H NMR of **2f**

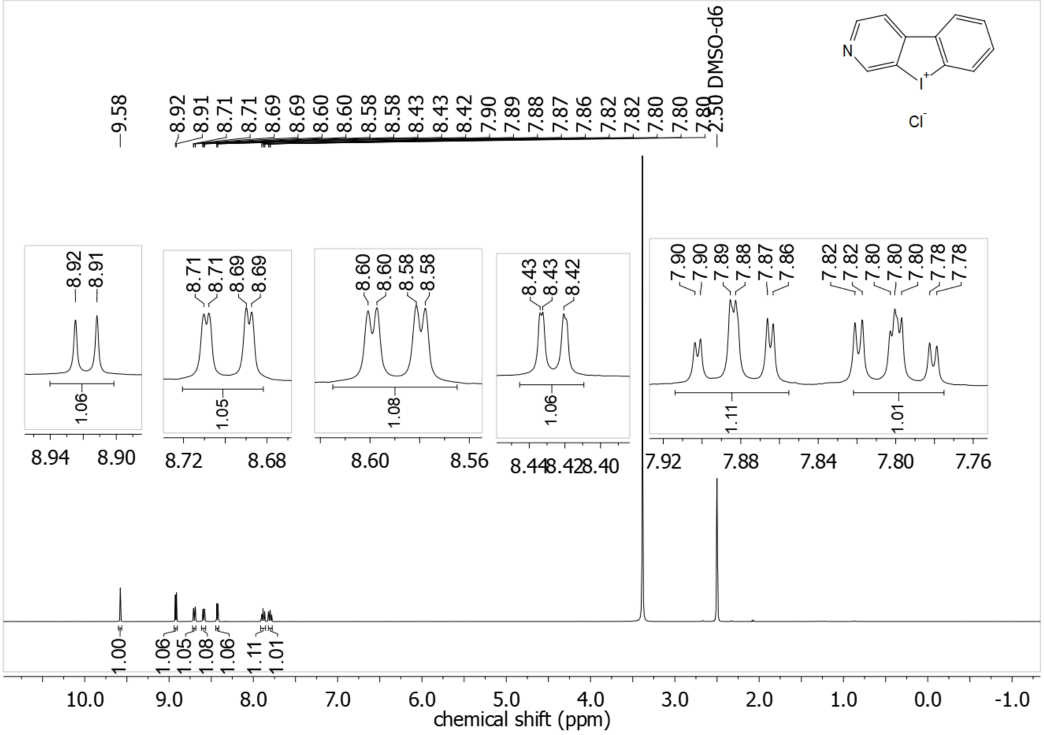

^{13}C NMR of **2f**

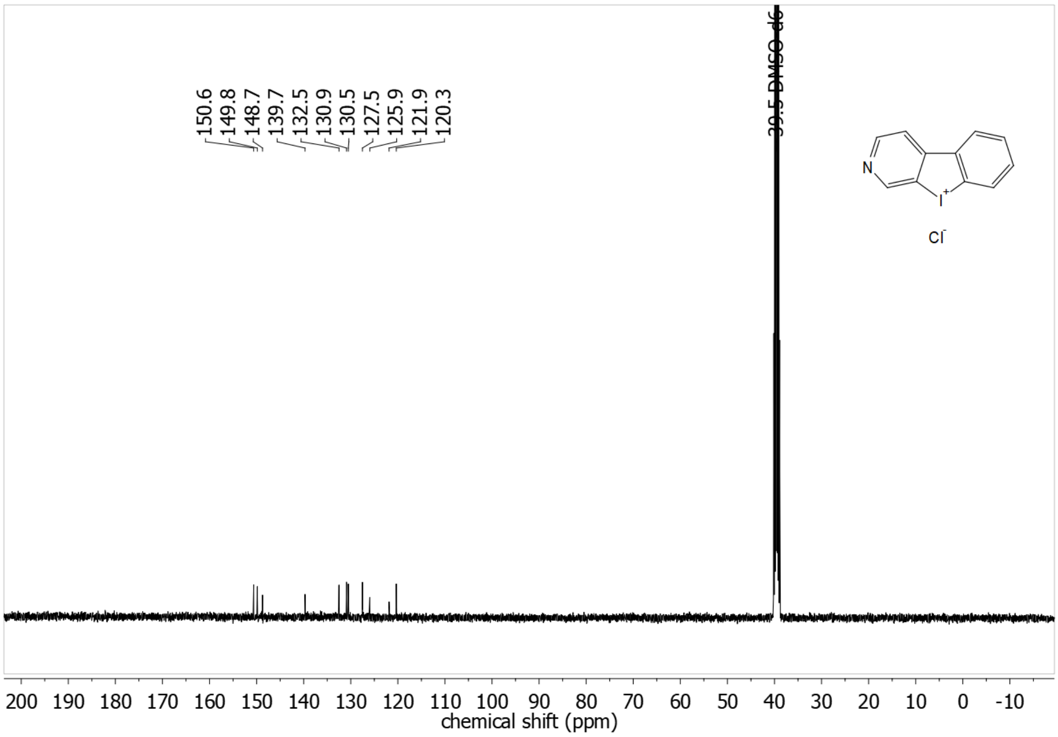

^{1}H NMR of **2g**

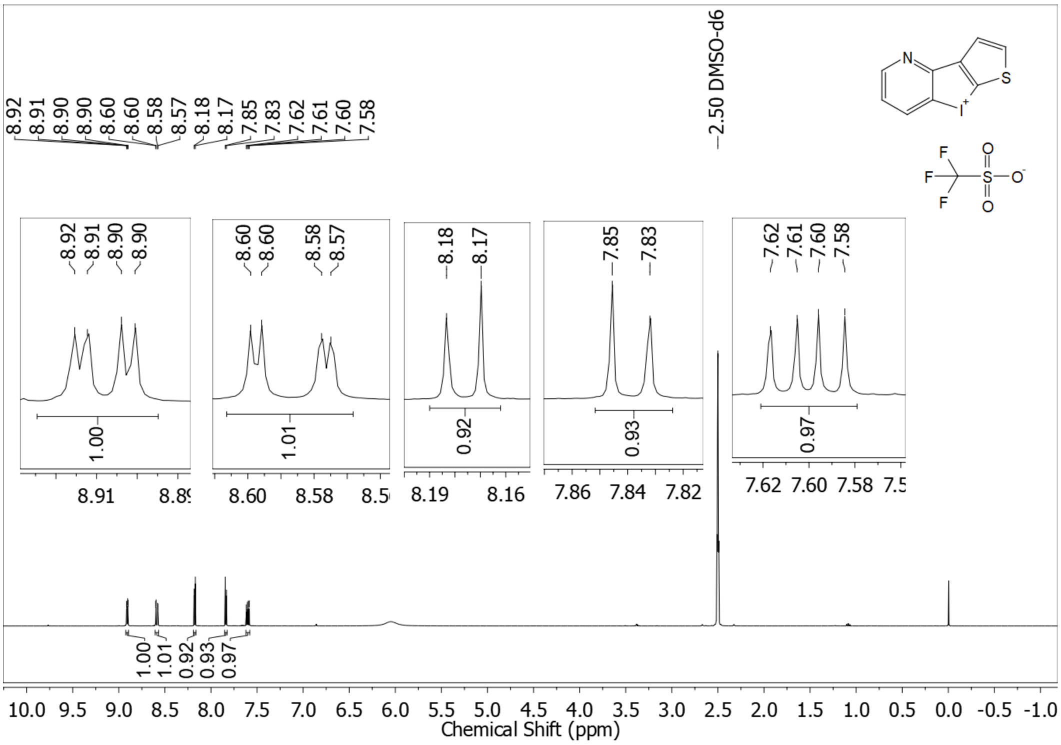

^{13}C NMR of **2g**

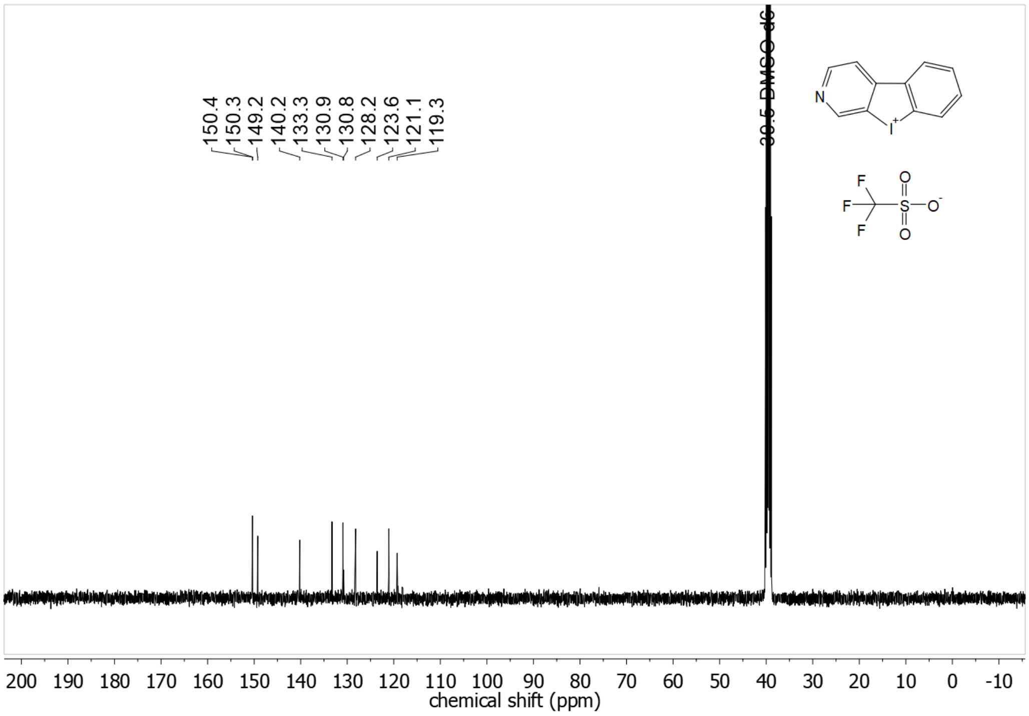

^{19}F NMR of **2g**

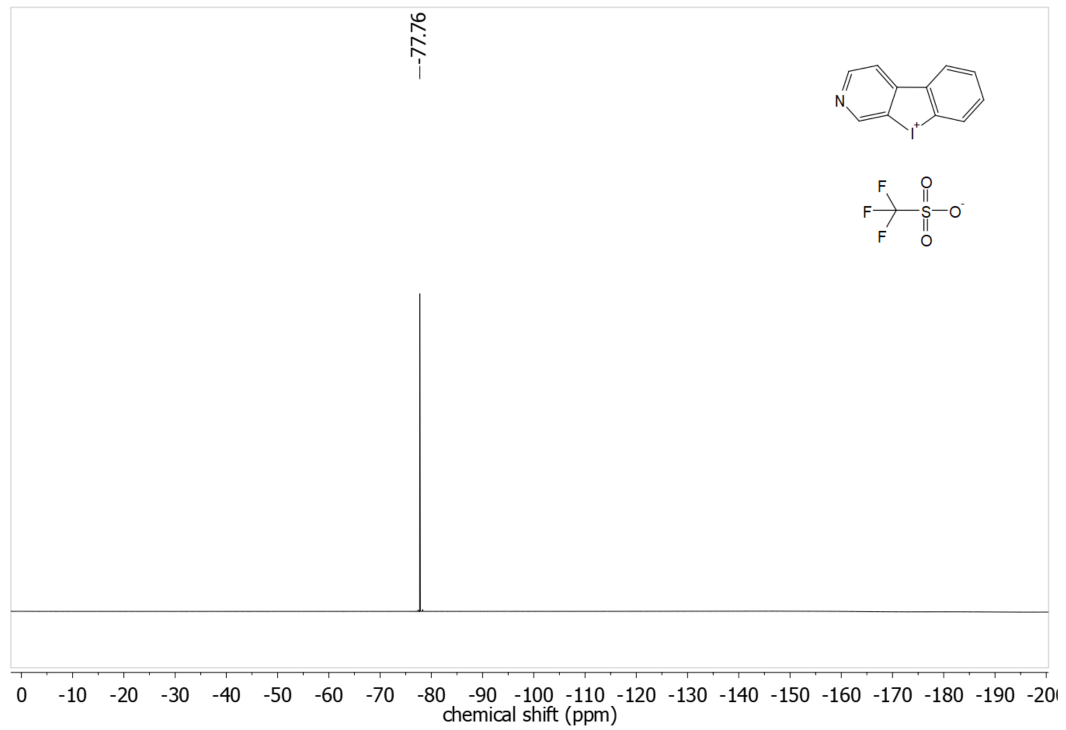

^{1}H NMR of **3a**

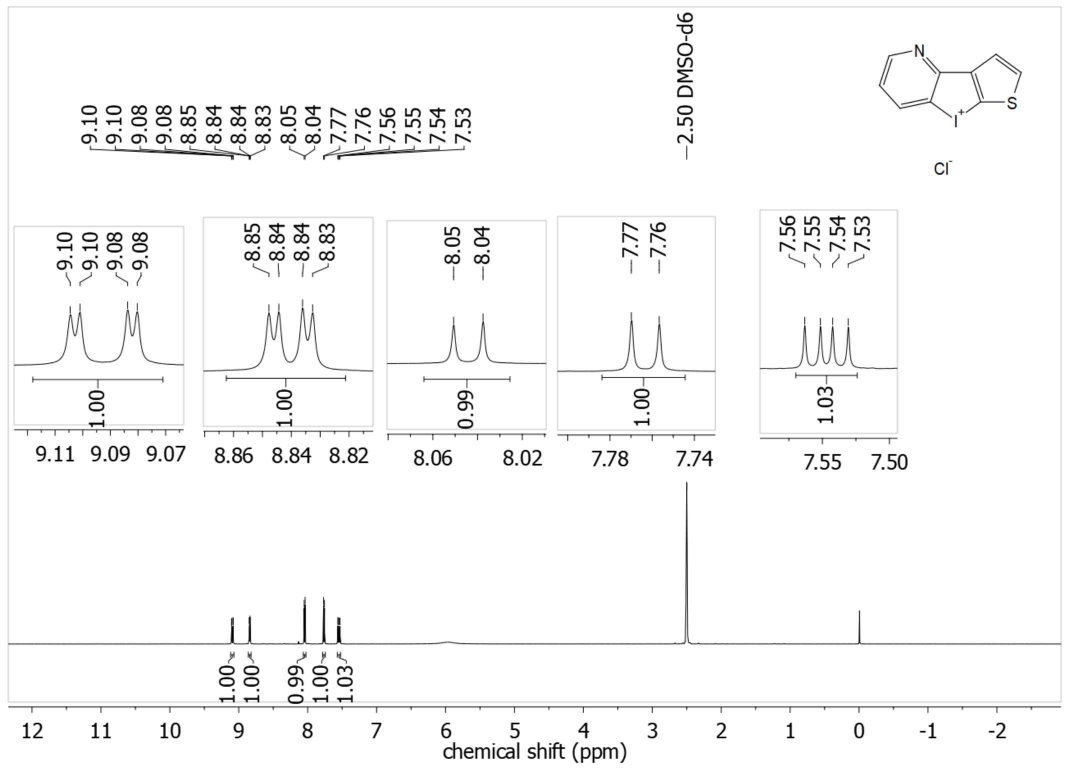

^{13}C NMR of **3a**

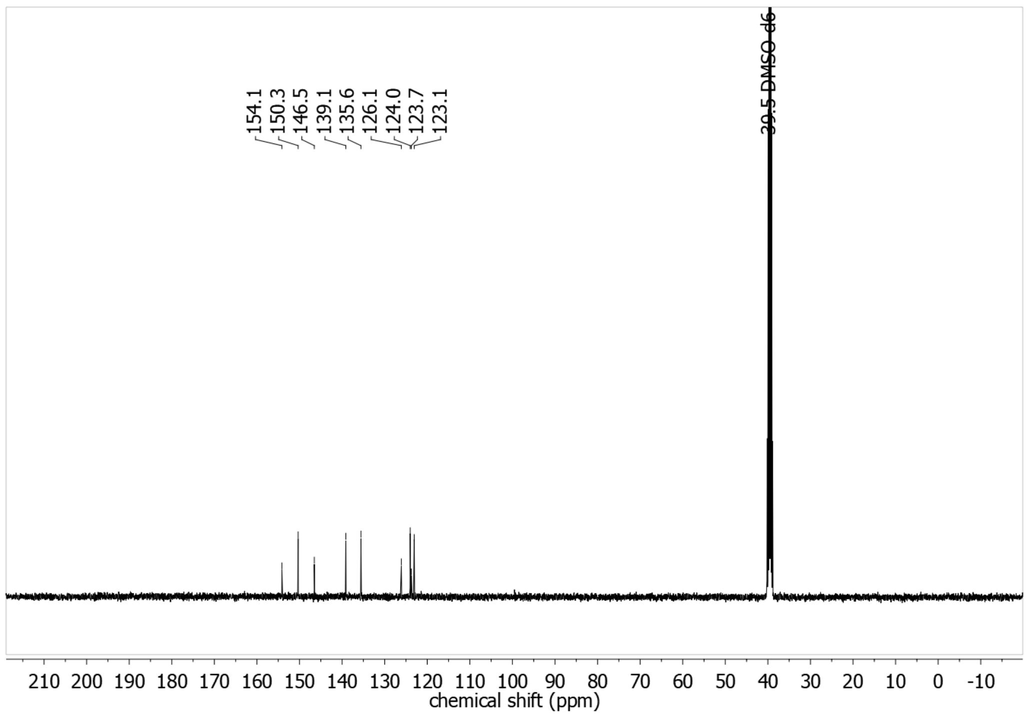

^{1}H NMR of **3b**

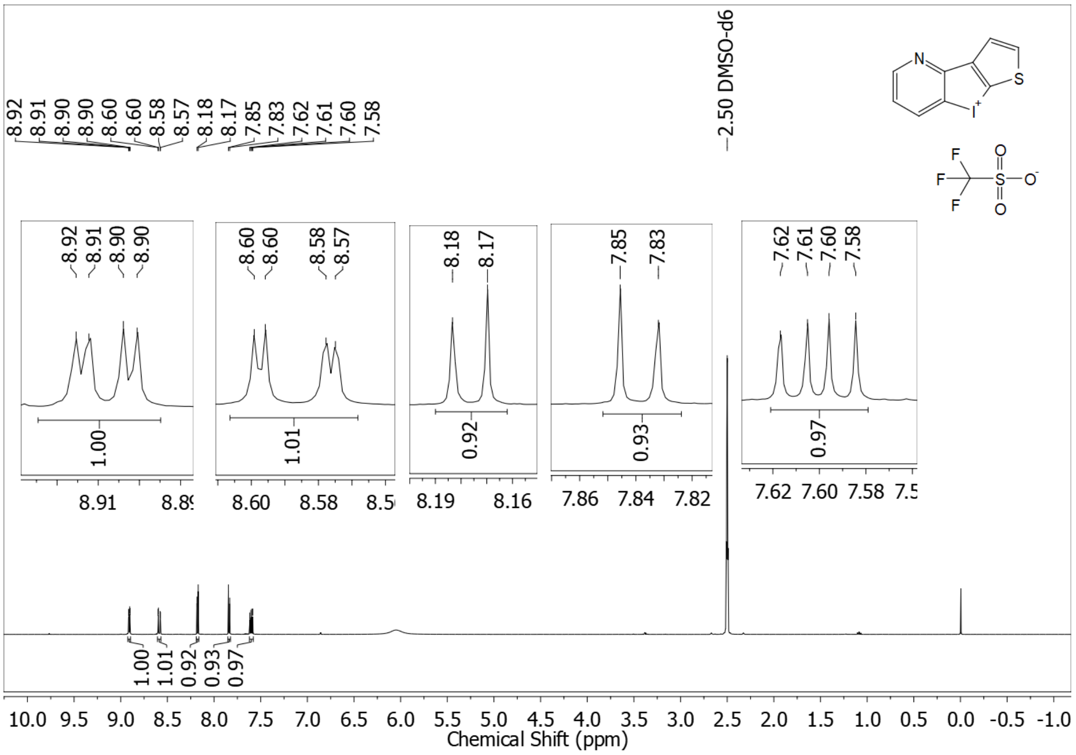

^{13}C NMR of **3b**

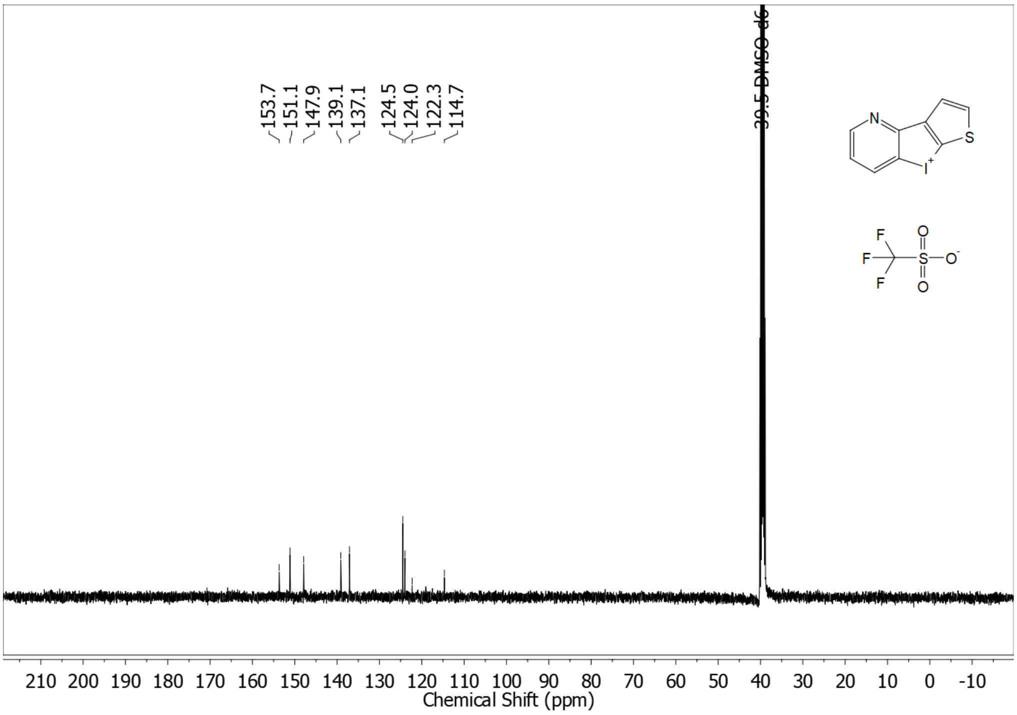

^{19}F NMR of **3b**

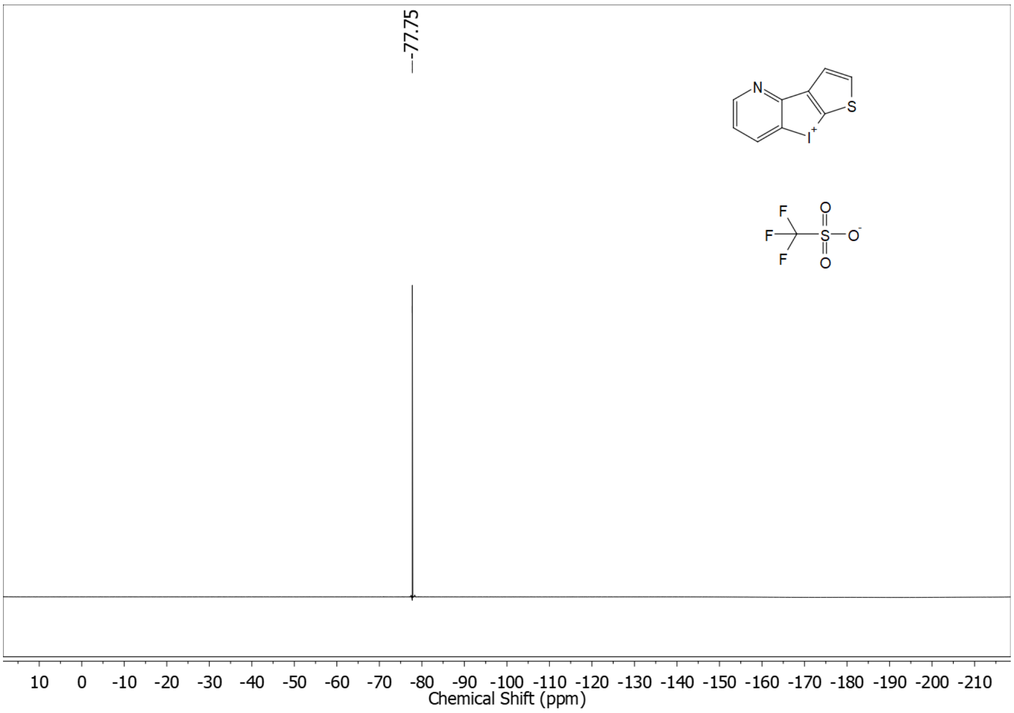

^{1}H NMR of **P1**

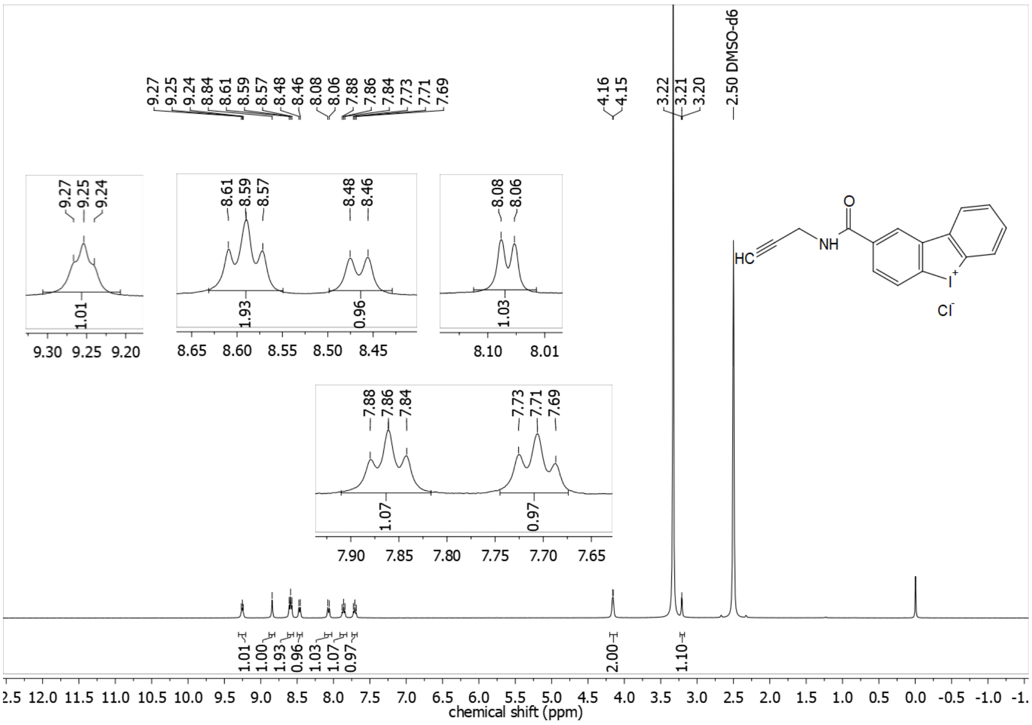

^{13}C NMR of **P1**

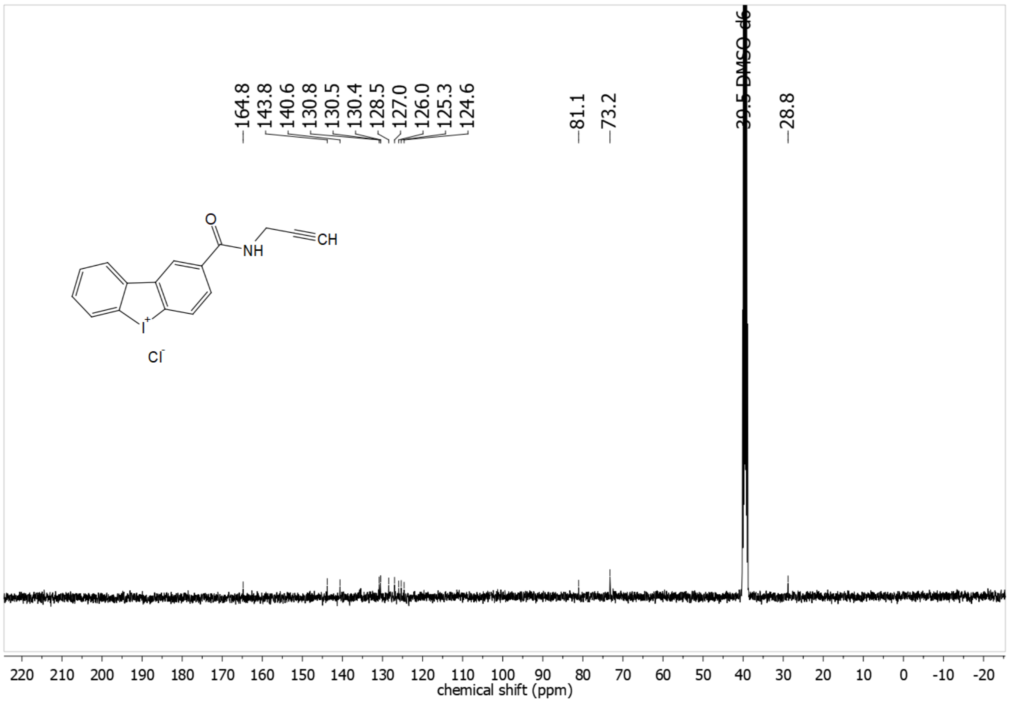

Supplement: Supplemental file 1 — Supplemental material. Download spectrum.04773-22-s0001.pdf, PDF file, 5.8 MB [file spectrum.04773-22-s0001.pdf]
